# A giant dapediid from the Late Triassic of Switzerland and insights into neopterygian phylogeny

**DOI:** 10.1098/rsos.180497

**Published:** 2018-08-15

**Authors:** Ashley E. Latimer, Sam Giles

**Affiliations:** 1 Paleontological Institute and Museum, University of Zurich, Zurich 8052, Switzerland; 2 Department of Earth Sciences, University of Oxford, Oxford OX1 3AN, UK

**Keywords:** Actinopterygii, CT scanning, holostei, durophagy, neurocranium, inner ear

## Abstract

A new Triassic neopterygian is described on the basis of a large three-dimensional neurocranium from the Rhaetian (Late Triassic) of the Kössen Formation (Schesaplana, Grisons, Switzerland). CT scanning reveals neurocranial features similar to *Dapedium*, suggesting that this new genus, *Scopulipiscis saxciput* gen. et sp. nov., was deep-bodied and potentially durophagous, although no associated dental material is known. An expanded phylogenetic analysis of actinopterygians resolves Dapediidae as a clade (inclusive of *Tetragonolepis*), although fails to recover any characters supporting the monophyly of the genus *Dapedium*. Dapediids are resolved as stem holosteans, filling a conspicuous gap in early neopterygian relationships. Pycnodonts, previously suggested as either stem teleosts or the sister group to dapediids, are resolved as a clade on the neopterygian stem. Similarities between the new taxon described here and *Dapedium* provide insights into morphological disparity within early members of the group—suggesting that the ecological expansion of dapediids originated prior to the End-Triassic extinction—as well as contributing to a growing understanding of endocranial anatomy in Palaeozoic and Early Mesozoic actinopterygians.

## 1. Introduction

Ray-finned fishes (Actinopterygii) constitute the majority of living vertebrate diversity, displaying a huge disparity of body forms filling a wide variety of ecological niches [1,2]. Almost all extant actinopterygians (more than 30 000 species) belong to a single group, the Teleostei, with non-teleostean actinopterygians comprising just 56 living species [3]. The distribution of extant species belies past diversity: teleosts have not always been the dominant group, having reached their current status only after the end of the Cretaceous [4,5]. Indeed, their sister group, the holosteans, outstripped teleosts in terms of diversity and disparity in the earliest part of their history [5], although only eight species survive in the modern. Despite this, the understanding of relationships among early holosteans remains poor, in particular with regard to taxa branching outside of the living radiation. A bare stem is typically recovered in most analyses (e.g. [6–9]), although a stem holostean identity has recently been suggested for *Dapedium* [10].

Representing an early experiment in body form and ecological diversity, *Dapedium* is a modestly diverse (approx. 20 species) radiation of neopterygians known from the Late Triassic (Rhaetian) to mid Jurassic (earliest Aalenian), most notable for its adaptations for durophagy. Members of this genus possess thick, interlocking ganoid scales; dense, pustular ornament; and a small mouth with styliform teeth borne on the stout lower jaw. Other genera closely associated with *Dapedium*, and typically united into Dapediidae (or Dapediiformes), include *Aetholepis* [11], *Dandya* [12], *Hemicalypterus* [13,14], *Heterostrophus* [15], *Paradapedium* [16], *Sargodon* [12] and *Tetragonolepis* [16,17], although the dapediid affinity of at least *Heterostrophus* and *Dandya* has been called into question [13,16]. Most *Dapedium* species have a standard length of around 15–35 cm [18], although dapediids range in size from approximately 8 cm (*Dapedium noricum* [12]) to approximately 1 m (*Sargodon tomicus* [12]). Only around five of the 30 or so known species of dapediid are present in the Triassic, and are exclusively represented by isolated teeth [19] or flattened [12,14,20] remains. Consequently, their adaptation for durophagy is thought to have facilitated the group's success in the aftermath of the End-Triassic extinction, a time at which many other durophagous taxa went extinct [21,22].

Despite representing an important expansion into a new ecological niche, the phylogenetic position of dapediids is unclear. Initially classed as ‘semionotids’ [12,23,24], a waste-basket taxon of primitive neopterygians, *Dapedium* was moved to Dapediidae by Lehman [25]. This family was erected largely on the basis of a deep-bodied phenotype [25–27], and a formal diagnosis was not provided until several decades later ([28]: these authors note that a previous diagnosis, formulated by Wenz [26], was largely inaccurate). Dapediids are poorly sampled in formal phylogenetic analyses (but see [14]), typically being represented by a single, often composite, terminal [3,29–37]. Despite—or perhaps in consequence of—their limited phylogenetic consideration, dapediids have variably been considered stem teleosts [8,29–31,34,35,38–40], total group holosteans (generally sister to ginglymodians [10,12,13,18,36,37]), and, most recently, stem holosteans [10]. Historical difficulties in defining apomorphies for dapediids stem from a lack of diagnoses teamed with characters of dubious merit (such as the distribution of scale serrations and multicuspid teeth [11]) being used to distinguish between species, and the monophyly of *Dapedium* has previously been questioned [28]. Furthermore, material from the Sinemurian of Dorset is sorely in need of taxonomic revision, as has recently been carried out for German dapediid material [28,37,41].

Further uncertainty surrounds the identity of the sister taxon of dapediids. Close relationships have variably been found with pholidophorids + leptolepids [29,34] and pachycormids [31], but most commonly with pycnodonts [27,30,32,42]. Pycnodonts are a clade of deep-bodied neopterygians with heterodont dentition [43], and are themselves of uncertain phylogenetic affinity. They have traditionally been affiliated with teleosts [30,32,44,45], but as noted by Poyato-Ariza [46], these results largely rely on verbal argumentation or small character-by-taxon matrices. As such, they lack the quantitative rigour necessary to evaluate placement of pycnodonts relative to other neopterygians. Most analyses of neopterygian interrelationships exclude pycnodonts [6,8,10,14,32,33,47,48], probably due to their highly specialized morphology. Furthermore, detailed analyses of pycnodont interrelationships [44,49–51] rarely include robust sampling of non-pycnodont neopterygians, sometimes using just a single, hypothetical outgroup. The most inclusive analysis to date recovers a stem neopterygian position for pycnodonts [46], although this analysis codes a single, composite taxon and does not include dapediids.

The equivocal phylogenetic signal of dapediids is in part due to the conflicting datasets used to resolve relationships. The phylogenetically informative endoskeleton [32,52,53], in particular the braincase, is described for only a small number of dapediids (*Dapedium punctatum*, *D. caelatum* [18]), and exhaustively in just a single taxon (*Dapedium* sp. [27,38,40,54,55]). While three-dimensional crania are known in a number of taxa (e.g. *Tetragonolepis semicincta* [17]; *Dapedium granulatum* [22], the majority of dapediids are preserved as laterally compressed fossils, with heavy dermal ornament obscuring most of the internal skeleton. In particular, no neurocrania are described from the Triassic, when the group first appeared.

**Figure 1.**
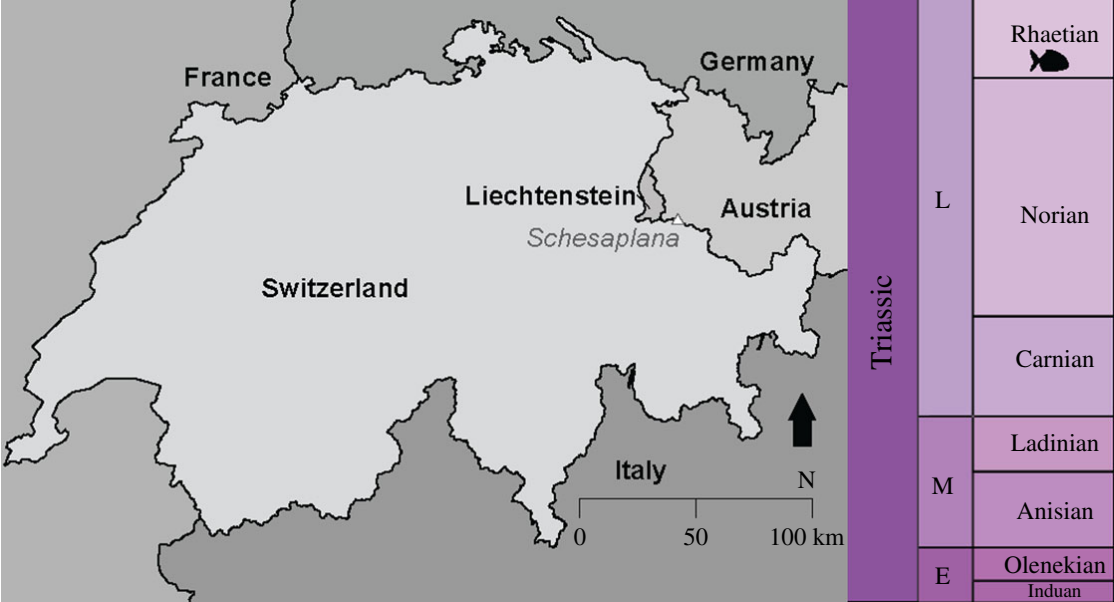
Map showing specimen locality (Schesaplana, white triangle) and putative age of deposit (Rhaetian).

Here we present a new, large (approx. 17 cm length) braincase from the Rhaetian (Late Triassic) of the Kössen Formation (Schesaplana, Grisons, Switzerland; figure 1). Marine reptiles and pterosaurs have previously been described from this locality [56–58]. Actinopterygian remains attributed to *Birgeria* have been described from a lateral extension of the formation into Austria [59], but no dapediids have previously been identified. This new specimen expands our knowledge of the taxonomic richness of the locality and increases the size range known for dapediids, with implications for the diversification of the group and the timing of their radiation. Furthermore, CT scanning of this well-preserved neurocranium presents an opportunity to revisit the phylogenetic affinities of the group.

## 2. Material and methods

### 2.1. CT scanning

PIMUZ (Paleontological Institute and Museum, University of Zurich, Switzerland) A/I 3026 was scanned at EMPA (Swiss Federal Laboratories for Materials Science and Technology, Dübendorf, Switzerland) using a 300 kV micro-focus source (Finetec FOMR300.0) and flat-panel detector (Perkin-Elmer XRD 1621 AN14 ES, 200 µm pixels) at 100 µm resolution. VGStudio Max V2.2.5 and Mimics v.19 were used for digital segmentation, and meshes were exported into and imaged in Blender (blender.org). Scan data and three-dimensional surface files (.PLYs) are available on MorphoMuseuM (https://doi.org/10.18563/journal.m3.44).

### 2.2. Phylogenetic analysis

Our dataset is based on Giles *et al.* [8], with 25 additional characters both novel and taken from the literature [10,14,33,36,45,46,49]. Eighteen taxa were added to the matrix in order to increase the sampling of dapediids and investigate the relationship of pycnodonts to other neopterygians. *Brachydegma caelatum* was removed pending a redescription of this taxon. This gives a total of 291 characters and 110 taxa. Full details are given in the electronic supplementary material. An equally weighted parsimony analysis was conducted using a heuristic search in PAUP* v. 4.0a158 [60] with the following settings: 1000 random addition sequences, five trees held at each step, maxtrees set to automatically increase, nchuck = 10 000, chuckscore = 1, tree bisection and reconstruction strategy enabled. Seven characters (c.89, c.91, c.151, c.171, c.241, c.247, c.251) were ordered following Giles *et al*. [8]. Taxonomic equivalence [61] was assessed using Claddis [62]. The outgroup was constrained using the topology [*Dicksonosteus* [*Entelognathus* [*Acanthodes*, *Cladodoides*, *Ozarcus*][ingroup]]]. An agreement subtree was calculated in PAUP. Bootstrap values were calculated in PAUP with the following settings: 1000 replicates of a heuristic search, tree branching and reconstruction strategy enabled, 25 replicates, five trees held at each step, rearrlimit = 50 000 000, limitperrep = yes, nchuck = 10 000, chuckscore = 1. An optimization tree, showing all ambiguous character changes, was generated in MacClade ([63]; electronic supplementary material, Data S1).

## 3. Results

### 3.1. Systematic palaeontology

Osteichthyes Huxley 1880Actinopterygii Cope 1887Holostei Müller 1844Dapediidae Lehman 1966*Scopulipiscis saxciput* gen. et sp. nov.

#### 3.1.1. Etymology

Generic name describes the marine deposits on the alpine cliff sides of Schesaplana *Scopuli*- from *scopulus* (Latin)—meaning a lookout place, cliff or crag, in or under the sea-, *piscis* (Latin) for fish. In the specific epithet, *sax*- (Latin) is for ‘rock’ and -*ciput* from *caput* (Latin) for head because the holotype specimen PIMUZ A/I 3026 is a well-ossified neurocranium.

#### 3.1.2. Holotype

**Figure 2.**
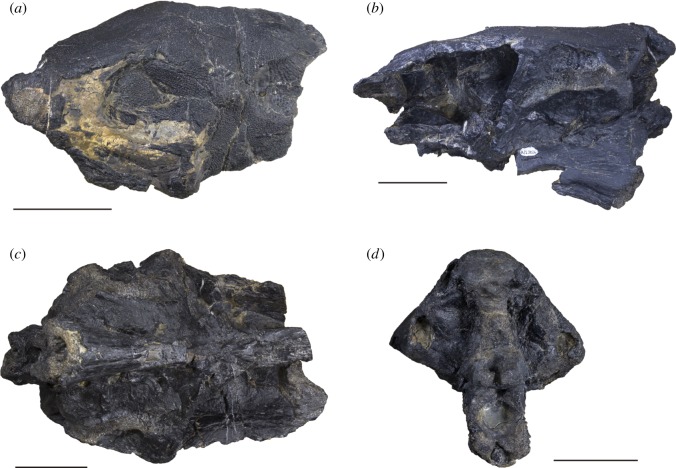
Photographs of the holotype of *Scopulipiscis saxciput* gen. et sp. nov. (PIMUZ A/I 3026). Specimen in dorsal (*a*), left lateral (*b*), ventral (*c*) and posterior (*d*) view. Scale bars are 50 mm.

PIMUZ A/I 3026 (figure 2), a neurocranium and associated dermal elements infilled by a fine carbonate matrix including small (approx. 2–8 mm) bivalves. Collected in 1976 by Dr Heinz Furrer, PIMUZ, the specimen was subsequently prepared mechanically.

#### 3.1.3. Locality and horizon

The specimen was collected as float from the Kössen Formation of Schesaplana Mountain, in Canton Grisons/Graubünden, Switzerland (figure 1), in the lowest part of the Alplihorn Member, Late Triassic [56], where the boundary between the Late Norian and Early Rhaetian is currently undefined (Heinz Furrer 2016, personal communication). Actinopterygians from the same locality include fragmentary elements and teeth from *Birgeria* [64] and isolated elements in the collections at PIMUZ assigned to *Sargodon* and *Lepidotes*. The Austrian portion of the Kössen Formation has produced other fish remains [59]. Other taxa from Schesaplana include marine reptiles [56,58] and pterosaurs [57].

#### 3.1.4. Diagnosis

Large neopterygian with a well-ossified neurocranium and the following combination of characters: spiracular canal extending through the postorbital process to open on the ventral surface of the braincase; elongate hyomandibular facet approximately one-fourth of neurocranial length; pronounced median occipital crest, large sinus between lateral cranial canals, parasphenoid keel extending dorsally between orbits.

### 3.2. Anatomical description

#### 3.2.1. General

PIMUZ A/I 3026 preserves a partial skull roof and a near-complete neurocranium (17 cm preserved length), missing the nasal capsules and anterior portion of the orbit. There is some distortion, with the right orbit partially crushed and the basiocciput sheared slightly to the right. The skull roofing bones have also collapsed in the region of the anterior dorsal fontanelle. The complete ossification of the braincase and partial obliteration of dermal sutures suggests the cranium belonged to an adult specimen. Sutures between braincase ossifications are not apparent; the neurocranium appears to be ossified as a single unit. Some areas of perichondral bone were weathered away prior to collection, exposing cancellous bone, particularly in the occipital region and along the parasphenoid.

#### 3.2.2. Skull roof

**Figure 3.**
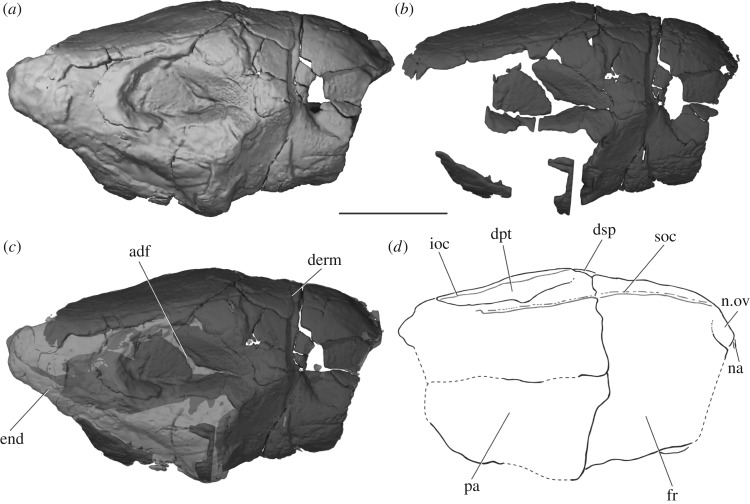
The skull roof of PIMUZ A/I 3026 in dorsal view. Rendering of entire specimen (*a*), dermal bone only (*b*) and position of preserved dermal bone (*c*). Interpretive drawing of skull roof (*d*). Dermal bone in dark grey, endochondral bone and matrix in light grey. Sensory canals in (*d*) in grey. Scale bar is 50 mm. adf, region of anterior dorsal fontanelle; derm, dermal bone; dpt, dermopterotic; dsp, dermosphenotic; end, endochondral bone; fr, frontal; ioc, infraorbital canal; na, nasal; n.ov, nasal overlap; pa, parietal; soc, supraorbital canal.

The dermal bones of the skull roof are heavily ornamented with pustular tubules, from 0.5 to 1 mm in diameter, radiating from ossification centres and increasing in size towards element margins (figures 2 and 3). The tubules are particularly large around the midline where the frontals and parietals meet. Such dense ornament means that sutures between bones are typically difficult to trace, although they can sometimes be followed in CT scans. The frontal is relatively short, accounting for a little under half the length of the skull roof (fr, figure 3*d*). It is widest anterior to the parietal contact, and tapers anteriorly. There is no pineal foramen, and the frontals appear to be fused at the midline. Only a small fragment of the left nasal is preserved, but an unornamented area at the anteriormost point of the left frontal represents the overlap area for this bone (n.ov, figure 3*d*). A series of pores marks the passage of the supraorbital sensory canal through the frontal and into the parietal (soc, figure 3*d*), and the path of the canal can be followed in CT sections. Very little of the midline suture is preserved between the elongate parietals (pa, figure 3*d*), which have partially collapsed into the anterior dorsal fontanelle (adf, figure 3*c*). The suture between the rectangular dermopterotic and parietal (dpt, figure 3*d*) is obscured by ornament externally, but can be traced in CT sections. However, it is unclear whether the dermopterotic contacts the frontal anteriorly. As with the supraorbital canal, the path of the otic sensory canal through the dermopterotic and into the dermosphenotic is indicated by a line of pores (ioc, figure 3*d*), with the canal visible in CT slices.

#### 3.2.3. Braincase

##### 3.2.3.1. Occipital region

**Figure 4.**
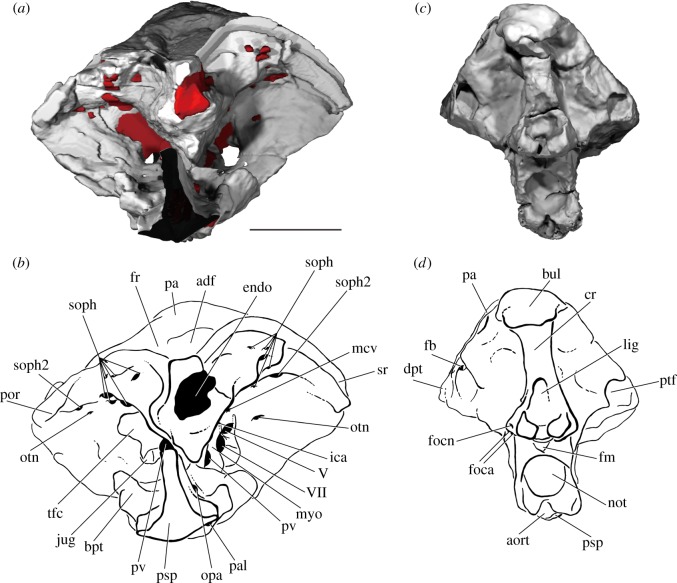
The braincase of PIMUZ A/I 3026. Rendering (*a*) and interpretive drawing (*b*) of the cranium in anterior view, with the dermal skull roof digitally removed anterior to the postorbital process and the anterior corpus of the parasphenoid cropped. Rendering (*c*) and interpretive drawing (*d*) of the cranium in posterior view. Braincase and dermal bones in light grey, endocast and canals in red. Scale bar is 50 mm. adf, region of anterior dorsal fontanelle; aort, dorsal aorta; bpt, basipterygoid process; bul, bulge; cr, crest; dpt, dermopterotic; endo, endocavity; fb, fossa bridgei; fm, foramen magnum; foca, occipital artery; focn, occipital nerve; fr, frontal; ica, internal carotid; jug, jugular vein; lig, ligament pit; mcv, middle cerebral vein; myo, myodome; not, notochord; opa, ophthalmic artery; otn, otic nerve; pa, parietal; pal, palatine artery; por, postorbital process; psp, parasphenoid; ptf, post-temporal fossa; pv, pituitary vein; soph, supraophthalmic artery; sr, skull roof; tfc, trigeminofacialis chamber; V, trigeminal nerve; VII, facial nerve.

**Figure 5.**
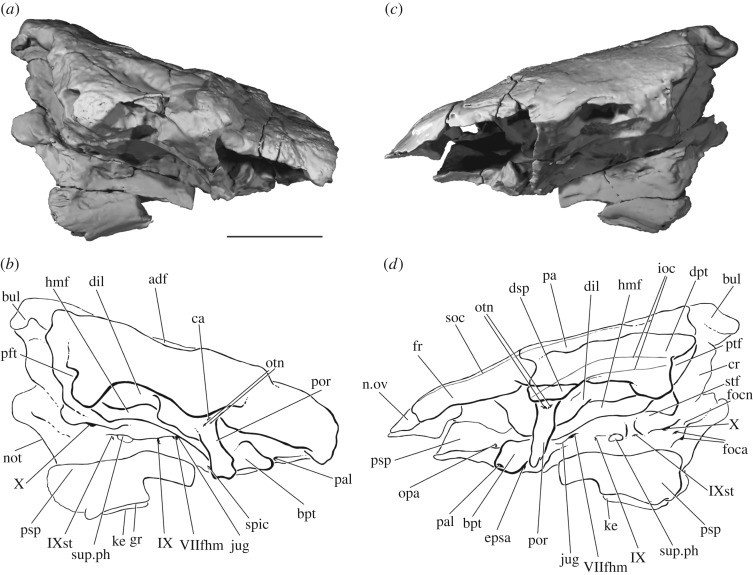
The braincase of PIMUZ A/I 3026. Rendering (*a*) and interpretive drawing (*b*) of cranium in right lateral view. Rendering (*c*) and interpretive drawing (*d*) of the cranium in left lateral view. Sensory canals in (*d*) in grey. Scale bar is 50 mm. adf, region of anterior dorsal fontanelle; bpt, basipterygoid process; bul, bulge; ca, canal; cr, crest; dil, dilatator facet; dpt, dermopterotic; dsp, dermosphenotic; epsa, efferent pseudobranchial; foca, occipital artery; focn, occipital nerve; fr, frontal; gr, groove; hmf, hyomandibular facet; ioc, infraorbital canal; jug, jugular vein; ke, keel; n.ov, nasal overlap; not, notochord; opa, ophthalmic artery; otn, otic nerve; pa, parietal; pal, palatine artery; por, postorbital process; psp, parasphenoid; ptf, post-temporal fossa; soc, supraorbital canal; spic, spiracular canal; stf, supratemporal fossa; sup.ph, suprapharyngobranchial articulation; VIIfhm, hyomandibular branch of the facial nerve; IX, glossopharyngeal nerve; IXst, supratemporal branch of the glossopharyngeal nerve; X, vagus nerve.

**Figure 6.**
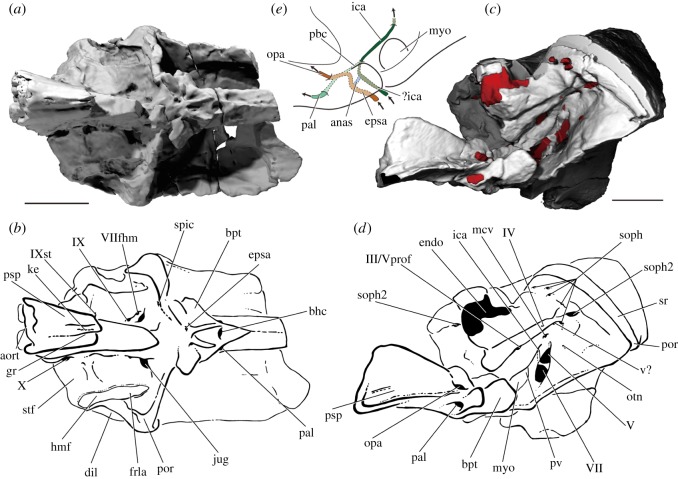
The braincase of PIMUZ A/I 3026. Rendering (*a*) and interpretive drawing (*b*) of the cranium in ventral view. Rendering (*c*) and interpretive drawing (*d*) of the cranium in left anterolateral view, with the dermal skull roof digitally removed anterior to the postorbital process. (*e*) Schematic drawing of the circulatory system in the basisphenoid and the parasphenoid in anterolateral view. The position of the entrance of the internal carotid in (*e*) is uncertain due to breakage. Braincase and dermal bones in light grey, endocast and canals in red. Scale bar is 50 mm. anas, anastomosis; aort, dorsal aorta; bhc, buccohypophyseal canal; bpt, basipterygoid process; dil, dilatator facet; endo, endocavity; epsa, efferent pseudobranchial; frla, recurrent lateralis branches of the facial nerve; gr, groove; hmf, hyomandibular facet; ica, internal carotid; jug, jugular vein; ke, keel; pal, palatine artery; mcv, middle cerebral vein; myo, myodome; opa, ophthalmic artery; otn, otic nerve; pbc, parabasal canal; por, postorbital process; psp, parasphenoid; pv, pituitary vein; soph, supraophthalmic artery; spic, spiracular canal; sr, skull roof; stf, supratemporal fossa; v?, unknown vein; III/Vprof, oculomotor nerve and profundus branch of the trigeminal nerve; IV, trochlear nerve; V, trigeminal nerve; VII, facial nerve; VIIfhm, hyomandibular branch of the facial nerve; IX, glossopharyngeal nerve; IXst, supratemporal branch of the glossopharyngeal nerve; X, vagus nerve.

**Figure 7.**
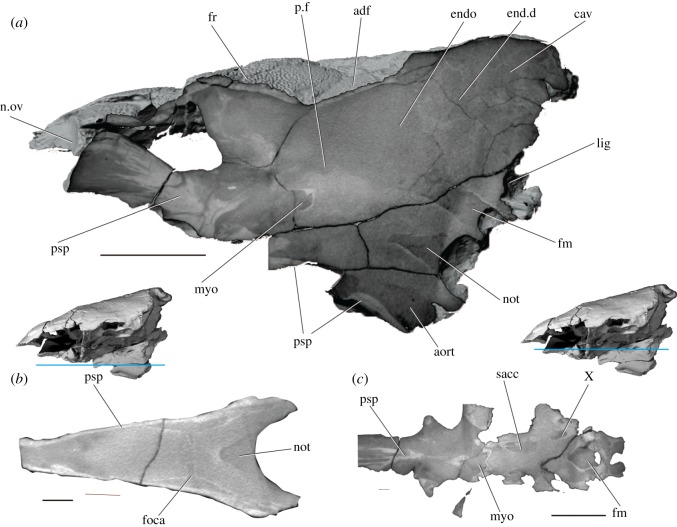
CT slices through the braincase of PIMUZ A/I 3026. (*a*) Sagittal section through the midline. Transverse planes through the basioccipital (*b*) and floor of the orbit and basisphenoid (*c*). Scale bars are 50 mm for (*a*,*c*) and 20 mm for (*b*). adf, region of anterior dorsal fontanelle; aort, dorsal aorta; cav, cavity; endo, endocavity; end.d, endolymphatic duct; foca, occipital artery; fr, frontal; fm, foramen magnum; lig, ligament pit; myo, myodome; n.ov, nasal overlap; not, notochord; p.f, pituitary fossa; psp, parasphenoid; sacc, sacculus; X, vagus nerve.

**Figure 8.**
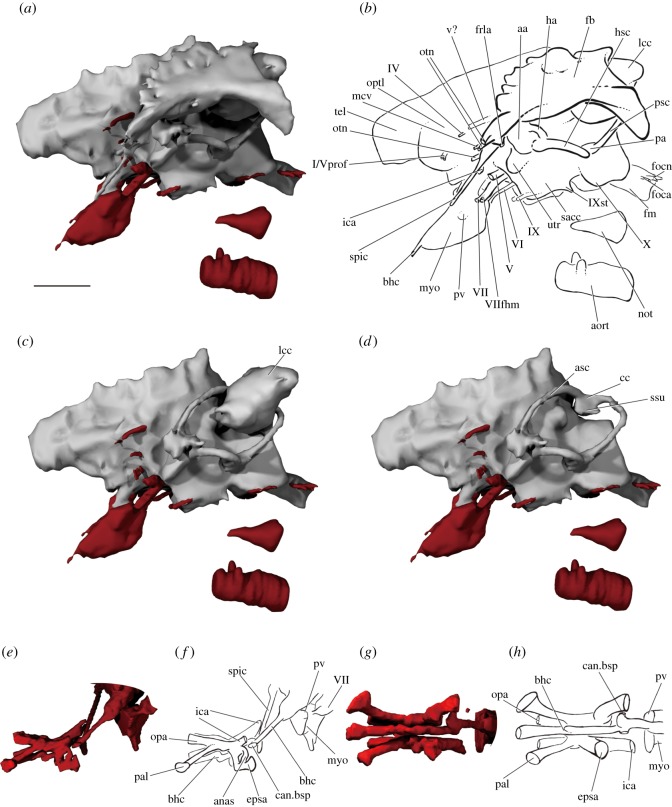
The endocast and cranial circulation of PIMUZ A/I 3026. Rendering (*a*) and interpretive drawing (*b*) of the endocast, fossa bridgei and lateral cranial canal in left lateral view, and with the fossa bridgei (*c*) and lateral cranial canal (*d*) digitally removed. Rendering (*e*,*g*) and interpretive drawing (*f*,*h*) of the basisphenoid and parasphenoid circulatory system in left lateral (*e*,*f*) and ventral (*g*,*h*) view. Endocast in grey, canals in red. Scale bar is 50 mm. aa, anterior ampulla; anas, anastomosing canal; aort, dorsal aorta; asc, anterior semicircular canal; bhc, buccohypophyseal canal; can.bsp, unidentified canal in basisphenoid; cc, crus commune; epsa, efferent pseudobranchial; fb, fossa bridgei; foca, occipital artery; focn, occipital nerve; fm, foramen magnum; frla, recurrent lateralis branches of the facial nerve; ha, horizontal ampulla; hsc, horizontal semicircular canal; ica, internal carotid; lcc, lateral cranial canal; mcv, middle cerebral vein; myo, myodome; not, notochord; opa, ophthalmic artery; optl, optic lobe; otn, otic nerve; pa, posterior ampulla; pal, palatine artery; psc, posterior semicircular canal; pv, pituitary vein; sacc, sacculus; spic, spiracular canal; ssu, sinus superior; tel, telencephalon; utr, utriculus; v?, unknown vein; III/Vprof oculomotor nerve and profundus branch of the trigeminal nerve; IV, trochlear nerve; V, trigeminal nerve; VI, abducens nerve; VII, facial nerve; VIIfhm, hyomandibular branch of the facial nerve; IX, glossopharyngeal nerve; IXst, supratemporal branch of the glossopharyngeal nerve; X, vagus nerve.

The occipital region is the deepest part of the braincase, with the basiocciput accounting for half the vertical height of the specimen (figures 4*c*,*d* and 5). It is narrower than either the otic or orbitotemporal regions (figure 6*a,b*). A ‘bulge’ on the midline projects dorsally above the skull roof (bul, figures 4*d* and 5*b*,*d*) and houses a large cavity within the perichondrium of the braincase (cav, figure 7*a*). The posterior face of the occiput is developed into a broad median occipital crest, punctuated by a large ligamentous pit (lig, figures 4*d* and 7*a*). A process lateral to this pit is pierced by small anastomosing branches for the occipital nerve (focn, figures 4*d* and 5*d*). Medial to the posterodorsal angle of the braincase is a deep post-temporal fossa (ptf, figures 4*d* and 5*b*,*d*), which is continuous anteriorly with the fossa bridgei (fb, figure 4*d*). The foramen magnum is small and triangular, and both its floor and roof are well-ossified (fm, figures 4*d* and 7*a*). Two foramina for the occipital arteries open onto the lateral face of the braincase just above the parasphenoid (foca, figures 4*d* and 5*d*), with the smaller opening for the occipital nerve dorsal to these (focn, figures 4*d* and 5*d*). The notochordal facet is large and rounded (not, figure 4*d*), and leads into a narrow notochordal canal that pinches out approximately halfway along the length of the basiocciput (not, figures 7*a*,*b* and 8*b*). Ventral to the opening for the notochord is the circular aortic canal, which is enclosed ventrally by the parasphenoid (aort, figures 4*d*, 6*b* and 7*a*). A dorsally directed canal leaves the dorsal margin of the aorta to open on the lateral wall of the braincase above the parasphenoid. There is no indication of either a posterior dorsal fontanelle or otoccipital fissure.

##### 3.2.3.2. Otic region

**Figure 9.**
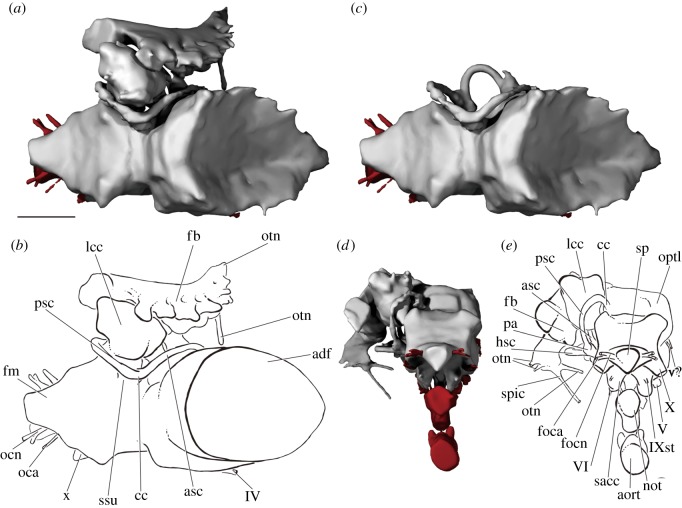
The endocast of PIMUZ A/I 3026. Rendering (*a*) and interpretive drawing (*b*) of the endocast, fossa bridgei and lateral cranial canal in dorsal view. Rendering (*c*) and interpretive drawing (*d*) of the endocast, fossa bridgei and lateral cranial canal in posterior view. Endocast in grey, canals in red. Scale bar is 50 mm. adf, anterior dorsal fontanelle; aort, dorsal aorta; asc, anterior semicircular canal; cc, crus commune; fb, fossa bridgei; fm, foramen magnum; hsc, horizontal semicircular canal; lcc, lateral cranial canal; not, notochord; oca, occipital artery; ocn, occipital nerve; optl, optic lobe; otn, otic nerve; pa, posterior ampulla; psc, posterior semicircular canal; sp, spinal nerve cord; spic, spiracular canal; ssu, sinus superior; v?, unknown vein; IV, trochlear nerve; V, trigeminal nerve; VI, abducens nerve; IXst, supratemporal branch of glossopharyngeal nerve; X, vagus nerve.

The otic region is the widest part of the braincase (figure 6*a*,*b*). Much of its dorsal surface is unossified to accommodate the large anterior dorsal fontanelle, which extends from the posterior margin of the hyoid facet to above the orbits (adf, figures 3*c*, 4*b* and 9*b*). Lateral to the fontanelle is an excavation for the fossa bridgei (fb, figures 8*b* and 9), which extends posteriorly to the post-temporal fossa and anteriorly to the postorbital process. The lateral margin of the fossa bridgei is strongly concave, wrapping around the hyomandibular facet. The medial margin is convex, with multiple small projections giving an irregular edge. A total of five canals connect with the fossa bridgei. Two canals issue from its anteromedial corner. The more ventral, for the spiracular canal, enters the top of the postorbital process and travels along its entire length, opening just posterior to the lateral commissure on the ventral surface of the braincase (spic, figures 5*b*, 6*b* and 9*e*). On the left side, the lateral commissure is broken, and consequently this canal appears to open midway along its length. The more dorsal of the two anteromedial canals opens into the posterolateral corner of the orbit, just below the orbital roof, and transmitted the otic nerve (otn, figures 4*a*, 6*b*,*d* and 8*b*). An additional canal leaves more posteriorly from the medial margin and opens into the roof of the hyomandibular facet and may have transmitted the recurrent lateralis branch of the facial nerve (frla, figures 6*b* and 8*b*). Two narrow canals exit the anterolateral corner to open on the side of braincase just below the skull roof, and probably housed branches of the otic nerve (otn, figures 5*b*,*d*, 8*b* and 9*b*,*e*). Dorsal to the fossa bridgei is a separate small cavity, which also lies within the perichondrium of the braincase. Owing to specimen preservation, the chamber is only fully observable on the right side. The chamber has a medial connection to the orbit, opening into the dorsolateral corner of the posterior wall (soph2, figure 4*b*). An additional small canal leaves the lateral margin of the chamber to open onto the lateral surface of the braincase dorsal to the otic nerve canals (ca, figure 5*b*).

The articular facet for the hyomandibular is elongate and horizontally oriented (hmf, figures 5*b*,*d* and 6*b*), and a deep dilatator fossa lies dorsal to the hyoid articulation (dil, figures 5*b*,*d* and 6*b*). Ventral to the facet, the lateral wall of the braincase narrows towards the midline, and this face is pierced by a number of canals. The most posterior of these is the lenticular opening for the vagus nerve (X, figures 5*b*,*d* and 6*b*), positioned ventral to the posterior margin of the hyoid facet. A shallow depression between the vagus nerve and hyoid facet is the supratemporal fossa (stf, figures 5*d* and 6*b*), delimited anteriorly by a vertical strut. The glossopharyngeal nerve (probably its supratemporal branch) exits the braincase via a small canal at the base of this strut (IXst, figures 5*b*,*d* and 6*b*), adjacent to an elliptical pit. This pit appears to be at least partly the result of mechanical preparation, as it punches through the perichondrium to reach the saccular chamber on one side, but comparison of both sides shows that the perichondral lining of the endocavity is complete, and there is no vestibular fontanelle. The original morphology of the pit is unclear, but it may represent an articular facet for the first suprapharyngobranchial (sup.ph, figure 5). A shallow groove for the jugular marks the lateral face of the braincase anterior to this facet, and can be traced to a foramen in the lateral commissure (jug, figures 5*b*,*d* and 6*b*). A large opening on the ventral margin of the jugular groove marks the exit of a stout canal, which originates on the lateral face of the sacculus near its anterior margin. This canal may be the main trunk of the glossopharyngeal nerve (IX, figures 5*b*,*d* and 6*b*). More anteriorly, a large opening for the hyomandibular branch of the facial nerve (VIIfhm, figures 5*b*,*d* and 6*b*) pierces the roof of the jugular groove. The jugular vein and main trunk of the facial nerve continue through the postorbital process into the orbit.

##### 3.2.3.3. Orbitotemporal region

The posterior margin of the orbitotemporal region is formed by the lateral commissure and postorbital process (por, figures 4*b*, 5*b*,*d* and 6*b*,*d*). The lateral commissure is reduced to a thin splint laterally, and the extent to which it is covered by the ascending process of the parasphenoid is difficult to discern. The orbital walls are pierced by a complex anastomosing series of canals, some of which are difficult to identify.

These canals can roughly be separated into four groups: on the roof and posterolateral corner of the orbit; on the posteromedial corner of the orbit; on the medial wall of the orbit; and midway up the posterior orbital wall. Of the canals that open onto the roof of the orbit, around four to six transmit the superficial ophthalmic branches of the facial and trigeminal nerves dorsally through the braincase to contact the visceral surface of the skull roof (soph, figures 4*b* and 6*d*). The two canals in the posterolateral corner of the orbit have different points of origin: the more ventral, for the otic nerve (otn, figures 4*b*, 6*d*, 8*b* and 9*b*,*e*), connects to the fossa bridgei; and the more dorsal, which may also carry branches of the superficial ophthalmic (soph2, figures 4*b* and 6*d*), to the chamber that lies above the fossa bridgei. Of the two canals in the posteromedial corner of the orbit, the more anterior and dorsal carries the trochlear nerve from the optic lobes (IV, figures 6*d*, 8*b* and 9*b*). The more posterior originates from the dorsal part of the midbrain, above and some way posterior to the trochlear nerve, but its identity is unclear (v?, figures 6*d*, 8*b* and 9*e*). Two canals open on the medial wall of the orbit via a single foramen. These canals have a single root on the forebrain on the right of the specimen and a double root on the left, and transmitted the oculomotor and profundus nerves into the orbit (III and Vprof, figures 6*d* and 8*b*). The final set of canals, approximately midway up the posterior orbital wall, is centred about the trigeminofacialis chamber (tfc, figure 4*b*), which is continuous posteroventrally with the jugular canal. Two canals exit from the same point on the anterior face of the utriculus before separating into separate canals. These canals enter the orbit separately, but both are continuous within the trigeminofacialis chamber. The more dorsal transmits the trigeminal nerve (V, figures 4*b*, 6*d*, 8*b* and 9*e*) and the more ventral the facial nerve (VII, figures 4*b*, 6*d* and 8*b*,*f*). Two grooves extend from the dorsal margin of the chamber. The groove on the posterior margin of the orbit is short, with no canal at its dorsal limit. It terminates some way ventral and medial to any of the canals that connect to the skull roof, but may have transmitted the superficial ophthalmic nerves, which entered the orbit via the underlying chamber. The groove on the medial orbital wall is deeper, and a canal at its dorsal margin connects to the cranial cavity via two closely positioned openings. This probably transmitted the internal carotid artery (ica, figures 4*b*, 6*d* and 8*d*). A ventral connection links the trigeminofacialis chamber and the median posterior myodome (myo figures 4*b*, 6*d* and 8*b*,*f*,*h*). The pituitary vein enters the orbit ventral to the trigeminofacialis chamber (pv, figures 4*b*, 6*d* and 8*b*,*f*,*h*). There is no obvious opening for the palatine branch of the facial nerve into the orbit.

The braincase is broken midway along the orbit, and the skull roof has partially collapsed. A single opening on the midline represents where the braincase is broken through the endocranial cavity of the forebrain (endo, figures 4*b* and 6*d*); neither the exit of the optic (II) nor olfactory (I) nerve is preserved. The basisphenoid is well ossified, contributing to the medial walls and floor of the orbit, although the interorbital septum is formed entirely by the parasphenoid (psp, figures 4*b*, 5*d*, 6*d* and 7*a*). The basipterygoid process, which has both a dermal and endoskeletal component, is robust. The internal carotid opens into the posterior margin of the basisphenoid via a foramen anterior to the posterior myodome. The subocular shelf is narrow and formed entirely by the parasphenoid.

#### 3.2.4. Parasphenoid

The parasphenoid extends the full length of the preserved braincase, although a fragment is missing in the middle where the specimen is broken between the midpoint of the basiocciput and the postorbital process. The posterior margin of the parasphenoid is weathered, exposing the basioccipital, but a midline aortic notch appears to have been absent (figures 4 and 6). Although housed entirely within the basiocciput posteriorly, most of the floor of the aortic canal is formed by the parasphenoid (aort, figure 7*a*). As the parasphenoid is broken midway along the basiocciput, the nature of the bifurcation into the lateral aortae is unknown. A narrow median keel on the ventral surface of the parasphenoid (ke, figures 5*b*,*d* and 6*b*) is flanked by paired shallow grooves (gr, figures 5*b* and 6*b*); the depth of the left groove has been exaggerated during specimen preparation. As the dorsal and lateral aortae are enclosed within the braincase, the purpose of these grooves is unclear. The parasphenoid extends up the lateral walls of the braincase to the level of the foramen magnum, effectively cloaking the basiocciput. The ascending processes of the parasphenoid are narrow and strut-like, although it is difficult to assess how far they extend dorsally up the postorbital process. The basipterygoid processes are robust, projecting dorsally as well as laterally (bpt, figures 4*b*, 5*b*,*d* and 6*b*,*d*), and the ophthalmic artery pierces the orbital floor posteromedial to the process (opa, figures 4*b*, 5*d* and 6*d*,*e*). An opening at the anterior limit of the basipterygoid process marks the exit of the palatine nerve and artery from the basisphenoid (pal, figures 4*b*, 5*b*,*d* and 6*b*,*d*,*e*). A V-shaped ridge is present on the ventral surface of the parasphenoid, and the efferent pseudobranchial artery exits posterior to where the two arms of the ridge converge on the midline (epsa, figures 5*d* and 6*b*,*e*). Anteriorly, the buccohypophyseal canal exits the parapshenoid on the midline (bhc, figure 6*b*). The internal carotid foramen is not preserved, and presumably entered the parasphenoid through the missing section. A broad parasphenoid keel extends dorsally between the orbits, forming the interorbital septum (psp, figures 4*b*, 5*d*, 6*d* and 7*a*). No teeth are present on the parasphenoid.

#### 3.2.5. Endocast

The regions of the endocranial cavity corresponding to the midbrain, hindbrain and bony labyrinth are almost completely preserved, although only a short portion corresponding to the narrow forebrain is present in the specimen (tel, figure 8). As the internal walls of the braincase are sometimes poorly mineralized (figure 7*a*), contrast between the braincase and infilling sediment is low, with the exact boundaries of some regions of the endocranial cavity difficult to discern.

##### 3.2.5.1. Midbrain

The area of the optic lobes (midbrain) is large, representing the widest part of the endocast (optl, figure 8*b*). Much of the dorsal surface is unfinished, indicating the position of the extensive anterior dorsal fontanelle (adf, figure 8*b*). Two closely associated canals leave from the anterolateral face of the midbrain, entering the orbit via a single (on the right of the specimen) or double (on the left) opening. These probably transmitted the oculomotor and profundus nerves (III/Vprof, figure 8*b*). The trochlear nerve leaves the cranial cavity posteriorly and dorsally (IV, figures 8*b* and 9*b*).

##### 3.2.5.2. Hindbrain

There is no clear division between the midbrain and hindbrain, although the portion of the hindbrain positioned behind the crus commune sits far ventral relative to the rest of the endocavity, perhaps indicating that the cerebellum projected into the fourth ventricle. Cerebellar auricles cannot be identified. Several canals exit the cranial cavity in the anterior part of the hindbrain. Dorsally, a stout canal transmits an unidentified vessel into the dorsal part of the orbit (v?, figure 8*b*). The middle cerebral vein exits the cranial cavity some way ventral to this (mcv, figure 8*b*), and the internal carotid enters more ventrally still (ica, figure 8*b*). The trigeminal and facial nerves exit from the angle between the midbrain and utricular recess (V and VII, figure 8*b*). Posterior to the labyrinth, the vagus nerve leaves the ventral margin of the hindbrain through a large, anteroposteriorly elongate canal (X, figure 8*b*). The region corresponding to the spinal cord is by far the narrowest portion of the endocast, and from its lateral margins issue a dorsal, bifurcating canal for the occipital nerves (focn, figures 8*b* and 9*b*,*e*), and a ventral, single canal for the occipital artery (foca, figures 8*b* and 9*b*,*e*).

##### 3.2.5.3. Labyrinth

The walls of the bony labyrinth are fairly well ossified, except in the region of the sinus superior and anterior ampulla. The three semicircular canals are narrow, and all are fully enclosed in bone (asc, hsc, psc, figures 8*b* and 9*b*,*e*). Both the anterior and posterior canals are anteroposteriorly short, and the horizontal canal is almost isoclinal in its curvature (figure 9*c*). The orientation of the anterior ampulla is unclear (aa, figure 8*b*). The utriculus is dorsoventrally tall, and there is a slight suggestion of division into dorsal and ventral components (utr, figure 8*b*). A small saccular notch is present (sac, figure 8*b*). Although incompletely ossified, the sinus superior is long (ssu, figure 8*d*), and the crus commune projects above the roof of the hindbrain and slightly towards the midline (cc, figures 8*d* and 9*b*). The lateral cranial canal is very well developed (lcc, figures 8*b* and 9*b*,*e*). As well as anterior and posterior connections to the cranial cavity, it has a dorsal extension that almost reaches the underside of the skull roof. At least a single canal, probably for the endolymphatic ducts (end.d, figure 7*a*), connects the cranial cavity to the median sinus positioned between the lateral cranial canals.

##### 3.2.5.4. Circulatory system

The buccohypophyseal canal is elongate, leaving the anteriormost point of the posterior myodome and continuing anteroventrally (bhc, figure 8). A narrow canal branches from its lateral margin level with the exit of the internal carotids into the orbit (can.bsp: unidentified canal in the region of the basisphenoid, figure 8*f*,*h*). Further anteriorly, the buccohypophyseal canal bends dorsally in an ‘s’ shape, before opening onto the ventral surface of the parasphenoid. The path of the internal carotid artery is incompletely known, largely due to the loss of part of the parasphenoid. It presumably entered the parasphenoid posterior to the basipterygoid process (?ica, figure 6*e*) and continued via an anterodorsally directed canal. This canal opens onto the orbital floor posteromedial to the basipterygoid processes (ica, figures 6*e* and 8*f*,*h*), and enters the cranial cavity through a foramen in a dorsally directed groove medial to the trigeminofacialis chamber (ica, figure 6*d*,*e*). The efferent pseudobranchial pierces the parasphenoid beneath the basipterygoid process (epsa, figures 5*d*, 6*d* and 8*f*,*h*) and is transmitted through a vertical canal, exiting onto the floor of the orbital as the ophthalmic artery (opa, figures 4*b*, 5*b*,*d*, 6*d*,*e* and 8*f*,*h*). The internal carotid and efferent pseudobranchial communicate via a short anastomosing canal (anas, figures 6*e* and 8*f*), and are also linked via the parabasal canal (pbc, figure 6*e*), which seems to have housed the palatine nerve and artery. The path of the palatine into the orbit is not known, but it appears to have entered the parabasal canal through the same opening as the internal carotid, and opens anteriorly on the lateral margin of the parasphenoid (pal, figures 4*b*, 5*b*,*d*, 6*b*,*d*,*e* and 8*f*,*h*). The internal carotids and efferent pseudobranchials do not anastomose with their antimeres, but a narrow transverse canal connects the two ophthalmic arteries.

#### 3.2.6. Body size

The neurocranium of *Dapedium* is inclined at approximately 50° from the horizontal in articulated specimens, and body size typically ranges from around 15 to 35 cm [18]. Skull length accounts for approximately 18% of body length in *Dapedium*, but accounts for a much smaller proportion (approx. 10%) in the largest known dapediids. Body length of a large *Sargondon tomicus* is estimated about 1 m [12], with absolute head length of a medium-sized specimen approximately 6 cm and of the large specimen approximately 10 cm [65].

The length of the preserved portion of PIMUZ A/I 3026 is 17 cm. Based on the proportions of the skull of *Dapedium*, a conservative estimate for the length of the unbroken skull is 20 cm. Comparison to the largest known dapediid suggests a body length in the region of 2 m.

## 4. Discussion

***Scopulipiscis saxciput*** gen. et sp. nov. as a dapediid.

Although represented only by an isolated neurocranium, this specimen bears conspicuous similarities to dapediids, in particular the braincase described for *Dapedium* sp. [27,55]: short aortic canal, parasphenoid ‘wings’ cloaking the basiocciput, median occipital crest punctuated by a large ligamentous pit, parasphenoid extending dorsally between the orbits, fossa bridgei continuous posteriorly with the post-temporal fossa, median sinus between the lateral cranial canals, canals for endolymphatic ducts connecting the cranial cavity to the median sinus and absence of differentiated braincase ossifications. Similar likenesses are found with the endocast of *Dapedium* sp. [40], including a large lateral cranial canal extending dorsal to the endocranial roof, narrow semicircular canals and a lenticular utriculus. The majority of actinopterygian material from Schesaplana has been identified as either *Birgeria* or *Lepidotes* [66] although unpublished elements in the collections at PIMUZ have been referred to the giant dapediid taxon *Sargodon* (A. Latimer 2016, personal observation), known from the Late Triassic (Norian) of Italy [12]. The limited anatomical overlap between *Sargodon* and the braincase described here makes comparisons difficult: *Sargodon* is known from articulated but laterally compressed fossils, with no part of the braincase, and only small portions of the skull roof, described. Differences are evident in some of the few comparable regions, for example the parietals and dermopterotic of *Sargodon* are quadrate, and those of the specimen described here are markedly rectangular. Furthermore, the cranium of even the largest *Sargodon* specimen known is only around 10 cm in length. By contrast, the braincase described here is approximately 17 cm, and is incomplete anterior to the orbit, suggesting a total cranial length of at least 20 cm. Consequently, we feel justified in erecting a new genus and species for this specimen: *Scopulipiscis saxciput*. It is possible that undescribed cranial elements at PIMUZ belong to *Scopulipiscis saxciput* rather than *Sargodon*, as their identification was most probably based on size.

**Figure 10.**
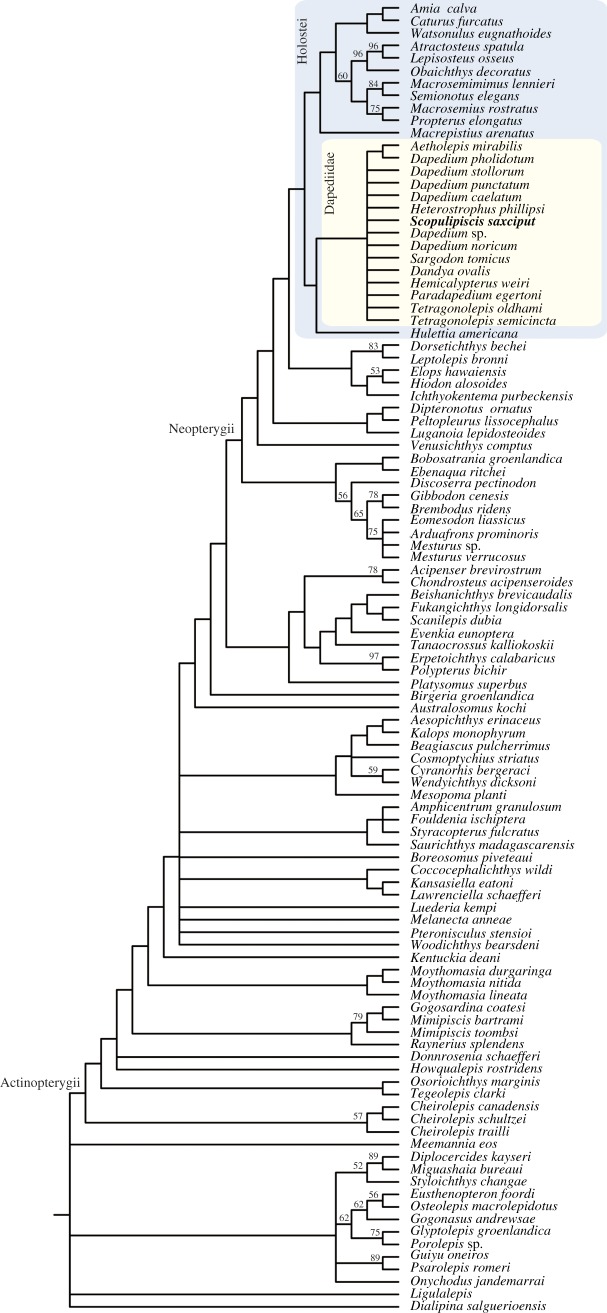
Phylogenetic placement of *Scopulipiscis saxciput* gen. et sp. nov. Strict consensus of the 20 000 shortest trees (1522 steps) for 110 taxa and 291 equally weighted characters. Numbers above nodes represent bootstrap support. Outgroup taxa (*Dicksonosteus*, *Entelognathus*, *Acanthodes*, *Cladodoides*, *Ozarcus*) excluded for purposes of space.

In the strict consensus tree of our phylogenetic analysis (figure 10), *Scopulipiscis saxciput* is resolved in a polytomy with all included *Dapedium* species, *Heterostrophus phillipsi*, *Sargodon tomicus*, *Dandya ovalis*, *Hemicalypterus weiri*, *Paradapedium egertoni*, *Tetragonolepis oldhami* and *Tetragonolepis semicincta*. This clade is supported by seven homoplastic characters: c.58, one or two suborbitals; c.156, braincase ossifications not differentiated; c.247, complete series of dorsal ridge scales; c.258, caudal fin not forked; c.269, suborbitals extend ventral to orbit; c.275, hem-like median fins; c.276, long-based anal fin. The monophyly of *Dapedium* is not upheld, and the genus is recovered as paraphyletic in both the Adams consensus tree and agreement subtree (electronic supplementary material, figure S1). Additional characters found in dapediid taxa to the exclusion of immediate outgroups, but which cannot be optimized due to uncertainty over the primitive condition in the clade, include: c.270, coronoids contribute to lateral dentition field; c.273, dorsal extension of parasphenoid between orbits; c.274, ligament pit on posterior face of braincase; c.278, ossified centra absent.

The failure to resolve *Dapedium* as monophyletic is hardly surprising, given that placement of material in this genus traditionally largely relied on the presence of a deep-bodied morphology, and differentiation of species on characters that could covary in individual specimens [12]. Doubts over the monophyly of the genus have been raised before [28], and it is possible that detailed investigation of Sinemurian material from Dorset may find further objections. Only four dapediid taxa are excluded in the agreement subtree (electronic supplementary material, figure S1a), suggesting that only some of the uncertainty stems from conflicting endoskeletal and dermal character sets. The monophyly of dapediids, in contrast, seems well supported, albeit with a different set of characters to those identified in previous phylogenetic analyses [14,37].

### 4.1. Placement of dapediids

Dapediids (along with *Hulettia*) form a clade of stem holosteans, sharing with other holosteans the following five homoplastic characters: c.89, two infradentaries; c.112, anterodorsal process of suboperculum; c.135, anterodorsal myodome single; c.173, parasphenoid with multifid anterior margin; c.178, aortic notch in parasphenoid absent. While dapediids have been recovered as total group holosteans before, they are more typically resolved as the sister group to ginglymodians [36,37]. A stem holostean position was recovered by López-Arbarello & Sferco [10] on the basis of two homoplastic characters (four or more supraorbitals and a presupracleithrum), neither of which are optimized as supporting this relationship in the current analysis. A position deep on the holostean stem goes some way to explaining the peculiar combination of apparent holostean and teleost features that have influenced past hypotheses of phylogenetic relationships.

The position of *Hulettia* as sister taxon to dapediids, with the support of three homoplastic characters (c.247, basal fulcra on dorsal fin; c.249, scutes anterior to anal fin; c.284, preoperculum shorter than operculum), is unexpected. We note that *Hulettia* shows large numbers of character reversals (for the full list of character optimizations see electronic supplementary material, Supplementary Data), suggesting that this position may not be robust. However, *Hulettia* has previously been associated with *Dapedium* [32]. *Macrepistius* is resolved as sister to all other holosteans, supported by one unambiguous (c.10, olfactory nerve pierces premaxilla) and five homoplastic characters (c.14, single median dermal bone capping snout; c.50, tube-like canal bearing anterior arm of antorbital; c.61, circumorbital ring of supraorbitals and infraorbitals closes the orbit; v.107, paired vomer; c.161, sphenotic with small dermal component). The holostean crown is united by just three homoplastic characters (c.175, parasphenoid teeth small; c.176, parasphenoid not pierced by internal carotid; c.272, parasphenoid wings around basiocciput absent), indicating that features previously identified as uniting the living radiations may in fact be more widely distributed across the total group.

### 4.2. Placement of pycnodonts

Pycnodonts are recovered as stem neopterygians in our analysis. The monophyly of pycnodonts is robustly supported by one unambiguous (c.282, prearticular symphysis) and thirteen homoplastic characters. *Discoserra* is recovered as the immediate outgroup of pycnodonts, with a clade comprising *Bobasatrania* + *Ebenequa* falling as the sister taxon. While this broader clade is supported by five homoplastic characters, we do not regard it as robust: at least some of the characters optimized as supporting this group (e.g. c.276, long-based anal fin) probably reflect anatomical similarities related to a deep-bodied morphology rather than a close phylogenetic relationship. Furthermore, *Platysomus* is resolved as sister taxon to an unlikely clade comprising cladistians and chondrosteans. These incongruent placements highlight that further work is needed to resolve the relationships of other radiations of deep-bodied Palaeozoic fishes.

A limited understanding of the internal skeleton of pycnodonts—and those taxa recovered as closely related—may also be a contributing factor. Despite an extensive fossil record stretching from the Late Triassic to the Eocene, few pycnodont neurocrania are described: the most comprehensive description of an early occurring pycnodont braincase is based on a specimen that now appears to be lost (*Mesturus* sp. [67]). Derived pycnodont neurocrania are known from well-preserved Cretaceous specimens of *Neoproscinetes penlavi* and *Iemanja palma* from Brazil, but are poorly ossified [50,68]. The earliest described pycnodonts are named from articulated but two-dimensional remains from the Norian of Italy [69], with isolated dental elements found in Norian and Rhaetian deposits of Belgium and Luxembourg [70]. As such, we consider our results relating to pycnodonts as preliminary: only a small proportion of the vast diversity of pycnodonts are included, and only one for which the braincase is known in detail. In particular, detailed description of primitive pycnodonts (for example, material from the Early Norian of Italy mentioned as possessing lateral gulars [71]), as well as three-dimensional neurocranial material, may help clarify the position of the group.

### 4.3. Dapediid diversity

Ray-finned fishes with an adaptation to durophagy were well represented in the Triassic [21,72], although dapediids are thought to have made up a fairly small proportion of this diversity. Although many durophages were wiped out during the End-Triassic extinction, dapediids survived and went on to radiate in the Jurassic, presumably filling vacated ecospace [73]. The discovery of a new, exceptionally large [74] dapediid in the Late Triassic indicates that dapediids may have been more diverse before the End-Triassic extinction than previously thought. Although dental elements are not preserved for *Scopulipiscis saxciput*, similarities in braincase anatomy to *Dapedium* seem likely to perform a functional role in strengthening the skull during durophagy, including the pronounced midline occipital crest, the deep insertion point for a longitudinal ligament, and the extension of the parasphenoid between the orbits and around the basioccipital. Many of these specializations are seen in other durophagous actinopterygians, including Carboniferous eurynotiforms [75] and Eocene gymnodonts [76].

## 5. Conclusion

This paper presents a new genus and species of giant dapediid from the Schesaplana (Canton Grisons, Switzerland), and is the first actinopterygian braincase to be described from this locality. Although represented only by a three-dimensional neurocranium, *Scopulipiscis saxciput* can be confidently identified as a dapediid. CT scanning reveals important internal neurocranial anatomy, including a largely complete endocast, and similarities with the braincase of *Dapedium* suggest that this taxon was also adapted for durophagy. The very large size of this taxon—inferred to be well over 1.5 m in length—increases the known size range of dapediids and suggests that dapediids reached higher levels of diversity than previously anticipated prior to the End-Triassic extinction. An expanded phylogenetic analysis incorporating most dapediid taxa and this new specimen fails to support the monophyly of *Dapedium*, but recovers Dapediidae as a clade of early diverging holosteans, filling a conspicuous gap on the holostean stem [7].

## Supplementary Material

Supplementary Information

## Supplementary Material

Data matrix

## Supplementary Material

Supplementary Figure 1

## Supplementary Material

Supplementary Data

## Data accessibility

Three-dimensional surface files (.ply) and scan data (.tiff stack) are available from MorphoMuseuM (https://doi.org/10.18563/journal.m3.44). Other data files, including the data matrix, are provided as electronic supplementary materials. The ZooBank LSID (Life Science Identifier) for this publication is: urn:lsid:zoobank.org:pub:573AD7A0-7184-4506-A760-BA1632C38479.
