# Supplementary material for: A giant dapediid from the Late Triassic of Switzerland and insights into neopterygian phylogeny
Source: R Soc Open Sci. 2018 Aug 15;5(8):180497. doi: 10.1098/rsos.180497 (PMC6124034; doi:10.1098/rsos.180497)
Supplement: Supplementary Information [file rsos180497supp1.pdf]

Part C. Phylogenetic Analyses

1) General notes. This matrix is based on that of Giles et al. (2017), with 25 additional characters and 18 additional taxa. This matrix encompasses osteichthyan interrelationships, with a strong focus on actinopterygians, and includes non-osteichthyan outgroups. Characters are derived from studies of **gnathostome** (Maisey 1986; Brazeau 2009; Zhu et al. 2013; Giles et al. 2015c), **osteichthyan** (Forey 1980; Schultze & Cumbaa, 2001; Zhu & Schultze 2001; Zhu et al. 2001; Zhu & Yu 2002; Zhu et al. 2006; Zhu et al. 2009; Friedman & Brazeau 2010; Davis et al. 2012; Brazeau & Friedman 2014), **sarcopterygian** (Cloutier & Ahlberg, 1996; Ahlberg & Clack, 1998; Ahlberg & Johanson 1998; Zhu & Ahlberg 2004; Daeschler et al. 2006; Long et al. 2006; Friedman 2007), **actinopterygian** (Patterson 1982; Lauder & Liem, 1983; Gardiner 1984; Gardiner & Schaeffer 1989; Taverne, 1997; Lund et al. 1995; Lund & Poplin, 1997; Coates 1999; Dietze 2000; Lund 2000; Poplin & Lund, 2000; Lund & Poplin 2001; Cloutier & Arratia, 2004; Gardiner et al. 2005; Friedman & Blom, 2006; Long et al., 2008; Arratia 2009; Swartz, 2009; Choo, 2011; Xu et al., 2011; Giles et al. 2015b; Xu et al., 2014; Giles et al. 2017), and **neopterygian** (Patterson 1973; Olsen & McCune 1991; Grande & Bemis 1998; Cavin & Suteethorn 2006; Hurley 2007; Grande 2010; López-Arbarello 2012; Bermúdez-Rochas & Poyato-Ariza 2015; Poyato-Ariza 2015; Thies & Waschkewitz, 2015; Xu & Shen 2015; Xu et al., 2015; Gibson 2016; Xu & Zhao, 2016; López-Arbarello & Sferco 2018) phylogeny.

18 taxa were added to the analysis in order to understand the relationships of dapediids and pycnodonts. These comprise 12 **dapediids**: *Aetholepis mirablis* (Woodward 1895), *Dandya ovalis* (Tintori 1983), *Dapedium caelatum* (Thies & Hauff, 2008; Thies & Herzog, 1999), *Dapedium noricum* (Tintori, 1983), *Dapedium punctatum* (Thies & Herzog, 1999; Thies & Hauff, 2011; Thies & Waschewitz, 2015), *Dapedium stollorum* (Thies & Herzog, 1999; Thies

& Hauff, 2011; Thies & Waschewitz, 2015), *Hemicalypterus weiri* (Schaeffer, 1967; Gibson 2013, 2016), *Heterostrophus phillipsi* (Woodward 1928, 1929), *Paradapedium egertoni* (Jain 1973), *Sargodon tomicus* (Tintori 1983), *Scopulipiscis saxciput*, *Tetragonolepis oldhami* (Jain 1973); and six **pycnodonts**: *Arduafrons prominoris* (Nursall 1999), *Brembodus ridens* (Tintori 1981), *Eomesodon liassicus* (Gardiner 1960), *Gibbodon cenensis* (Tintori 1981), *Mesturus* sp. (Nursall 1999), *Mesturus verrucosus* (Nursall 1999).

In addition, 25 characters have been added. These have a focus on dapediid and pycnodont anatomy, and are both novel and sourced from the literature. Full references and notes are provided below. The present matrix comprises 291 characters and 110 taxa. Characters are ordered by skeletal system, with the exception of newly added characters.

2) Character codes updated from Giles et al. (2017). *Bobasatrania*: c.115, 0 –> -; *Dapedium* sp.: c.173, 0 –>1, c.178, 1 –> 0, c.181, 1 –> 0; *Discoserra*: c.115, 0 –> -; *Heterostrophus*, c.112 1 –> 0; *Hiodon*: c.42, 0 –> -; *Lepisosteus*: c.181, 0 –> 1; *Macrosemimimus*: c.115, 0 –> -; *Paradapedium*: c.112, 1 –>0; *Semionotus*: c.115, 0 –> -; *Tetragonolepis*: c.115, 0 –> -; *Venusichthys*: c.115, 1 –> 2; *Watsonulus*: c.173, 0 –> 1, c.214, 1 –> 0;

3) Character List

General

1. Large dermal plates

(Forey, 1980; Gardiner, 1984; Zhu & Schultze, 2001; Zhu et al., 2001; Zhu & Yu, 2002; Zhu et al., 2006; Friedman, 2007; Brazeau, 2009; Zhu et al., 2009; Friedman & Brazeau; 2010;

Davis et al., 2012; Zhu et al., 2013; Brazeau & Friedman, 2014; Giles et al., 2015b,c; Giles et al., 2017.)

0 absent

1 present

2. Sensory lines

(Brazeau, 2009; Zhu et al., 2013; Giles et al., 2015b; Giles et al., 2017.)

0 preserved as open grooves

1 pass through canals

Dermal skull

3. Premaxilla as distinct ossification

(Hurley et al. 2007; Xu et al. 2014; Giles et al., 2017. Typically, the premaxilla is a short, paired or median bone that contributes to the orbital margin anterior to the maxilla. However, considerable variation is present, and we have attempted to consistently code this variation as laid out here and in the following character descriptions. A premaxilla may be completely absent (e.g. *Acipenser*, *Cyranorhis*) or dorsally expanded into a midline bone (possibly fused with the rostral; e.g. *Bobasatrania*, *Styracopterus*). These two latter states are coded as '1' here.)

0 present

1 absent

4. Premaxillae, contact at midline

(Cloutier & Ahlberg, 1996; Taverne, 1997; Schultze & Cumbaa, 2001; Zhu & Schultze, 2001; Zhu & Yu, 2002; Cloutier & Arratia, 2004; Friedman & Blom, 2006; Zhu et al., 2006; Friedman, 2007; Long et al., 2008; Swartz, 2009; Choo, 2011; Giles et al., 2017. Coded as inapplicable in taxa lacking any ossification in the position typically occupied by the premaxilla (e.g. *Acipenser*, *Cyranorhis*) and where the premaxilla appears fused with the rostral (e.g. *Bobasatrania*, *Styracopterus*).

0 present

1 absent

5. Premaxilla fused at midline

(Xu et al., 2011; Xu et al., 2015; Xu & Zhao, 2016; Giles et al., 2017. Coded as inapplicable in taxa lacking any ossification in the position typically occupied by the premaxilla (e.g. *Acipenser*, *Cyranorhis*) and where the premaxilla appears fused with the rostral (e.g. *Bobasatrania*, *Styracopterus*) and where the premaxilla appears fused with the rostral (e.g. *Bobasatrania*, *Styracopterus*).)

0 absent

1 present

6. Premaxilla

(Friedman, 2007; Giles et al., 2015b; Giles et al., 2017. Coded as inapplicable in taxa lacking any ossification in the position typically occupied by the premaxilla (e.g. *Acipenser*, *Cyranorhis*) and in taxa where the premaxillae do not contact at the midline.)

0 Reaches or extends past anterior margin of orbit

1 Confined to region anterior to orbit

7. Premaxilla contributes to orbital margin

(Cloutier & Ahlberg, 1996; Schultze & Cumbaa, 2001; Zhu & Schultze, 2001; Zhu et al., 2001; Zhu & Yu, 2002; Cloutier & Arratia, 2004; Zhu et al., 2006; Friedman, 2007; Long et al., 2008; Swartz, 2009; Zhu et al., 2009; Xu & Gao, 2011; Zhu et al., 2013; Xu et al., 2014; Giles et al., 2017. Coded as inapplicable in taxa lacking any ossification in the position typically occupied by the premaxilla (e.g. *Acipenser*, *Cyranorhis*), where the premaxilla appears fused with the rostral (e.g. *Bobosatrania*, *Styracopterus*), and where the premaxilla is restricted anterior to the orbit.)

0 absent

1 present

8. Teeth on premaxillae

(Cloutier & Arratia 2004, Xu et al. 2014; Giles et al., 2017. Coded as inapplicable in taxa lacking any ossification in the position typically occupied by the premaxilla (e.g. *Acipenser*, *Cyranorhis*).)

0 present

1 absent

9. Mobile premaxilla

(Arratia 1999; Cavin & Suteethorn 2006; Hurley et al. 2007; Giles et al., 2017. Coded as inapplicable in taxa lacking any ossification in the position typically occupied by the premaxilla (e.g. *Acipenser*, *Cyranorhis*) and where the premaxilla appears fused with the rostral (e.g. *Bobasatrania*, *Styracopterus*).

0 absent

1 present

10. Olfactory nerve pierces premaxilla

(Grande 2010; Xu et al., 2015; Xu & Shen, 2015; Xu & Zhao, 2016; Giles et al., 2017. Coded as inapplicable in taxa lacking any ossification in the position typically occupied by the premaxilla (e.g. *Acipenser*, *Cyranorhis*) and where the premaxilla appears fused with the rostral (e.g. *Bobasatrania*, *Styracopterus*).)

0 absent

1 present

11. Nasal process of premaxilla

(Gardiner & Schaeffer 1989; Olsen & McCune 1991; Gardiner et al. 1996; Gardiner et al. 2005; Cavin & Suteethorn 2006; Hurley et al. 2007; Grande 2010; López-Arbarello 2012; Xu & Wu 2012; Xu et al. 2014; Bermúdez-Rochas & Poyato-Ariza 2015; Poyato-Ariza 2015; Thies & Waschkewitz, 2015; Xu & Shen, 2015; Gibson 2016; Xu & Zhao, 2016; Giles et al., 2017; López-Arbarello & Sferco 2018. Coded as inapplicable in taxa lacking any ossification in the position typically occupied by the premaxilla (e.g. *Acipenser*, *Cyranorhis*) and where the premaxilla appears fused with the rostral (e.g. *Bobasatrania*, *Styracopterus*).)

0 absent

1 short

2 long, reaches skull roof

12. Sensory canal on premaxilla

(Giles et al., 2017. Coded as inapplicable in taxa lacking any ossification in the position typically occupied by the premaxilla (e.g. *Acipenser*, *Cyranorhis*) and where the premaxilla appears fused with the rostral (e.g. *Bobasatrania*, *Styracopterus*).)

0 present

1 absent

13. Postrostrals (element[s] immediately anterior to frontals but not in contact with premaxillae)

(Cloutier & Ahlberg, 1996; Taverne, 1997; Lund, 2000; Schultze & Cumbaa, 2001; Zhu & Schultze, 2001; Lund & Poplin, 2002; Cloutier & Arratia, 2004; Friedman & Blom, 2006; Long et al., 2008; Swartz, 2009; Choo, 2011; Xu et al., 2014; Giles et al., 2015b; Giles et al., 2017.)

0 present

1 absent

14. Single median dermal bone capping snout

(Gardiner & Schaeffer, 1989; Taverne, 1997; Friedman & Blom, 2006; Long et al., 2008; Swartz, 2009; Choo, 2011; Giles et al., 2015b; Poyato-Ariza 2015; Giles et al., 2017.)

0 absent

1 present

15. Median rostal

(Gardiner et al. 1996; Hurley et al. 2007; Giles et al., 2017.)

0 plate-like

1 tube-like

16. Pores for rostral organ

(Friedman, 2007; Giles et al., 2015b; Giles et al., 2017.)

0 absent

1 present

17. Nasal bone as single consolidated ossification (i.e. bone(s) carrying supraorbital canal between premaxilla and anterior margin of frontals)

(Taverne, 1997; Schultze & Cumbaa, 2001; Friedman & Blom, 2006; Long et al., 2008; Swartz, 2009; Choo, 2011; Giles et al., 2015b; Giles et al., 2017.)

0 absent

1 present

18. Contact of nasals on midline

(Giles et al., 2017.)

0 separated by dermal bones

1 contacting or separated by gap unfilled by bone

19. Nasal contributes to orbital margin

(Xu & Wu 2012; Xu et al. 2014; Xu & Zhao, 2016; Giles et al., 2017; López-Arbarello & Sferco 2018.)

0 absent

1 present

20. Mesial margin of (anterior) nasal

(Lund et al., 1995; Ahlberg & Johanson, 1998; Ahlberg et al., 2000; Lund, 2000; Poplin & Lund, 2000; Schultze & Cumbaa, 2001; Lund & Poplin, 2002; Cloutier & Arratia, 2004; Zhu

& Ahlberg, 2004; Daeschler et al., 2006; Long et al., 2006; Zhu et al., 2006; Zhu et al., 2009; Choo, 2011; Giles et al., 2015b; Giles et al., 2017; López-Arbarello & Sferco 2018.)

0 not notched

1 notched

21. Posterior nostril in complete communication with orbital fenestra

(Friedman & Blom, 2006; Long et al., 2008; Choo, 2011; Giles et al., 2015b; Giles et al., 2017. This character refers to the opening of the posterior nostril with reference to the surrounding dermal bones.)

0 absent

1 present

22. Posterior nostril - contribution to margin by premaxillae

(Friedman & Blom, 2006; Long et al., 2008; Choo, 2011; Giles et al., 2015b; Giles et al., 2017.)

0 absent

1 present

23. Tectals (sensu Cloutier & Ahlberg 1996, not counting the posterior tectal of Jarvik)

(Lund et al., 1995; Cloutier & Ahlberg, 1996; Lund, 2000; Schultze & Cumbaa, 2001; Zhu & Schultze, 2001; Zhu et al., 2001; Lund & Poplin, 2002; Zhu & Yu, 2002; Cloutier & Arratia, 2004; Zhu et al., 2006; Friedman, 2007; Swartz, 2009; Zhu et al., 2009; Zhu et al., 2013; Giles et al., 2015b; Giles et al., 2017.)

0 absent

1 present

24. Dermal intracranial joint

(Cloutier & Ahlberg, 1996; Ahlberg & Johanson, 1998; Zhu & Ahlberg, 2004; Zhu & Schultze, 2001; Zhu et al., 2001; Zhu & Yu, 2002; Daeschler et al., 2006; Long et al., 2006; Zhu et al., 2006; Friedman, 2007; Brazeau, 2009; Zhu et al., 2009; Choo, 2011; Davis et al., 2012; Zhu et al., 2013; Giles et al., 2015b; Giles et al., 2017.)

0 absent

1 present

25. Pineal foramen

(Cloutier & Ahlberg, 1996; Taverne, 1997; Schultze & Cumbaa, 2001; Zhu & Schultze, 2001; Zhu & Yu, 2002; Friedman & Blom, 2006; Friedman, 2007; Long et al., 2008; Brazeau, 2009; Swartz, 2009; Davis et al., 2012; Zhu et al., 2013; Xu et al., 2014; Giles et al., 2015b,c; Giles et al., 2017. A pineal foramen is variably present in *Cheirolepis canadensis* (Pearson & Westoll, 1979; Arratia & Cloutier, 1996), *C. trailli* (Pearson & Westoll, 1979), *Kentuckia deani* (Rayner, 1951) and *Meemannia* (Zhu et al., 2010), and these taxa are coded '0/1' to reflect this polymorphism.)

0 present

1 absent

26. Pineal eminence

(Friedman, 2007; Zhu et al., 2009; Giles et al., 2015b; Giles et al., 2017. Can only be coded in taxa that lack a pineal foramen.)

0 absent

1 present

27. Shape of parietals (sarcopterygian postparietals):

(Dietze, 2000; Schultze & Cumbaa, 2001; Cloutier & Arratia, 2004; Friedman & Blom, 2006; Long et al., 2008; Swartz, 2009; Choo, 2011; Xu et al., 2014; Giles et al., 2015b; Giles et al., 2017.)

0 rectangular, with long axis parallel to midline

1 quadrate

28. Relative lengths of frontals and parietals (sarcopterygian parietals and postparietals)

(Lund et al., 1995; Taverne, 1997; Dietze, 2000; Lund, 2000; Poplin & Lund, 2000; Schultze & Cumbaa, 2001; Lund & Poplin, 2002; Cloutier & Arratia, 2004; Friedman & Blom, 2006; Zhu et al., 2006; Long et al., 2008; Swartz, 2009; Choo, 2011; López-Arbarello 2012; Xu et al., 2014; Bermúdez-Rochas & Poyato-Ariza 2015; Thies & Waschkewitz, 2015; Gibson 2016; Giles et al., 2015b; Giles et al., 2017; López-Arbarello & Sferco 2018.)

0 frontal shorter than parietal

1 frontal approximately equal to parietal

2 frontal longer than parietal

29. Frontals broad posteriorly and tapering anteriorly

(Arratia 1999; López-Arbarello 2012; Bermúdez-Rochas & Poyato-Ariza 2015; Thies & Waschkewitz, 2015; Gibson 2016; Giles et al., 2017; López-Arbarello & Sferco 2018.)

0 absent

1 present

30. Anterior pit line

(Giles et al., 2015b,c; Giles et al., 2017. Although not figured, an anterior pit line is described for *Miguashaia* (Cloutier 1996).)

0 absent

1 present

31. Otic canal extends through parietals

(Giles et al., 2015b,c; Giles et al., 2017.)

0 absent

1 present

32. Junction between supraorbital and infraorbital canals

(López-Arbarello 2012; Bermúdez-Rochas & Poyato-Ariza 2015; Poyato-Ariza 2015; Giles et al., 2017; López-Arbarello & Sferco 2018. The supraorbital canal may terminate in the frontal/parietal, or it may become confluent with the infraorbital canal. The exact position of this junction is highly variable, and typically occurs in the region of the frontal, dermosphenotic, dermopterotic.)

0 absent

1 present

33. Anterior branch of infraorbital sensory canal

(Giles et al., 2017. In some taxa (e.g. *Dapedium*), the infraorbital canal continues anteriorly above the orbit a short way.)

0 absent

1 present

34. Tabular

(Lund et al., 1995; Cloutier & Ahlberg, 1996; Schultze & Cumbaa, 2001; Zhu & Schultze, 2001; Cloutier & Arratia, 2004; Long et al., 2008; Swartz, 2009; Giles et al., 2015b; Giles et al., 2017.)

0 present

1 absent

35. Tabular pit line

(Giles et al., 2015b,c; Giles et al., 2017.)

0 absent

1 present

36. Number of bones carrying otic portion of lateral line canal between dermosphenotic and posterior edge of skull roof.

(Gardiner & Schaeffer 1989; Cloutier & Arratia, 2004; Hurley et al. 2007; Choo, 2011; Giles et al., 2015b; Xu & Zhao, 2016; Giles et al., 2017. This character is reformulated from Choo's character 'Dermopterotic: present/absent'. Rather than designating bones as an intertemporal (or supratemporal or dermosphenotic) a priori, we consider the number of bones carrying the otic portion of the lateral line canal between the dermosphenotic and the posterior edge of the skull roof. Where two bones are present, these are treated as the intertemporal and supratemporal; where only one is present, this is treated as the dermopterotic. Anamestic bones between the dermosphenotic and frontal are not included in this count.)

0 at least two (i.e. intertemporal and supratemporal)

1 one (i.e. dermopterotic)

37. Intertemporal - relative length

(Taverne, 1997; Friedman & Blom, 2006; Choo, 2011; Giles et al., 2015b; Giles et al., 2017. Coded as inapplicable in taxa with a dermopterotic. Coded as inapplicable in taxa with a dermopterotic.)

0 shorter than supratemporal

1 of similar length to supratemporal

2 longer than supratemporal

38. Intertemporal - contact with supratemporal anterior to that between frontal and parietal

(Friedman & Blom, 2006; Choo, 2011; Giles et al., 2015b; Giles et al., 2017. Coded as inapplicable in taxa with a dermopterotic.)

0 absent

1 present

39. Intertemporal contacts nasal

(Xu & Gao, 2011; Xu et al., 2014; Giles et al., 2015b; Giles et al., 2017. Coded as inapplicable in taxa with a dermopterotic.)

0 absent

1 present

40. Supratemporal - narrow anterolateral flange forming ventral margin of spiracular opening

(Choo, 2011; Giles et al., 2015b; Giles et al., 2017. Coded as inapplicable in taxa with a dermopterotic.)

0 absent

1 present

41. Parietal fused to dermopterotic

(Xu & Gao 2011; Xu et al. 2014; Giles et al., 2017. Coded as inapplicable in taxa with a separate intertemporal and supertemporal, and in taxa lacking these bones entirely.)

0 absent

1 present

42. Bone carrying otic portion of lateral line canal extends past posterior margin of parietals

(Lu et al. 2016; Giles et al., 2017.)

0 absent

1 present

43. Number of paired extrascapulars

(Gardiner & Schaeffer, 1989; Lund et al., 1995; Cloutier & Ahlberg, 1996; Grande & Bemis 1998; Coates, 1998; Lund, 2000; Poplin & Lund, 2000; Schultze & Cumbaa, 2001; Zhu & Schultze, 2001; Lund & Poplin, 2002; Cloutier & Arratia, 2004; Friedman & Blom, 2006; Long et al., 2008; Swartz, 2009; Choo, 2011; López-Arbarello 2012; Zhu et al., 2013; Bermúdez-Rochas & Poyato-Ariza 2015; Poyato-Ariza 2015; Giles et al., 2015b; Thies & Waschkewitz, 2015; Xu et al., 2015; Gibson 2016; Xu & Zhao, 2016; Giles et al., 2017; López-Arbarello & Sferco 2018.)

0 one pair

1 two pairs

2 three or more pairs

44. Extrascapular reaches lateral edge of skull roof

(Giles et al., 2015b,c; Giles et al., 2017. The skull roof of *Moythomasia* as shown in Fig. 103 (Gardiner, 1984) is a restoration (see also Choo 2015: fig. 13). The only skull roof directly figured by Gardiner does not preserve the extrascapulars in situ (fig. 83). However, in specimens viewed by us, as well as in published photos of articulated material (e.g. Choo 2015: fig 8), the lateral extrascapular of *M. durgaringa* is clearly excluded from the lateral margin of the skull roof.)

0 absent

1 present

45. Single median extrascapular

(Dietze, 2000; Cloutier & Arratia, 2004; Long et al., 2008; Swartz, 2009; Choo, 2011; Xu & Gao, 2011; Zhu et al., 2013; Xu et al., 2014; Giles et al., 2015b; Giles et al., 2017.)

0 present

1 absent

46. Extrascapulae contact each other at midline

(Giles et al., 2015b,c; Giles et al., 2017. Inapplicable for taxa that possess a median extrascapular, as it is logically impossible for the lateral extrascapulae to meet in the midline.)

0 absent

1 present

47. Medially-directed branch of sensory canal in extrascapulae

(Choo, 2011; Giles et al., 2015b; Giles et al., 2017.)

0 present

1 absent

48. Extratemporal

(Cloutier & Ahlberg, 1996; Ahlberg & Johanson, 1998; Zhu & Schultze, 2001; Zhu et al., 2001; Zhu & Yu, 2002; Zhu & Ahlberg, 2004; Daeschler et al., 2006; Long et al., 2006; Zhu et al., 2006; Friedman, 2007; Zhu et al., 2009; Giles et al., 2015b; Giles et al., 2017.)

0 absent

1 present

49. Antorbital bone

(Gardiner & Schaeffer 1989; Lund et al. 2000; Cloutier & Arratia, 2004; Hurley et al. 2007; Choo, 2011; Xu & Gao, 2011; Xu et al., 2014; Giles et al., 2015b; Giles et al., 2017; López-Arbarello & Sferco 2018.)

0 absent

1 present

50. Tube-like canal bearing anterior arm of antorbital:

(Grande 2010; Xu & Wu 2012; Xu et al. 2014; Poyato-Ariza 2015; Xu & Shen, 2015; Xu & Zhao, 2016; Giles et al., 2017; López-Arbarello & Sferco 2018.)

0 absent

1 present

51. Infraorbitals

(Cloutier & Arratia, 2004; Gardiner et al., 2005; Choo, 2011; Xu & Gao, 2011; Xu et al., 2014; Giles et al., 2015b; Giles et al., 2017; López-Arbarello & Sferco 2018.)

0 one

1 two

2 more than two

52. Anterior expansion of lacrimal

(Taverne, 1997; Friedman & Blom, 2006; Long et al., 2008; Swartz, 2009; Choo, 2011; Giles et al., 2015b; Giles et al., 2017.)

0 absent

1 present

53. Notch in anterior margin of jugal

(Cloutier & Arratia, 2004; Friedman & Blom, 2006; Long et al., 2008; Swartz, 2009; Choo, 2011; Giles et al., 2015b; Giles et al., 2017; Xu et al., 2014.

0 absent

1 present

54. Suborbitals (non-canal bearing ossifications separating jugal and maxilla)

(Gardiner & Schaeffer 1989; Olsen & McCune 1991; Taverne, 1997; Schultze & Cumbaa, 2001; Friedman & Blom, 2006; Long et al., 2008; Choo, 2011; López-Arbarello 2012; Xu & Gao 2011; Bermúdez-Rochas & Poyato-Ariza 2015; Poyato-Ariza 2015; Giles et al., 2015b;

Thies & Waschkewitz, 2015; Xu et al., 2015; Gibson 2016; Xu & Zhao, 2016; Giles et al., 2017; López-Arbarello & Sferco 2018.)

0 absent

1 one

2 two

3 three or more

55. Multiple rami of infraorbital canal in jugal

(Giles et al., 2015b,c; Giles et al., 2017. Multiple branches radiate from the infraorbital canal in the jugal of many Carboniferous and younger actinopterygians.)

0 absent

1 present

56. Dermosphenotic with distinct posterior ramus

(Gardiner & Schaeffer, 1989; Coates, 1998; Schultze & Cumbaa, 2001; Cloutier & Arratia, 2004; Friedman & Blom, 2006; Zhu et al., 2006; Long et al., 2008; Zhu et al., 2009; Choo, 2011; Giles et al., 2015b; Xu et al., 2015; Giles et al., 2017. The dermosphenotic of *Moythomasia* illustrated by (Gardiner 1984, fig 69) lacks a posterior limb, but this is from a small individual and most likely reflects ontogenetic variability, with a posterior limb being developed in larger individuals (B. Choo, pers. comm.; Choo 2015: fig. 8). The posterior limb of the dermosphenotic is variably developed in *Mesopoma* (Coates, 1999), so this taxon is scored '0/1' to reflect this polymorphism.)

0 absent

1 present

57. Dermosphenotic- contact with frontals blocked by intertemporal or dermopterotic

(Friedman & Blom, 2006; Choo, 2011; Giles et al., 2015b; Giles et al., 2017.)

0 absent

1 present

58. Supraorbital

(Gardiner & Schaeffer 1989; Grande & Bemis 1998; Hurley et al. 2007; Xu & Gao 2011; Xu et al. 2014; Poyato-Ariza 2015; Xu et al., 2015; Xu & Zhao, 2016; Giles et al., 2017; López-Arbarello & Sferco 2018.)

0 absent

1 one or two

2 three or more

59. Anterior-most infraorbital anterior to orbit (i.e. does not contribute to orbital margin)

(Olsen & McCune 1991; Cavin & Suteethorn 2006; López-Arbarello 2012; Bermúdez-Rochas & Poyato-Ariza 2015; Thies & Waschkewitz, 2015; Gibson 2016; Giles et al., 2017; López-Arbarello & Sferco 2018.)

0 absent

1 present

60. Three or more lachrymals

(Grande 2010; Xu & Wu 2012; Xu et al. 2014, 2015; Bermúdez-Rochas & Poyato-Ariza 2015; Poyato-Ariza 2015; Thies & Waschkewitz, 2015; Gibson 2016; Xu & Zhao, 2016;

Giles et al., 2017. The first lachrymal is regarded here as the anteriormost canal-bearing bone that contributes to orbital margin.)

0 absent

1 present

61. Circumorbital ring

(Wiley 1976; López-Arbarello 2012; Bermúdez-Rochas & Poyato-Ariza 2015; Poyato-Ariza 2015; Thies & Waschkewitz, 2015; Gibson 2016; Giles et al., 2017; López-Arbarello & Sferco 2018.)

0 Supraorbitals do not contact infraorbitals at the anterior rim of the orbit.

1 Supraorbitals contact infraorbitals, closing the orbit.

62. Jugal canal

(Patterson, 1982; Lauder & Liem, 1983; Gardiner, 1984; Cloutier & Arratia, 2004; Brazeau, 2009; Friedman & Brazeau, 2010; Choo, 2011; Davis et al., 2012; Zhu et al., 2013; Giles et al., 2015b,c; Giles et al., 2017.)

0 absent

1 present

63. Dermohyal

(Patterson, 1982; Gardiner & Schaeffer, 1989; Lund et al., 1995; Cloutier & Ahlberg, 1996; Coates, 1998; Dietze, 2000; Lund, 2000; Schultze & Cumbaa, 2001; Zhu & Schultze, 2001; Zhu et al., 2001; Lund & Poplin, 2002; Zhu & Yu, 2002; Cloutier & Arratia, 2004; Gardiner et al., 2005; Friedman & Blom, 2006; Zhu et al., 2006; Friedman, 2007; Long et al., 2008; Swartz, 2009; Zhu et al., 2009; Choo, 2011; Xu & Gao, 2011; Xu et al., 2014; 2015; Giles et

al., 2015b; Xu & Zhao, 2016; Giles et al., 2017. This region of the cheek is missing in *Coccocephalichthys* (Poplin, 1974; Poplin & Véran, 1996), and was presumably removed by Watson (1925) when he first described the specimen. It is unclear from the surviving cast whether a dermohyal and/or accessory operculum were present, and as such this taxon is coded as '?'.)

0 absent

1 present

64. Head of dermohyal projects above dorsal margin of operculum

(Giles et al., 2015b,c; Giles et al., 2017. The dermohyal is not preserved in *Melanecta* (Coates, 1998), but it is clear from the surrounding bones that it would not have projected above the dorsal surface of the operculum.)

0 absent

1 present

65. Dermohyal

(Gardiner et al., 2005; Coates, 1999; Xu & Gao, 2011; Xu et al., 2014; Giles et al., 2015b; Giles et al., 2017.)

0 fused to hyomandibular

1 separate from hyomandibular

66. Complete enclosure of spiracle by bones bearing otic and infraorbital canals

(Friedman, 2007; Zhu et al., 2009; Giles et al., 2015b; Giles et al., 2017.)

0 absent

1 present

Jaws and palate

67. Maxilla

(Zhu & Yu, 2002; Friedman, 2007; Xu et al., 2014, 2015; Giles et al., 2015b; Xu & Zhao, 2016; Giles et al., 2017; López-Arbarello & Sferco 2018.)

0 absent

1 present

68. Expanded dorsal lamina of maxilla

(Lund et al., 1995; Lund, 2000; Poplin & Lund, 2000; Schultze & Cumbaa, 2001; Zhu & Schultze, 2001; Zhu et al., 2001; Zhu & Yu, 2002; Lund & Poplin, 2002; Cloutier & Arratia, 2004; Zhu et al., 2006; Friedman, 2007; Zhu et al., 2009; Zhu et al., 2013; Giles et al., 2015b,c; Giles et al., 2017; López-Arbarello & Sferco 2018.)

0 absent

1 present

69. Contribution by maxilla to posterior margin of cheek

(Friedman, 2007; Zhu et al., 2009; Zhu et al., 2013; Giles et al., 2015b,c; Giles et al., 2017; López-Arbarello & Sferco 2018.)

0 absent

1 present

70. Sensory canal/pit line associated with maxilla

(Grande & Bemis 1998; Friedman, 2007; Zhu et al., 2009; Zhu et al., 2013; Giles et al., 2015b; Giles et al., 2017.)

0 absent

1 present

71. Teeth on maxilla

(Poyato-Ariza & Wenz, 2002; Cloutier & Arratia, 2004; López-Arbarello 2012; Xu et al. 2014, 2015; Bermúdez-Rochas & Poyato-Ariza 2015; Poyato-Ariza 2015; Thies & Waschkewitz, 2015; Gibson 2016; Xu & Zhao, 2016; Giles et al., 2017; Cawley & Kriwet 2018; López-Arbarello & Sferco 2018.)

0 present

1 absent

72. Mobile maxilla in cheek

(Patterson 1973; Gardiner & Schaeffer 1989; Olsen & McCune 1991; Gardiner et al. 1996; Gardiner et al. 2005; Coates 1999; Hurley et al. 2007; Xu & Gao 2011; Poyato-Ariza 2015; Xu et al. 2014, 2015; Xu & Zhao, 2016; Giles et al., 2017.)

0 absent

1 present

73. Peg-like anterior process of maxilla

(Grande 2010; Xu & Wu 2012; Xu et al. 2014; Poyato-Ariza 2015; Giles et al., 2017.)

0 absent

1 present

74. Posterior maxillary notch

(Grande & Bemis 1998; Xu & Wu 2012; Xu et al. 2014, 2015. Arratia 2013; Xu & Zhao, 2016; Giles et al., 2017; López-Arbarello & Sferco 2018.)

0 absent

1 present

75. Supramaxilla

(Grande & Bemis 1998; Gardiner & Schaeffer 1989; Olsen & McCune 1991; Gardiner et al. 1996; Gardiner et al. 2005; Coates 1999; Hurley et al. 2007; Xu & Gao 2011; Xu et al. 2014, 2015; Bermúdez-Rochas & Poyato-Ariza 2015; Poyato-Ariza 2015; Xu & Shen, 2015; Thies & Waschkewitz, 2015; Gibson 2016; Xu & Zhao, 2016; Giles et al., 2017; López-Arbarello & Sferco 2018.)

0 absent

1 one

2 two

76. Course of mandibular canal

(Friedman & Blom, 2006; Long et al., 2008; Swartz, 2009; Choo, 2011; Giles et al., 2015b; Giles et al., 2017.)

0 traces ventral margin of jaw along entire length

1 arches dorsally in anterior half of jaw

77. Mandibular canal reaches anterior margin of mandible

(Giles et al., 2015b,c; Giles et al., 2017.)

0 present

1 absent

78. Mandibular canal

(Patterson, 1982; Cloutier & Ahlberg, 1996; Coates, 1998; Schultze & Cumbaa, 2001; Zhu & Schultze, 2001; Zhu et al., 2001; Zhu & Yu, 2002; Cloutier & Arratia, 2004; Zhu et al., 2006; Friedman, 2007; Zhu et al., 2009; Choo, 2011; Zhu et al., 2013; Giles et al., 2015b; Giles et al., 2017.)

0 primarily carried by infradentaries

1 primarily carried by dentary

79. Relative length of dentary

(Ahlberg & Johanson, 1998; Zhu et al., 2001; Zhu & Yu, 2002; Zhu & Ahlberg, 2004; Friedman, 2007; Zhu et al., 2009; Giles et al., 2015b; Giles et al., 2017.)

0 long (constitutes most of the length of the lower jaw)

1 short (constitutes less than half of jaw length)

80. Teeth on dentary

(Cloutier & Arratia 2004, Xu et al. 2014; Giles et al., 2017; López-Arbarello & Sferco 2018.)

0 present

1 absent

81. Dentary with conspicuously reflexed distal tip

(Friedman & Blom, 2006; Long et al., 2008; Swartz, 2009; Choo, 2011; Giles et al., 2015b; Giles et al., 2017.)

0 absent

1 present

82. Enlarged series of parasymphysial teeth on dentary

(Friedman & Blom, 2006; Long et al., 2008; Swartz, 2009; Choo, 2011; Giles et al., 2017.)

0 absent

1 present

83. Facet for parasymphysial tooth-whorl on anterior dentary

(Choo, 2011; Giles et al., 2015b; Giles et al., 2017.)

0 present

1 absent

84. Teeth of outer dental arcade

(Friedman, 2007; Giles et al., 2015b; Poyato-Ariza 2015; Giles et al., 2017; López-Arbarello & Sferco 2018. Coates (1998) states that the maxilla of *Melanecta* bears large teeth interspersed with smaller teeth, but it is unclear how these teeth are arranged. As such, this taxon is coded '?'.)

0 several rows of disorganized teeth

1 two rows, with large teeth lingually and small teeth labially

2 single row of teeth

85. Jaw margins overlain by lateral lamina

(Giles et al. 2017. In *Styracopterus*, *Fouldenia* and *Amphicentrum*, a lateral lamina of bone obscures the maxillary dentition (Sallan & Coates 2013).)

0 absent

1 present

86. Acrodin caps on teeth

(Patterson, 1982; Gardiner, 1984; Maisey, 1986; Gardiner & Schaeffer, 1989; Cloutier & Ahlberg, 1996; Taverne, 1997; Coates, 1999; Poplin & Lund, 2000; Schultze & Cumbaa, 2001; Zhu & Schultze, 2001; Zhu et al., 2001; Zhu & Yu, 2002; Cloutier & Arratia, 2004; Gardiner et al., 2005; Friedman & Blom, 2006; Zhu et al., 2006; Friedman, 2007; Long et al., 2008; Zhu et al., 2009; Friedman & Brazeau, 2010; Choo, 2011; Xu & Gao, 2011; Zhu et al., 2013; Xu et al., 2014; Giles et al., 2015b,c; Giles et al., 2017.)

0 absent

1 present

87. Plicidentine

(Zhu & Yu 2002; Friedman 2007; López-Arbarello 2012; Bermúdez-Rochas & Poyato-Ariza 2015; Poyato-Ariza 2015; Thies & Waschkewitz, 2015; Gibson 2016; Giles et al., 2017; López-Arbarello & Sferco 2018.)

0 absent

1 present

88. Ossification of mentomeckelian region:

(Friedman & Blom, 2006; Long et al., 2008; Swartz, 2009; Grande 2010; Choo, 2011; Xu et al., 2014; Giles et al., 2015b; Poyato-Ariza 2015; Giles et al., 2017.)

0 present

1 absent

89. Number of infradentaries

(Friedman & Blom, 2006; Friedman, 2007; Long et al., 2008; Choo, 2011; Xu & Gao, 2011; Xu et al., 2014; Giles et al., 2015b; Poyato-Ariza 2015; Giles et al., 2017; López-Arbarello & Sferco 2018.)

0 more than two

1 two (angular and surangular)

2 one (angular only)

90. Coronoids (sensu stricto, excluding parasymphysial tooth whorl or anterior coronoid)

(Schultze and Cumbaa, 2001; Zhu and Schultze, 2001; Zhu et al., 2001; Zhu and Yu, 2002; Zhu et al., 2006; Friedman, 2007; Zhu et al., 2009; Giles et al., 2015b,c; Poyato-Ariza 2015; Giles et al., 2017; López-Arbarello & Sferco 2018.)

0 present

1 absent

91. Number of coronoids

(Ahlberg & Clack, 1998; Daeschler et al., 2006; Long et al., 2006; Friedman, 2007; Zhu et al., 2009; Zhu et al., 2013; Giles et al., 2015b; Giles et al., 2017. A single specimen of *Pteronisculus stensioi* has at least five or six coronoids anterior to the prearticular region. However, these appear to correspond to the three coronoids present in most specimens, so the taxon is coded here as '2'. Two coronoids are reported in *Boreosomus* (Nielsen, 1942).)

0 five

1 four or more

2 three

3 two

4 one

92. Posterior coronoid

(Cloutier & Ahlberg, 1996; Ahlberg & Johanson, 1998; Zhu & Ahlberg, 2004; Daeschler et al., 2006; Long et al., 2006; Giles et al., 2015b; Giles et al., 2017.)

0 morphologically similar to anterior coronoids

1 expanded

93. Coronoid process of lower jaw

(Patterson 1973; Gardiner & Schaeffer 1989; Olsen & McCune 1991; Poyato-Ariza & Wenz, 2002; Zhu & Yu, 2002; Friedman, 2007; Friedman, 2007; Xu & Gao 2011; Xu et al. 2014, 2015; Giles et al., 2015b; Poyato-Ariza 2015; Xu & Zhao, 2016; Giles et al., 2017; Cawley & Kriwet 2018; Cawley & Kriwet 2018; López-Arbarello & Sferco 2018.)

0 absent

1 present

94. Coronoid process contributed to by

(Modified from Patterson 1973; Olsen & McCune 1991; Gardiner et al. 2005; Poyato-Ariza 2015; Giles et al., 2017; López-Arbarello & Sferco 2018.)

0 prearticular only

1 surangular only

2 dentary plus postdentary bones

3 angular only

95. Leptolepid notch

(Arratia 2013; Giles et al., 2017; López-Arbarello & Sferco 2018. A distinct notch in the posterior margin of the dentary is seen in taxa such as *Leptolepis*.)

0 absent

1 present

96. Symplectic involvement in jaw joint

(Grande & Bemis 1998; Olsen & McCune 1991; Grande 2010; Xu & Wu 2012; Xu et al. 2014, 2015; López-Arbarello 2012; Bermúdez-Rochas & Poyato-Ariza 2015; Poyato-Ariza 2015; Thies & Waschkewitz, 2015; Gibson 2016; Xu & Zhao, 2016; Giles et al., 2017; López-Arbarello & Sferco 2018.)

0 absent

1 present

97. Retroarticular process

(Friedman, 2007; Giles et al., 2015b; Giles et al., 2017.)

0 present

1 absent

98. Palatal bite

(Giles et al., 2017.)

0 absent

1 present

99. Palatal articulation with basipterygoid process

(Revised from Friedman, 2007; Brazeau, 2009; Zhu et al., 2009; Friedman & Brazeau, 2010; Davis et al., 2012; Zhu et al., 2013; Giles et al., 2015b,c; Giles et al., 2017; López-Arbarello & Sferco 2018. This character is expanded from previous formulations, which only considered whether a basipterygoid fenestra was absent or present. Where a basipterygoid process is absent, the dorsal margin of the palate may be flat, or the metapterygoid may bear a distinct notch.)

0 articulation not obvious

1 via basipterygoid fenestra

2 via metapterygoid process/notch

100. Palatoquadrate ossifications

(Giles et al., 2017.)

0 comineralized

1 separate ossification centers

101. Lateral process of ectopterygoid

(Giles et al., 2017.)

0 absent

1 present

102. Palatoquadrate symphysis

(Giles et al., 2017. This character captures whether the palatoquadrates contact at the midline.

0 absent

1 present

103. Dorsal margin of palate

(Giles et al., 2017.)

0 high posterior extension

1 flat dorsal margin

104. Metapterygoid posterior to quadrate

(Giles et al., 2017.)

0 absent

1 present

105. Number of dermopalatines

(Friedman, 2007; Giles et al., 2015b; Giles et al., 2017.)

0 multiple

1 single

106. Prearticular

(Poyato-Ariza 2015; Giles et al., 2017; López-Arbarello & Sferco 2018.)

0 present

1 absent

107. Vomers

(Patterson 1973; Olsen & McCune 1991; López-Arbarello 2012; Arratia 2013; Xu & Wu, 2012; Bermúdez-Rochas & Poyato-Ariza 2015; Poyato-Ariza 2015; Thies & Waschkewitz, 2015; Gibson 2016; Xu & Zhao, 2016; Giles et al., 2017; López-Arbarello & Sferco 2018.)

0 paired

1 single

108. Vomer sutured to parasphenoid

(Patterson 1973; Olsen & McCune 1991; Hurley et al. 2007; Giles et al., 2017.)

0 absent

1 present

Operculogular system

109. Accessory operculum

(Schultze & Cumbaa, 2001; Cloutier & Arratia, 2004; Friedman & Blom, 2006; Long et al., 2008; Swartz, 2009; Giles et al., 2015b; Giles et al., 2017. This region of the cheek was removed in Coccocephalichthys (Poplin, 1974; Poplin & Véran, 1996), presumably by Watson (1925) when he first described the specimen. It is unclear from the surviving cast whether a dermohyal and/or accessory operculum were present, and as such this taxon is coded as '?'.)

0 absent

1 present

110. Operculum - relative size

(Modified from Lund et al., 1995; Lund, 2000; Lund & Poplin, 2002; Cloutier & Arratia, 2004; Long et al., 2008; Swartz, 2009; Choo, 2011; López-Arbarello 2012; Bermúdez-Rochas & Poyato-Ariza 2015; Xu et al., 2015; Giles et al., 2015b; Thies & Waschkewitz, 2015; Gibson 2016; Xu & Zhao, 2016; Giles et al., 2017; López-Arbarello & Sferco 2018.)

0 at least twice as high as suboperculum

1 subequal

2 smaller than suboperculum

111. Subopercle

(Poyato-Ariza & Wenz, 2002; Xu et al. 2014; Giles et al., 2017; Cawley & Kriwet 2018.)

0 present

1 absent

112. Anterodorsal process of suboperculum

(Long et al., 2008; Choo, 2011; López-Arbarello 2012; Bermúdez-Rochas & Poyato-Ariza 2015; Giles et al., 2015b; Thies & Waschkewitz, 2015; Poyato-Ariza 2015; Gibson 2016; Giles et al., 2017; López-Arbarello & Sferco 2018. An anterodorsal process is present some non-neopterygian actinopterygians and 'semionotiforms'. Although these processes do not appear to be homologous they are coded within the character.)

0 absent

1 present

113. Anteroventral process of suboperculum

(Giles et al., 2017.)

0 absent

1 present

114. Number of cheek bones bearing pre-opercular canal posterior to jugal (Friedman, 2007; Zhu et al., 2009; Zhu et al., 2013; Giles et al., 2015b; Xu & Zhao, 2016; Giles et al., 2017.)

0 one

1 multiple

2 series of small ossicles

115. Preoperculum orientation

(Modified from Patterson 1973; Olsen & McCune 1991; Grande & Bemis 1998; Gardiner et al., 2005; Swartz 2009; López-Arbarello 2012; Bermúdez-Rochas & Poyato-Ariza 2015; Thies & Waschkewitz, 2015; Poyato-Ariza 2015; Gibson 2016; Giles et al., 2017; López-Arbarello & Sferco 2018. This character is reformulated from its original (compound) formulation, which considered both maxilla and preoperculum shape. Primitively in actinopterygians the preoperculum is wholly or partially developed dorsal to the maxilla as an anterodorsal-posteroventrally oriented bone, either with (e.g. *Mimipiscis*, *Moythomasia*) or without (e.g. *Cheirolepis*) a dorsoventrally oriented limb. The preoperculum may also be near-vertical, with no distinct anterodorsal or anteroventral extensions (e.g. *Boreosomus*, *Peltopleurus*), or developed as an anteroventral-posterodorsally-directed bone largely ventral to the maxilla (e.g. *Discoserra*, *Propterus*).)

0 prounounced dorsal limb

1 vertical

2 pronounced ventral limb

116. Junction between preopercular and more anterior cheek bones

(Modified from López-Arbarello 2012; Bermúdez-Rochas & Poyato-Ariza 2015; Poyato-Ariza 2015; Thies & Waschkewitz, 2015; Gibson 2016; Giles et al., 2017; López-Arbarello & Sferco 2018.)

0 Infraorbitals (including jugal) or suborbitals suture with or abut preopercular

1 Infraorbitals (including jugals) and suborbitals broadly overlap preopercular

117. Posterior border of preopeculum notched ventrally

(López-Arbarello 2012; Bermúdez-Rochas & Poyato-Ariza 2015; Giles et al., 2017; Thies & Waschkewitz, 2015; Gibson 2016; López-Arbarello & Sferco 2018.)

0 absent

1 present

118. Interopercle

(Patterson 1973; Grande & Bemis 1998; Gardiner & Schaeffer 1989; Olsen & McCune 1991; Poyato-Ariza & Wenz, 2002; Xu & Gao 2011; Xu et al. 2014, 2015. Gardiner & Schaeffer 1989; Olsen & McCune 1991; Gardiner et al. 1996; Gardiner et al. 2005; Cavin & Suteethorn 2006; Hurley et al. 2007; López-Arbarello 2012; Bermúdez-Rochas & Poyato-Ariza 2015; Poyato-Ariza 2015; Thies & Waschkewitz, 2015; Gibson 2016; Xu & Zhao, 2016; Giles et al., 2017; Cawley & Kriwet 2018; López-Arbarello & Sferco 2018.)

0 absent

1 present

119. Branchiostegal rays - dorsal-most in series

(Lund et al., 1995; Cloutier & Arratia, 2004; Choo, 2011; Giles et al., 2015b; Giles et al., 2017.)

0 of similar depth to adjacent branchiostegal ray

1 deeper than adjacent branchiostegal ray

120. Lateral gulars

(Olsen & McCune 1991; Xu et al., 2014; Giles et al., 2017.)

0 present

1 absent

121. Lateral gulars

(Gardiner & Schaeffer, 1989; Cloutier & Ahlberg, 1996; Taverne, 1997; Lund & Poplin, 1997; Coates, 1999; Schultze & Cumbaa, 2001; Zhu & Schultze, 2001; Cloutier & Arratia, 2004; Friedman & Blom, 2006; Long et al., 2008; Swartz, 2009; Brazeau, 2009; Xu & Gao, 2011; Davis et al., 2012; Zhu et al., 2013; Xu et al., 2014; Giles et al., 2015b,c; Giles et al., 2017. The condition in *Boreosomus* (Nielsen, 1942) is unique: instead of lateral gulars flanking a median gular, there appears to be a second median gular. This may well represent a fusion of the two, longer lateral gulars, is coded as such.)

0 extending most of the length of the lower jaw

1 restricted to the anterior third of the lower jaw (no longer than the width of three branchiostegals)

122. Median gular

(Olsen & McCune 1991; Lund et al., 1995; Cloutier & Ahlberg, 1996; Coates, 1999; Lund, 2000; Schultze & Cumbaa, 2001; Zhu & Schultze, 2001; Zhu et al., 2001: Lund & Poplin, 2002; Zhu & Yu, 2002; Cloutier & Arratia, 2004; Zhu et al., 2006; Friedman, 2007; Zhu et al., 2009; Xu & Gao, 2011; López-Arbarello 2012; Zhu et al., 2013; Bermúdez-Rochas & Poyato-Ariza 2015; Poyato-Ariza 2015; Xu et al., 2014, 2015; Giles et al., 2015b,c; Thies & Waschkewitz, 2015; Xu & Zhao, 2015; Gibson 2016; Giles et al., 2017; López-Arbarello & Sferco 2018. Pearson & Westoll (1979: p. 365) state that a median gular is not known in *Cheirolepis canadensis*. Although a median gular is reconstructed by Cloutier & Arratia

(1996: fig. 7), this bone is not present in any specimen photos and is not mentioned in the text. As such, this taxon is coded as '?'.)

0 absent

1 present

123. Relative length of median gular

(Giles et al., 2015b,c; Giles et al., 2017. The condition in *Boreosomus* (Nielsen, 1942) is unique: instead of lateral gulars flanking a median gular, there appears to be a second median gular. This may well represent a fusion of the two, longer lateral gulars, and is coded as such.)

0 much shorter than jaw length

1 more than half of law length

Braincase

124. Fenestra ventrolateralis

(Schultze & Cumbaa, 2001; Zhu & Schultze, 2001; Zhu et al., 2001; Zhu & Yu, 2002; Zhu et al., 2006; Friedman, 2007; Zhu et al., 2009; Zhu et al., 2013; Giles et al., 2015b; Giles et al., 2017.)

0 absent

1 present

125. Palatal opening surrounded by premaxilla, maxilla, dermopalatine and vomer (choana)

(Zhu & Yu, 2002; Friedman, 2007; Giles et al., 2015b; Giles et al., 2017. Character can only be coded in taxa which possess all of these bones.)

0 absent

1 present

126. Internasal cavity

(Ahlberg & Johanson, 1998; Zhu & Yu, 2002; Zhu & Ahlberg, 2004; Daeschler et al., 2006; Long et al., 2006; Friedman, 2007; Zhu et al., 2009; Zhu et al., 2013; Giles et al., 2015b,c; Giles et al., 2017.)

0 absent

1 present

127. Interorbital septum

(Friedman, 2007; Zhu et al., 2009; Brazeau, 2009; Friedman & Brazeau, 2010; Davis et al., 2012; Zhu et al., 2013; Giles et al., 2015b,c; Giles et al., 2017.)

0 broad

1 narrow

128. Optic foramen

(Giles et al., 2017. Primitively in actinopts, the optic nerve exits the cranial cavity into the orbit through paired foramina approximately halfway up the orbital wall. In many Carboniferous and younger taxa, much of the orbital wall is unossified (the optic fenestra). The optic nerves may exit through openings just posterior to (e.g. *Pteronisculus*) or confluent with (e.g. *Pholidophorus*) the optic fenestra. In polypterids and *Fukangichthys*, the optic nerve exits ventrally through paired foramina that abut the parasphenoid.)

0 dorsally positioned

1 ventrally positioned (i.e. abuts parasphenoid)

129. Pronounced median anterior crista on dorsal surface of braincase

(Giles et al., 2015b,c; Giles et al., 2017. Carboniferous and younger actinopts such as *Lawrenciella* (Hamel & Poplin, 2008) have a median crista anterior to the anterior dorsal fontanelle upon which the skull roof sits.)

0 absent

1 present

130. Expanded anterior dorsal fontanelle

(Giles et al., 2015b,c; Giles et al., 2017. The anterior dorsal fontanelle of many Carboniferous and younger actinopts is greatly expanded, in contrast to the smaller fontanelle of Devonian taxa such as *Mimipiscis* (Gardiner, 1984).)

0 absent

1 present

131. Endoskeletal intracranial joint

(Cloutier & Ahlberg, 1996; Ahlberg & Johanson, 1998; Zhu & Ahlberg, 2004; Zhu et al., 2001; Zhu & Yu, 2002; Daeschler et al., 2006; Long et al., 2006; Friedman, 2007; Brazeau, 2009; Zhu et al., 2009; Friedman & Brazeau, 2010; Davis et al., 2013; Zhu et al., 2013; Giles et al., 2015b,c; Giles et al., 2017.)

0 absent

1 present

132. Eye stalk or unfinished area for similar structure

(Zhu & Schultze, 2001; Zhu et al., 2001; Zhu & Yu, 2002; Zhu et al., 2006; Friedman, 2007; Zhu et al., 2009; Zhu et al., 2013; Giles et al., 2015b,c; Giles et al., 2017. This character is coded as absent for taxa that possess a large interorbital fenestra (e.g. *Pteronisculus*, *Coccocephalichthys*, *Kentuckia deani*), as, if present, the eyestalk area would be visible posterior to the opening for the optic nerve.)

0 absent

1 present

133. Roof of posterior myodome perforated by palatine branch of facial nerve (VII)

(Coates, 1999; Giles et al., 2015b; Giles et al., 2017.)

0 absent

1 present

134. Foramen for abducens nerve (VI) dorsally positioned (level with optic foramen (II))

(Coates, 1999; Giles et al., 2015b; Giles et al., 2017.)

0 absent

1 present

135. Anterodorsal myodome

(Patterson 1973; Olsen & McCune 1991; Coates, 1999; Gardiner et al. 1996; Hurley et al. 2007; Xu & Gao, 2011; Xu et al., 2014, 2015; Giles et al., 2015b; Poyato-Ariza 2015; Xu & Zhao, 2016; Giles et al., 2017.)

0 paired

1 single

2 absent

136. Posterior myodome

(Modified from Patterson 1973; Olsen & McCune 1991; Coates, 1999; Xu et al., 2014. Wiley 1976; Gardiner, 1984; Gardiner & Schaeffer, 1989; Gardiner et al. 1996; Hurley et al. 2007; López-Arbarello 2012; Xu & Gao, 2011; Bermúdez-Rochas & Poyato-Ariza 2015; Xu et al. 2014, 2015; Giles et al., 2015b; Thies & Waschkewitz, 2015; Gibson 2016; Xu & Zhao, 2016; Giles et al., 2017; López-Arbarello & Sferco 2018.)

0 absent

1 paired

2 median

137. Basicranial fenestra

(Ahlberg & Johanson, 1998; Zhu et al., 2001; Zhu & Yu, 2002; Zhu & Ahlberg, 2004; Friedman, 2007; Zhu et al., 2009; Zhu et al., 2013; Giles et al., 2015b,c; Giles et al., 2017.)

0 absent

1 present

138. Spiracle

(Modified from Patterson, 1982; Gardiner, 1984; Gardiner & Schaeffer, 1989; Taverne, 1997; Coates, 1999; Gardiner et al., 2005; Xu & Gao, 2011; Xu et al., 2014; Giles et al., 2015b; Giles et al., 2017; López-Arbarello & Sferco 2018. Taxa that lack a groove on the lateral commissure are coded as inapplicable for this character.)

0 open

1 partial closure or spiracular bar

2 complete enclosure in canal

139. Basipterygoid process

(Gardiner et al., 2005; Xu & Gao, 2011; Xu et al., 2014, 2015; Giles et al., 2015b; Xu & Zhao, 2016; Giles et al., 2017; López-Arbarello & Sferco 2018.)

0 present

1 absent

140. Basipterygoid process with vertically oriented component

(Ahlberg & Johanson, 1998; Zhu & Schultze, 2001; Zhu et al., 2001; Zhu & Yu, 2002; Zhu & Ahlberg, 2004; Zhu et al., 2006; Friedman, 2007; Zhu et al., 2009; Davis et al., 2012; Zhu et al., 2013; Giles et al., 2015b,c; Giles et al., 2017.)

0 absent

1 present

141. Dermal component to basipterygoid process

(Gardiner, 1984; Gardiner & Schaeffer, 1989; Taverne, 1997; Coates, 1999; Giles et al., 2015b; Giles et al., 2017.)

0 absent

1 present

142. Hyoid facet

(Gardiner et al. 2005; Gardiner et al. 1996; Hurley et al. 2007; Xu & Gao 2011; Xu et al. 2015; Giles et al., 2017; López-Arbarello & Sferco 2018.)

0 directed posteroventrally

1 horizontal

143. Fossa bridgei

(Gardiner, 1984; Gardiner & Schaeffer, 1989; Taverne, 1997; Coates, 1999; Xu & Gao, 2011; Xu et al., 2014; Giles et al., 2015b; Giles et al., 2017.)

0 absent

1 present

144. Posttemporal fossae

(Zhu & Yu, 2002; Friedman, 2007.)

0 absent

1 present

145. Vestibular fontanelle

(Friedman, 2007; Brazeau, 2009; Zhu et al., 2009; Friedman & Brazeau, 2010; Davis et al., 2012; Zhu et al., 2013; Brazeau & Friedman, 2014; Giles et al., 2015b,c; Giles et al., 2017.)

0 absent

1 present

146. Ventral cranial fissure and vestibular fontanelle

(Coates, 1999; Giles et al., 2015b; Giles et al., 2017. We follow Coates (1999) in coding Howqualepis as '0' on the basis of Long 1988 fig. 16 and AMF65495 (pers. obs. S.G.), rather than the braincase reconstruction (Long, 1988: fig. 18.)

0 separated by bridge of bone

1 confluent

147. Accessory fenestration in otic capsule

(Friedman, 2007; Zhu et al., 2009; Giles et al., 2015b; Giles et al., 2017.)

0 absent

1 present

148. Otoccipital fissure

(Friedman, 2007; Brazeau, 2009; Davis et al., 2012; Zhu et al., 2013; Giles et al., 2015b,c; Giles et al., 2017.)

0 absent

1 present

149. Median projection overhanging posterior part of posterior dorsal fontanelle

(Giles et al., 2015b; Giles et al., 2017. Variable in *Boreosomus*: the posterior dorsal fontanelle is closed in the specimen figured in Nielsen (1942: plate 25F), but developed in the specimen figured in plate 28. This taxon is coded '0/1' to reflect this polymorphism.)

0 absent

1 present

150. Median projection overhanging anterior part of posterior dorsal fontanelle

(Giles et al., 2015b; Giles et al., 2017. This projection is somewhat reduced in *Pteronisculus* (Nielsen, 1942), but is coded '1' here. Variable in *Boreosomus*: the posterior dorsal fontanelle is closed in the specimen figured in Nielsen 1942 plate 25F, but developed in the specimen figured in plate 28. This taxon is coded '0/1' to reflect this polymorphism. *Cheirolepis trailli* is coded '0' (2015a).)

0 absent

1 present

151. Dorsal aorta

(Coates & Sequeira, 1998; Coates & Sequeira, 2001a, b; Coates, 1999; Friedman, 2007; Zhu et al., 2009; Friedman & Brazeau, 2010; Zhu et al., 2013; Giles et al., 2015b,c; Giles et al., 2017; López-Arbarello & Sferco 2018. This character is coded as inapplicable in taxa that lack a canal for the dorsal aorta. *Cheirolepis trailli* is coded '0' (Giles et al., 2015a). The aortic canal of *Moythomasia* is not figured by Gardiner (1984), but a clear posterior notch in the aortic canal can be seen in Long & Trinajstic (2010:fig 5b). The neurocranium of *Gogosardina* is not yet described, but this character can be coded on the basis of Choo et al. (2009: fig. 9).)

0 open in groove

1 canal notched posteriorly

2 completely enclosed in canal

152. Dorsal aorta pierced by canal/s for exit of eff.a.2

(Giles et al., 2015b; Giles et al., 2017. In *Mimipiscis bartrami* and *M. toombsi*, a groove for one of the efferent branchial arteries branches off from the lateral dorsal aorta immediately before the articular area for the first infrapharyngobranchial. However, it is uncertain which, so both taxa coded as '?' for these characters. The neurocranium of *Gogosardina* is not yet described, but this character can be coded on the basis of Choo et al. (2009: fig. 9).)

0 absent

1 present

153. Dorsal aorta pierced by canal/s for exit of eff.a.1

(Giles et al., 2015b; Giles et al., 2017. In *Mimipiscis bartrami* and *M. toombsi*, a groove for one of the efferent branchial arteries branches off from the lateral dorsal aorta immediately before the articular area for the first infrapharyngobranchial. However, it is uncertain which, so both taxa coded as '?' for these characters. The neurocranium of *Gogosardina* is not yet described, but this character can be coded on the basis of Choo et al. (2009: fig. 9).)

0 absent

1 present

154. Bifurcation of dorsal aorta

(Coates & Sequeira, 1998; Coates & Sequeira, 2001a, b; Coates, 1999; Friedman, 2007; Zhu et al., 2009; Friedman & Brazeau, 2010; Zhu et al., 2013; Giles et al., 2015b,c; Giles et al., 2017.)

0 posterior to occiput

1 anterior to occiput

155. Birfurcation of dorsal aorta into lateral dorsal aortae

(Coates, 1999; Giles et al., 2015b; Giles et al., 2017. This character is coded as inapplicable in taxa that lack a canal for the dorsal aorta. In *Mimipiscis toombsi*, the bifurcation point of the dorsal aorta can be open (Giles & Friedman, 2014: fig. 2) or closed (Gardiner 1984: fig. 15). This taxon is coded '0/1' to reflect this polymorphism. The aortic canal of *Moythomasia* is not figured by Gardiner (1984), but the bifucation into the lateral dorsal aortae can be seen in Long & Trinajstic (2010:fig 5b).)

0 open

1 enclosed in canal

156. Braincase ossifications differentiated

(Giles et al., 2017.)

0 absent

1 present

157. Basisphenoid

(Wiley 1976; López-Arbarello 2012; Bermúdez-Rochas & Poyato-Ariza 2015; Thies & Waschkewitz, 2015; Gibson 2016; Giles et al., 2017; López-Arbarello & Sferco 2018.)

0 present

1 absent or very reduced

158. Opisthotic-pterotic relationship

(Gardiner et al. 1996; Hurley et al. 2007; Giles et al., 2017. This character can only be coded when separate braincase ossifications can be identified.)

0 opisthotic larger than subotic

1 opisthotic and pterotic equal in size

159. Epioccipital

(Hurley et al. 2007; Giles et al., 2017. This character can only be coded when separate braincase ossifications can be identified.)

0 present

1 absent

160. Forward extension of the exoccipital around the vagus nerve

(Olsen & McCune 1991; Gardiner et al. 1996; Cavin & Suteethorn 2006; Hurley et al. 2007; López-Arbarello 2012; Bermúdez-Rochas & Poyato-Ariza 2015; Thies & Waschkewitz, 2015; Gibson 2016; Giles et al. 2017; López-Arbarello & Sferco 2018. This character can only be coded when separate braincase ossifications can be identified.)

0 absent

1 present

161. Spenotic with small dermal component

(Grande 2010; López-Arbarello 2012; Xu & Wu 2012; Xu et al. 2014, 2015; Arratia 2013; Bermúdez-Rochas & Poyato-Ariza 2015; Poyato-Ariza 2015; Thies & Waschkewitz, 2015; Gibson 2016; Xu & Zhao, 2016; Giles et al. 2017; López-Arbarello & Sferco 2018. This character can only be coded when separate braincase ossifications can be identified.)

0 absent

1 present

162. Pterotic

(Gardiner et al. 1996; Grande & Bemis, 1998; Hurley et al. 2007; Xu et al. 2014; Poyato-Ariza 2015; Xu & Shen, 2015; Xu & Zhao, 2016; Giles et al. 2017; López-Arbarello & Sferco 2018. This character can only be coded when separate braincase ossifications can be identified.)

0 present

1 absent

163. Opisthotic bone

(Wiley 1976; Olsen & McCune 1991; Grande & Bemis 1998; Cavin & Suteethorn 2006; Hurley et al. 2007; Grande 2010; López-Arbarello 2012; Bermúdez-Rochas & Poyato-Ariza 2015; Poyato-Ariza 2015; Thies & Waschkewitz, 2015; Xu et al., 2014, 2015; Gibson 2016; Xu & Zhao, 2016; Giles et al. 2017; López-Arbarello & Sferco 2018. This character can only be coded when separate braincase ossifications can be identified.)

0 present

1 absent

164. Intercalar

(Olsen & McCune 1991; Olsen 1994; Gardiner et al. 1996; López-Arbarello 2012; Xu et al. 2014; Poyato-Ariza 2015; Xu & Shen, 2015; Thies & Waschkewitz, 2015; Gibson 2016; Xu & Zhao, 2016; Giles et al. 2017; López-Arbarello & Sferco 2018. This character can only be coded when separate braincase ossifications can be identified.)

0 present

1 absent

165. Supraoccipital bone

(Grande 2010; Poyato-Ariza 2015; Xu et al. 2014, 2015; Xu & Zhao, 2016; Giles et al. 2017; López-Arbarello & Sferco 2018. This character can only be coded when separate braincase ossifications can be identified.)

0 absent

1 present

166. Membranous outgrowth of intercalar

(Patterson 1973; Olsen & McCune 1991; Gardiner et al. 1996; Hurley et al. 2007; Giles et al. 2017; López-Arbarello & Sferco 2018. This character can only be coded when separate braincase ossifications can be identified.)

0 absent

1 present

167. Post-temporal fossa

(Patterson 1973; Gardiner, 1984; Gardiner & Schaeffer, 1989; Olsen & McCune 1991; Coates, 1999; Hurley et al. 2007; López-Arbarello 2012; Xu & Gao, 2011; Xu et al. 2014, 2015; Bermúdez-Rochas & Poyato-Ariza 2015; Giles et al., 2015b; Poyato-Ariza 2015; Thies & Waschkewitz, 2015; Gibson 2016; Xu & Zhao, 2016; Giles et al. 2017.)

0 absent

1 present

168. Sub-temporal fossa

(Gardiner, 1984; Gardiner & Schaeffer, 1989; Gardiner et al. 1996; Hurley et al. 2007; Xu & Gao, 2011; Xu et al. 2014, 2015; Xu & Zhao, 2016.)

0 absent

1 present

169. Dilatator fossa

(Gardiner, 1984; Gardiner & Schaeffer, 1989; Coates, 1999; Gardiner et al. 1996; Hurley et al. 2007; Xu & Gao, 2011; Xu et al. 2014, 2015; Xu & Zhao, 2016; Giles et al. 2017.)

0 absent

1 present

170. Parasphenoid

(Gardiner, 1984; Brazeau, 2009; Davis et al., 2012; Zhu et al., 2013; Giles et al., 2015b,c; Giles et al. 2017.)

0 absent

1 present

171. Parasphenoid

(Coates, 1999; Zhu & Yu, 2002; Gardiner et al., 2005; Friedman, 2007, Xu & Gao, 2011; Xu et al., 2014, 2015; Giles et al., 2015b; Xu & Zhao, 2016; Giles et al. 2017; López-Arbarello & Sferco 2018.)

0 terminates at/anterior to ventral otic fissure

1 extends across ventral otic fissure

2 extends to basioccipital

172. Ascending process of the parasphenoid

(Patterson, 1982; Coates, 1999; Dietze, 2000; Schultze & Cumbaa, 2001; Zhu & Schultze, 2001; Cloutier & Arratia, 2004; Gardiner et al., 2005; Friedman & Blom, 2006; Zhu et al., 2006; Zhu et al., 2009; Choo, 2011; Xu & Gao, 2011; Zhu et al., 2013; Xu et al., 2014; Giles et al., 2015b,c; Giles et al. 2017; López-Arbarello & Sferco 2018.)

0 absent

1 present

173. Parasphenoid with multifid anterior margin

(Friedman & Blom, 2006; Friedman, 2007; Zhu et al., 2009; Choo, 2011; Zhu et al., 2013; Giles et al., 2015b,c; Giles et al. 2017.)

0 absent

1 present

174. Buccohypophyseal canal pierces parasphenoid

(Giles et al., 2015b,c; Giles et al. 2017. The buccohypophyseal canal typically enters the dorsal surface of the parasphenoid, but whether it exits via the ventral surface is more variable, and this distribution is captured by this character.)

0 present

1 absent

175. Parasphenoid teeth

(Arratia 2013; Giles et al. 2017; López-Arbarello & Sferco 2018.)

0 small

1 large

2 absent

176. Parasphenoid pierced by internal carotid artery

(Gardiner et al. 1996; Hurley et al. 2007; Xu & Wu, 2012; Xu et al., 2015; Xu & Zhao, 2016; Giles et al. 2017.)

0 absent

1 present

177. Parasphenoid pierced by efferent pseudobranchial artery

(Gardiner et al. 1996; Hurley et al. 2007; Xu & Wu, 2012; Xu et al., 2015; Xu & Zhao, 2016; Giles et al. 2017.)

0 absent

1 present

178. Aortic notch in parasphenoid

(Modified from Gardiner et al. 2005; Giles et al. 2017.)

0 absent

1 present

179. Parabasal canal

(Xu and Gao 2011; Xu et al. 2014; Giles et al. 2017.)

0 present

1 absent

Endocast

180. Anterolaterally divergent olfactory tracts

(Coates, 1999; Giles & Friedman, 2014; Giles et al., 2015b; Giles et al. 2017.)

0 absent

1 present

181. Elongate olfactory tract(s)

(Brazeau, 2009; Friedman & Brazeau 2010; Davis et al., 2012; Zhu et al., 2013; Brazeau & Friedman, 2014; Giles & Friedman, 2014; Giles et al., 2015b,c; Giles et al. 2017. The olfactory tracts of *Osorioichthys* are elongate (pers. obs. unpubl. scan data S.G.).)

0 absent

1 present

182. Olfactory nerves carried in a single tract

(Coates, 1999; Giles & Friedman, 2014; Giles et al., 2015b; Giles et al. 2017. The olfactory nerves are carried in separate tracts in *Osorioichthys* (pers. obs. unpubl. scan data S.G.).)

0 present

1 absent

183. Hypophyseal chamber

(Coates, 1999; Xu & Gao, 2011; Xu et al., 2014; Giles et al., 2015b; Giles et al. 2017.)

0 projects posteroventrally

1 projects ventrally or anteroventrally

184. Optic lobes

(Giles & Friedman, 2014; Giles et al., 2015b; Giles et al. 2017.)

0 narrower than cerebellum

1 same width or wider than cerebellum

185. Optic lobes

(Coates 1999; Hurley et al. 2007; Xu et al. 2014; Giles et al. 2017.)

0 smaller than telencephalon

1 larger than telencephalon

186. Optic tectum divided into bilateral halves

(Coates, 1999; Giles et al., 2015b; Giles et al. 2017.)

0 absent

1 present

187. Cerebellar corpus

(Giles et al., 2015b,c; Giles et al. 2017. The region posterior to the cerebellar auricles in *Lawrenciella* was considered to be the area octavolateralis by Hamel & Poplin (2005). However, we interpret it as the corpus cerebellum, and this taxon is coded '1'.)

0 absent

1 present

188. Cerebellar corpus

(Coates 1999; Hurley et al. 2007; Xu & Gao 2011; Xu et al. 2014; Giles et al. 2017.)

0 divided bilaterally

1 undivided

189. Position of cerebellar corpus

(Coates 1999; Hurley et al. 2007; Xu & Gao 2011; Xu et al. 2014; Giles et al. 2017.)

0 enters fourth ventricle

1 arches above fourth ventricle

190. Cerebellar corpus with median anteriorly projecting portion

(Coates 1999; Hurley et al. 2007; Xu & Gao 2011; Xu et al. 2014; Giles et al. 2017.)

0 absent

1 present

191. Horizontal semicircular canal

(Davis et al., 2012; Zhu et al., 2013; Giles & Friedman, 2014; Giles et al., 2015b,c; Giles et al. 2017.)

0 joins vestibular region dorsal to ampulla for the posterior semicircular canal

1 joins vestibular region level with ampulla for the posterior semicircular canal

192. Junction between ampulla of posterior semicircular canal and cranial cavity

(Giles et al., 2015b,c; Giles et al. 2017. In certain stem actinopterygians, such as *Mimipiscis* (Giles and Friedman, 2014), a short length of canal lies between the posterior ampulla and the remainder of the labyrinth.)

0 separated by short length of canal

1 confluent

193. Crus commune of anterior and posterior semicircular canal

(Giles & Friedman, 2014; Giles et al., 2015b; Giles et al. 2017.)

0 dorsal to endocranial roof

1 ventral to endocranial roof

194. Lateral cranial canal

(Gardiner, 1984; Gardiner & Schaeffer, 1989; Coates, 1999; Cloutier & Arratia, 2004; Gardiner et al., 2005; Zhu et al., 2006; Zhu et al., 2009; Zhu et al., 2013; Giles & Friedman,

2014; Xu et al., 2014; Giles et al., 2015b,c; Giles et al. 2017. The presence of a lateral cranial canal in *Ligulalepis* and *Psarolepis* is uncertain, but its presence in *Meemannia* is confirmed following Lu et al. (2016). *Erpetoichthys* is conservatively coded as '?'.)

0 absent

1 present

195. Lateral cranial canal connects to cranial cavity anteriorly

(Giles et al. 2017.)

0 absent

1 present

Scales and histology

196. Enameloid on dermal bones and scales

(Giles et al., 2015b,c, 2017:Characters 147-150 form part of an atomisation of the compound characters 'ganoine' (typically defined as a single or multilayer enamel covering) and 'cosmine' (typically defined as a single layer of enamel with a well-defined pore canal network) (e.g. Cloutier & Ahlberg, 1996; Ahlberg & Johanson, 1998; Zhu & Ahlberg, 2004; Schultze & Cumbaa, 2001; Zhu & Schultze, 2001; Zhu et al., 2001; Zhu & Yu, 2002; Daeschler et al., 2006; Long et al., 2006; Zhu et al., 2006; Zhu et al., 2009; Davis et al., 2012; Zhu et al., 2013). A similar approach to atomization was taken by Friedman (2007), Brazeau & Friedman (2010) and Giles et al. (2015b). As detailed histological investigations have not been carried out for the majority of early actinopterygians (rather, they have simply been described as being covered in/bearing ridges of ganoine), many of these characters cannot be coded for a number of taxa.)

0 absent

1 present

197. Extensive pore-canal network

(See notes above for c. 147.)

0 absent

1 present

198. Enamel

(See notes above for c. 147.)

0 single-layered

1 multi-layered

199. Enamel layers

(See notes above for c. 147.)

0 applied directly to one another

1 separated by layers of dentine

200. Scales on body

(Giles et al. 2017.)

0 present

1 absent

201. Scales

(Cloutier & Arratia, 2004; Friedman & Blom, 2006; Long et al., 2008; Swartz, 2009; Zhu et al., 2009; Choo, 2011; Giles et al., 2015b; Giles et al. 2017.)

0 micromeric

1 macromeric

202. Scales with ‘peg and socket articulation'

(Maisey, 1986; Gardiner & Schaeffer, 1989; Cloutier & Ahlberg, 1996; Coates, 1999; Dietze, 2000; Poplin & Lund, 2000; Schultze & Cumbaa, 2001; Cloutier & Arratia, 2004; Friedman & Blom, 2006; Friedman, 2007; Long et al., 2008; Brazeau, 2009; Swartz, 2009; Zhu et al., 2009; Friedman & Brazeau, 2010; López-Arbarello 2012; Xu & Gao, 2011; Choo, 2011; Davis et al., 2012; Zhu et al., 2013; Xu et al., 2014; Giles et al., 2015b,c; Xu & Zhao, 2016; Giles et al. 2017; López-Arbarello & Sferco 2018. This character is coded only for taxa that possess rhombic scales.)

0 absent

1 present

203. Peg on rhomboid scale

(Patterson, 1982; Cloutier & Ahlberg, 1996; Dietze, 2000; Schultze & Cumbaa, 2001; Zhu & Schultze, 2001; Zhu et al., 2001: Zhu & Yu, 2002; Cloutier & Arratia, 2004; Friedman & Blom, 2006; Zhu et al., 2006; Friedman, 2007; Zhu et al., 2009; Giles et al. 2017. Although peg-and-socket articulation of is present between the scales of *Limnomis* (Daeschler, 2000), the nature of the peg is not described. As such, this taxon is conservatively coded '?'.)

0 narrow

1 broad

204. Anterodorsal process on scale

(Patterson, 1982; Gardiner, 1984; Gardiner & Schaeffer, 1989; Schultze & Cumbaa, 2001; Zhu & Schultze, 2001; Zhu et al., 2001; Zhu & Yu, 2002; Cloutier & Arratia, 2004; Friedman & Blom, 2006; Zhu et al., 2006; Friedman, 2007; Long et al., 2008; Swartz, 2009; Zhu et al., 2009; Choo, 2011; Zhu et al., 2013; Giles et al., 2015b,c; Giles et al. 2017.)

0 absent

1 present

205. Scales with well-developed pores on surface

(Friedman & Blom 2006; Long et al., 2008; Swartz, 2009; Choo, 2011; Xu et al., 2014; Giles et al., 2015b; Giles et al. 2017. Scales from the posterior half of the flank in *Wendichthys* bear pores on the enamel surface, whereas those from the anterior part of the flank lack these pores (Lund & Poplin, 1997: fig. 6). This taxon is scored '1'.)

0 absent

1 present

206. Small scales below dorsal fin

(Giles et al. 2017.)

0 absent

1 present

207. Lepidotrichia

(Friedman, 2007; Brazeau, 2009; Zhu et al., 2009; Friedman & Brazeau, 2010; Davis et al., 2012; Zhu et al., 2013; Brazeau & Friedman, 2014; Giles et al., 2015b,c; Giles et al. 2017.)

0 absent

1 present

208. Fringing fulcra

(Patterson, 1982; Gardiner & Schaeffer, 1989; Grande & Bemis 1998; Coates, 1999; Dietze, 2000; Schultze & Cumbaa, 2001; Poyato-Ariza & Wenz, 2002; Cloutier & Arratia, 2004; Friedman & Blom, 2006; Friedman, 2007; Long et al., 2008; Swartz, 2009; Zhu et al., 2009; Choo, 2011; Xu & Gao, 2011; López-Arbarello 2012; Zhu et al., 2013; Xu et al., 2014, 2015; Bermúdez-Rochas & Poyato-Ariza 2015; Poyato-Ariza 2015; Giles et al., 2015b; Thies & Waschkewitz, 2015; Gibson 2016; Xu & Zhao, 2016; Giles et al. 2017; Cawley & Kriwet 2018; López-Arbarello & Sferco 2018.)

0 absent

1 present

Hyoid arch

209. Double headed hyomandibular

(Cloutier & Ahlberg, 1996; Zhu & Schultze, 2001; Schultze & Cumbaa, 2001; Zhu et al., 2001; Zhu & Yu, 2002; Zhu et al., 2006; Friedman, 2007; Zhu et al., 2009; Friedman & Brazeau, 2010; Zhu et al., 2013; Giles et al., 2015b,c; Giles et al. 2017; López-Arbarello & Sferco 2018.)

0 absent

1 present

210. Perforate hyomandibula

(Friedman, 2007; Zhu et al., 2009; Friedman & Brazeau, 2010; Xu & Gao, 2011; Zhu et al., 2013; Brazeau & Friedman, 2014; Xu et al., 2014, 2015; Giles et al., 2015b; Xu & Zhao, 2016; Giles et al. 2017. Although Long (1988: p.24) mentions the presence of a depression for the hyomandibular nerve in *Howqualepis*, it is unclear whether this perforated the hyomandibula. This taxon is conservatively coded as '?'.)

0 absent

1 present

211. Opercular process

(Gardiner & Schaeffer, 1989 Poyato-Ariza & Wenz, 2002;; Giles et al., 2015b; Giles et al. 2017; Cawley & Kriwet 2018; López-Arbarello & Sferco 2018.) 0 absent

1 present

212. Ceratohyal

(Gardiner et al., 2005; Xu & Gao, 2011; Giles et al., 2015b; Giles et al. 2017; Xu et al., 2014.)

0 single ossification

1 two ossifications

213. Anterior ossification of ceratohyal

(Revised from Coates, 1999; Giles et al., 2015b; Giles et al. 2017; López-Arbarello & Sferco 2018.The character captures whether the ceratohyal (or the anterior ossification if an anterior and posterior ceratohyal are present) is medially constricted (hourglass-shaped) or plate-like in lateral view.)

0 no medial constriction

1 medial constriction (hourglass-shaped)

214. Anterior ceratohyal

(Coates, 1999; Giles et al., 2015b; Giles et al. 2017. The groove for the afferent hyoidean artery in the ceratohyal of *Gogosardina* is visible in Choo 2009 (fig 6).)

0 no groove

1 groove for afferent hyoidean artery

215. Interhyal

(Davis et al., 2012; Zhu et al., 2013; Giles et al., 2015b,c; Giles et al. 2017.)

0 absent

1 present

216. Symplectic

(Gardiner 1984; Gardiner & Schaeffer 1989; Coates 1999; Hurley et al. 2007; Poyato-Ariza 2015; Xu & Zhao, 2016; Giles et al. 2017; López-Arbarello & Sferco 2018. The general actinopterygian condition of the hyoid arch seems to comprise four ossifications: hyomandibula, ceratohyal (which may be one or two bones), hypohyal, and an intermediate bone between the hyomandibula and ceratohyal termed, variably, the interhyal or symplectic. In some actinopts (e.g. *Amia*, *Lepisosteus*, *Hiodon*, *Dorsetichthys*, *Macrosemionotus*, etc.), a second intermediate cartilage is present. The history attached to naming these terms is very complex (see Paterson 1973, Patterson 1982, Véran 1988, Gardiner et al. 1996, etc.), and we have tried here to apply a simple, consistent approach. The ossification that forms an intermediary between the hyomandibula and ceratohyal is termed the interhyal. This is primitively present (and in contact with the quadrate), and may be very reduced (e.g.

Watsonulus, *Elops*), or entirely cartilaginous (e.g. *Amia*, *Lepisosteus*) in more derived actinopts. The ossification that contacts the hyomandibula (and typically the quadrate), but does not articulate with the ceratohyal, is termed the symplectic. This element may brace the quadrate, and in *Watsonulus*, *Caturus* and *Amia* additionally articulates with the lower jaw. We follow Grande (2010) in identifying the 'symplectic' of *Acipenser* as the posterior ceratohyal. Véran (1988) identified a second intermediate ossification in the hyoid arch in a number of 'palaeoniscids', which she termed a symplectic. This identification has been disputed on the basis of position (e.g. Gardiner et al. 1996). We have seen no evidence (either through visual examination or CT scanning) for a second intermediate hyoid ossification in any specimens of *Boreosomus* or *Pteronisculus*. From examination of *Coccocephalichthys*, we identify the 'symplectic' of Véran to be the interhyal and the 'interhyal' of Véran to correspond to the articular. This casts doubt on the presence of a second element, and we have therefore coded *Boreosomus*, *Pteronisculus* and *Coccocephalicthys* as '0'.)

0 absent

1 present

217. Symplectic shape c68

(Giles et al. 2017; López-Arbarello & Sferco 2018.)

0 tube/splint like

1 hatchet

2 l-shaped

218. Hypohyal

(Friedman & Brazeau, 2010; Brazeau & Friedman, 2014; Giles et al., 2015b,c; Giles et al. 2017.)

0 absent

1 present

219. Basihyal

(Davis et al., 2012; Zhu et al., 2013; Giles et al., 2015b,c; Giles et al. 2017; López-Arbarello & Sferco 2018.)

0 absent

1 present

Gill skeleton

220. Gill arches

(Giles et al., 2015b,c; Giles et al. 2017.)

0 largely restricted to area under braincase

1 extend far posterior to braincase

221. Number of ceratobranchials

(Giles et al. 2017.)

0 five

1 four

222. Number of hypobranchials

(Grande, 2010; Xu & Wu, 2012; Xu et al., 2014, 2015; Xu & Zhao, 2016; Giles et al. 2017; López-Arbarello & Sferco 2018.)

0 three

1 four

223. Uncinate processes on epibranchials

(Coates, 1999; Xu & Gao, 2011; Xu et al., 2014, 2015; Poyato-Ariza 2015; Xu & Zhao, 2016; Giles et al. 2017; López-Arbarello & Sferco 2018. An uncinate process is a dorsally-directed extension on the epibranchial that articulates with the pharyngobranchial skeleton.)

0 absent

1 present

224. Endoskeletal urohyal

(Friedman, 2007; Friedman & Brazeau, 2010; Giles et al., 2015b,c; Poyato-Ariza 2015; Giles et al. 2017; López-Arbarello & Sferco 2018.)

0 absent

1 present

225. Urohyal formed as a tendon bone of the sternohyoideus muscle

(Arratia 2013; Giles et al. 2017.)

0 absent

1 present

226. Presupracleithrum

(Patterson, 1982; Gardiner, 1984; Gardiner & Schaeffer, 1989; Taverne, 1997; Lund, 2000; Schultze & Cumbaa, 2001; Zhu & Schultze, 2001; Zhu et al., 2001; Lund & Poplin, 2002; Zhu & Yu, 2002; Cloutier & Arratia, 2004; Gardiner et al., 2005; Friedman & Blom, 2006; Zhu et al., 2006; Friedman, 2007; Long et al., 2008; Swartz, 2009; Zhu et al., 2009; Choo,

2011; Xu & Gao, 2011; Zhu et al., 2013; Xu et al., 2014; Giles et al., 2015b; Giles et al. 2017; López-Arbarello & Sferco 2018. An elongate bone termed the 'anocleithrum' is variably present in *Wendichthys* (Lund & Poplin, 1997) in the position occupied by the presupracleithrum in other taxa. We regard this as a positional homologue, and code the taxon '0/1' to reflect this polymorphism. Coded as '?' in *C. trailli* following arguments in Friedman & Blom (2006).)

0 absent

1 present

Dermal shoulder girdle

227. Presupracleithrum

(Xu et al. 2014; Giles et al. 2017.)

0 single

1 multiple

228. Dorsal margin of cleithrum

(Cloutier & Ahlberg, 1996; Schultze & Cumbaa, 2001; Zhu & Schultze, 2001; Zhu et al., 2001; Zhu & Yu, 2002; Cloutier & Arratia, 2004; Zhu et al., 2006; Friedman, 2007; Zhu et al., 2009; Giles et al., 2015b,c; Giles et al. 2017.)

0 pointed

1 broad and rounded

229. Medial wing on cleithrum

(Cavin & Suteethorn 2006; Poyato-Ariza 2015; Giles et al. 2017; López-Arbarello & Sferco 2018.)

0 absent

1 present

230. Anocleithrum

(Gardiner & Schaeffer, 1989; Lund et al., 1995; Cloutier & Ahlberg, 1996; Dietze, 2000; Poplin & Lund, 2000; Schultze & Cumbaa, 2001; Zhu & Schultze, 2001; Zhu et al., 2001; Zhu & Yu, 2002; Cloutier & Arratia, 2004; Zhu et al., 2006; Friedman, 2007; Zhu et al., 2009; Zhu et al., 2013; Giles et al., 2015b; Giles et al. 2017; López-Arbarello & Sferco 2018.)

0 bone developed as postcleithrum

1 bone developed as anocleithrum sensu stricto

2 bone absent

231. Clavicle

(Patterson 1973; Olsen & McCune 1991; Coates 1999; Xu & Gao 2011; Xu et al. 2014; Poyato-Ariza 2015; Xu & Zhao, 2016; Giles et al. 2017; López-Arbarello & Sferco 2018.)

0 present as a broad plate

1 much reduced or absent

232. Serrated organ

(Arratia 2013; Giles et al. 2017; López-Arbarello & Sferco 2018. The serrated organ (or appendage) is a small, elongate element, typically ornamented with serrated ridges, present near the anterior margin of the cleithrum.)

0 absent

1 present

233. Interclavicle

(Cloutier & Arratia, 2004; Giles et al. 2017; Xu et al., 2014.)

0 present

1 absent

Paired fins

234. Triradiate scapulocoracoid

(Olsen & McCune 1991; Zhu & Schultze, 2001; Zhu et al., 2001; Zhu & Yu, 2002; Zhu et al., 2006; Friedman, 2007; Zhu et al., 2009; Xu & Gao, 2011; Zhu et al., 2013; Xu et al., 2014; Giles et al., 2015b; Giles et al. 2017.)

0 absent

1 present

235. Perforate propterygium

(Patterson, 1982; Gardiner, 1984; Gardiner & Schaeffer, 1989; Rosen, 1989; Taverne, 1997; Coates, 1999; Zhu & Schultze, 2001; Zhu et al., 2001; Zhu & Yu, 2002; Zhu et al., 2006; Brazeau, 2009; Zhu et al., 2009; Friedman & Brazeau, 2010; Xu & Gao, 2011; Davis et al., 2012; Zhu et al., 2013; Xu et al., 2014; Giles et al., 2015b,c.)

0 absent

1 present

236. Anterior rays embrace propterygium

(Patterson, 1982; Gardiner, 1984; Gardiner & Schaeffer, 1989; Taverne, 1997; Coates, 1999; Schultze & Cumbaa, 2001; Zhu & Schultze, 2001; Friedman & Blom, 2006; Long et al., 2008; Swartz, 2009; Choo, 2011; Xu & Gao, 2011; Giles et al., 2015b; Giles et al. 2017.)

0 absent

1 present

2 fused

237. Propterygium fused to first ray

(Giles et al. 2017; López-Arbarello & Sferco 2018.)

0 absent

1 present

238. Pectoral fin endoskeleton

(Taverne, 1997; Coates, 1999; Friedman & Blom, 2006; Long et al., 2008; Swartz, 2009; Xu & Gao, 2011; Xu et al., 2014; Giles et al., 2015b; Giles et al. 2017.)

0 extends far beyond body wall (fins lobate)

1 barely extends beyond body wall (fins not lobate)

239. Pectoral fin radials

(Zhu & Yu, 2002; Friedman, 2007; Giles et al., 2015b; Giles et al. 2017. Two series of pectoral fin radials are described (but not figured) for *Cheirolepis candensis* (Arratia & Cloutier, 2004). Although we consider this arrangement to be unlikely, for now this taxon is coded '1'.)

0 unjointed

1 jointed

240. Fin articulation

(Zhu & Schultze, 2001; Zhu et al., 2001; Zhu & Yu, 2002; Zhu et al., 2006; Friedman, 2007; Zhu et al., 2009; Friedman & Brazeau, 2010; Zhu et al., 2013; Giles et al., 2015b,c; Giles et al. 2017.)

0 monobasal

1 polybasal

241. Pectoral fin-ray segmentation

(Coates, 1999; Friedman & Blom, 2006; Long et al., 2008; Choo, 2011; Xu & Gao, 2011; Xu et al., 2014; Giles et al., 2015b; Giles et al. 2017.)

0 roughly even segmentation to fin base

1 proximal segments elongate with terminal segmentation

2 no significant segmentation on pectoral fin

3 terminal segments elongate with proximal segmentation

242. Pectoral fin

(Giles et al., 2015b; Giles et al. 2017.)

0 leaf-like

1 not leaf-like

243. Paired fin spines

(Zhu et al., 2001; Zhu & Yu, 2002; Friedman, 2007; Zhu et al., 2009; Brazeau, 2009; Davis et al., 2012; Zhu et al., 2013; Giles et al., 2015b,c; Giles et al. 2017.)

0 absent

1 present

244. Pelvic fins

(Friedman & Blom, 2006; Friedman, 2007; Brazeau, 2009; Choo, 2011; Davis et al., 2012; Zhu et al., 2013; Brazeau & Friedman, 2014; Giles et al., 2015b,c; Giles et al. 2017.)

0 absent

1 present

245. Pelvic fin insertion

(Gardiner & Schaeffer, 1989; Coates, 1998; Coates, 1999; Lund, 2000; Schultze & Cumbaa, 2001; Cloutier & Arratia, 2004; Friedman & Blom, 2006; Zhu et al., 2006; Long et al., 2008; Swartz, 2009; Zhu et al., 2009; Choo, 2011; Xu et al., 2014; Giles et al., 2015b; Giles et al. 2017.)

0 shorter than fin depth (short based)

1 longer than fin depth (long based)

Median fins

246. Basal scutes on fins

(Friedman, 2007; Zhu & Yu, 2002; López-Arbarello 2012; Bermúdez-Rochas & Poyato-Ariza 2015; Giles et al., 2015b; Thies & Waschkewitz, 2015; Gibson 2016; Giles et al. 2017; López-Arbarello & Sferco 2018.)

0 absent

1 present

247. Dorsal scutes anterior to dorsal fin

(Olsen & McCune 1991; Lund, 2000; Poplin & Lund, 2000; Poyato-Ariza & Wenz, 2002; Cloutier & Arratia, 2004; Friedman & Blom, 2006; Long et al., 2008; Swartz, 2009; Choo, 2011; Giles et al., 2015b; Thies & Waschkewitz, 2015; Gibson 2016; Giles et al. 2017; Cawley & Kriwet 2018; López-Arbarello & Sferco 2018.)

0 absent

1 few limited to region immediately anterior to fin (basal fulcra only)

2 many, extending to posterior of skull roof (complete set of dorsal ridge scales)

248. Ventral scutes between hypochordal lobe of caudal fin and anal fin

(Patterson, 1982; Taverne, 1997; Friedman & Blom, 2006; Long et al., 2008; Choo, 2011; Giles et al., 2015b; Giles et al. 2017; López-Arbarello & Sferco 2018.)

0 absent

1 present

249. Ventral scutes anterior to anal fin

(Cloutier & Arratia, 2004; Friedman & Blom, 2006; Long et al., 2008; Swartz, 2009; Choo, 2011; Giles et al., 2015b; Giles et al. 2017.)

0 absent

1 present

250. Dorsal fin(s)

(Gardiner & Schaeffer, 1989; Schultze & Cumbaa, 2001; Zhu & Schultze, 2001; Zhu et al., 2001; Zhu & Yu, 2002; Cloutier & Arratia, 2004; Friedman & Blom, 2006; Zhu et al., 2006;

Friedman, 2007; Long et al., 2008; Brazeau, 2009; Swartz, 2009; Zhu et al., 2009; Choo, 2011; Davis et al., 2012; Zhu et al., 2013; Giles et al., 2015b,c; Giles et al. 2017.)

0 two

1 one

251. Relative positions of anal and (second) dorsal fin

(Poplin & Lund, 2000; Cloutier & Arratia, 2004; Friedman & Blom, 2006; Long et al., 2008; Swartz, 2009; Choo, 2011; López-Arbarello 2012; Bermúdez-Rochas & Poyato-Ariza 2015; Giles et al., 2015b; Thies & Waschkewitz, 2015; Gibson 2016; Giles et al. 2017; López-Arbarello & Sferco 2018.)

0 anal shifted anteriorly relative to dorsal

1 fins opposite one another

2 anal shifted posteriorly relative to dorsal

252. Median fins (except caudal fin)

(Gardiner et al., 2005; Xu & Gao, 2011; Xu et al., 2014, 2015; Xu & Zhao, 2016; Giles et al. 2017; López-Arbarello & Sferco 2018.)

0 rays more numerous than radials

1 rays and radials equal

253. Proximal and middle radials of dorsal fin

(Giles et al. 2017.)

0 proximal and middle radials of similar size

1 proximal radials substantially enlarged

254. Posteriormost proximal radial of dorsal fin

(Giles et al. 2017)

0 enlarged plate

1 smaller than more anterior radials

255. Epichordal lobe of caudal fin

(Patterson, 1982; Cloutier & Ahlberg, 1996; Coates, 1999; Schultze & Cumbaa, 2001; Zhu & Schultze, 2001; Friedman & Blom, 2006; Long et al., 2008; Swartz, 2009; Choo, 2011; Giles et al., 2015b; Giles et al. 2017.)

0 present

1 absent

256. Fulcra along dorsal ridge of caudal fin

(Patterson, 1982; Taverne, 1997; Gardiner & Schaeffer, 1989; Gardiner et al., 2005; Friedman & Blom, 2006; Long et al., 2008; Choo, 2011; López-Arbarello 2012; Bermúdez-Rochas & Poyato-Ariza 2015; Giles et al., 2015b; Giles et al. 2017; López-Arbarello & Sferco 2018.)

0 absent

1 present

257. Caudal fin geometry

(Modified from Gardiner et al., 2005; Giles et al. 2017. A long chordal lobe is considered to be present when the notochord reaches the posterior margin of the caudal fin.)

0 long chordal lobe

1 short chordal lobe

258. Posterior margin of caudal fin

(Grande & Bemis 1998; Xu & Gao 2011; Xu et al. 2014; Xu & Zhao, 2016; Giles et al. 2017; López-Arbarello & Sferco 2018.)

0 forked

1 unforked

259. Diplospondyly in mid-caudal region

(Grande & Bemis 1998; Arratia 2013; Poyato-Ariza 2015; Giles et al. 2017; López-Arbarello & Sferco 2018.)

0 absent

1 present

260. Median neural spines in caudal region

(Coates 1999; Hurley et al. 2007; Xu et al. 2014; Giles et al. 2017; López-Arbarello & Sferco 2018.)

0 absent

1 present

261. Uroneural

(Pinna, 1996; Hurley et al., 2007; Xu & Wu, 2012, Xu et al., 2014; Xu & Zhao, 2016; Giles et al. 2017; López-Arbarello & Sferco 2018.)

0 absent

1 present

262. Division of hypurals into dorsal and ventral groups

(Pinna, 1996, Xu & Wu, 2012, Xu et al., 2014; Xu & Zhao, 2016; Giles et al. 2017.)

0 absent

1 present

263. Number of caudal lepidotrichs borne per hypural

(Giles et al. 2017; López-Arbarello & Sferco 2018.)

0 multiple

1 single

Axial skeleton

264. Opistocoelous vertebrae

(Wiley, 1976; López-Arbarello 2012; Bermúdez-Rochas & Poyato-Ariza 2015; Thies & Waschkewitz, 2015; Poyato-Ariza 2015; Gibson 2016; Giles et al. 2017; López-Arbarello & Sferco 2018.)

0 absent

1 present

265. Ossified ribs

(Giles et al. 2017.)

0 present

1 absent

New characters

266. Antorbital deeper than anteriormost infraorbital

(López-Arbarello 2012; Thies & Waschkewitz, 2015; Gibson 2016; López-Arbarello & Sferco 2018.)

0 absent

1 present

267. Infraorbitals fragmented

(The infraorbitals normally form as a single row partially surrounding the orbit. In some taxa, they may be fragmented into multiple irregular ossifications.)

0 absent

1 present

268. Suborbitals fragmented

(Grande & Bemis 1998; López-Arbarello 2011; López-Arbarello & Sferco 2018. The suborbitals normally form as a single row partially surrounding the orbit, posterior and/or ventral to the infraorbitals. In some taxa, they may be fragmented into multiple irregular ossifications.)

0 absent

1 present

269. Suborbitals extend ventral to orbit

(López-Arbarello 2011; López-Arbarello & Sferco 2018.)

0 absent

1 present

270. Coronoids contribute to lateral dentition field

(Coronoids are normally restricted to the medial (lingual) surface of the jaw. In some dapediids, they may extend laterally to contribute to the labial tooth row.)

0 absent

1 present

271. Jaw articulation ventral or anterior to orbit

(López-Arbarello 2012; Bermúdez-Rochas & Poyato-Ariza 2015; Poyato-Ariza 2015; Thies & Waschkewitz, 2015; Gibson 2016; López-Arbarello & Sferco 2018.)

0 absent

1 present

272. Parasphenoid wings around basiocciput

(The parasphenoid is normally restricted to covering the ventral face of the braincase. In some taxa, extensions may extend dorsally and laterally to cloak the basiocciput.)

0 absent

1 present

273. Dorsal extension of parasphenoid between orbits

(The parasphenoid extends dorsally between the orbits, contributing to the interorbital septum, in some taxa.)

0 absent

1 present

274. Ligament pit on posterior face of braincase

(A deep ligament pit, dorsal to the foramen magnum, is found on the posterior face of the braincase in some taxa.)

0 absent

1 present

275. 'Hem-like' median fins

(Gibson 2016.)

0 absent

1 present

276. Anal fin insertion

0 short based

1 long based

277. Denticulate ridge scales

0 absent

1 present

278. Ossified centra

(Poyato-Ariza & Wenz, 2002; Cawley & Kriwet 2018.)

0 absent

1 present

279. Supratemporal commissure in parietal

(Poyato-Ariza & Wenz, 2002; Thies & Waschkewitz, 2015; Gibson 2016; Cawley & Kriwet 2018; López-Arbarello & Sferco 2018. The supratemporal commissure is normally carried by the extrascapulars, but is sometimes borne in the parietal.)

0 absent

1 present

280. Enlarged vomerine teeth

(Poyato-Ariza & Wenz, 2002; Cawley & Kriwet 2018; López-Arbarello & Sferco 2018.)

0 absent

1 present

281. Enlarged prearticular teeth

(Poyato-Ariza & Wenz, 2002; Cawley & Kriwet 2018.)

0 absent

1 present

282. Prearticular symphysis

(Nursall 2010.)

0 absent

1 present

283. Dermal supraoccipital

(Poyato-Ariza & Wenz, 2002; Cawley & Kriwet 2018. A median bone, typically referred to as a dermal supraoccipital, is present between the parietals in some taxa.)

0 absent

1 present

284. Preoperculum taller than operculum

(Poyato-Ariza & Wenz, 2002; Cawley & Kriwet 2018.)

0 absent

1 present

285. Preoperculum wider than operculum

(Poyato-Ariza & Wenz, 2002; Cawley & Kriwet 2018.)

0 narrower

1 wider

286. Branchiostegals contact next in sequence

(Poyato-Ariza & Wenz, 2002; Cawley & Kriwet 2018.)

0 absent

1 present

287. Dorsal lateral line

0 absent

1 present

288. Parasphenoid inflected ventrally below orbit

(Nursall 2010.)

0 absent

1 present

289. Sagittal flange on neural spine

(Poyato-Ariza & Wenz, 2002; Cawley & Kriwet 2018.)

0 absent

1 present

290. Sagittal flange on haemal spine

(Poyato-Ariza & Wenz, 2002; Cawley & Kriwet 2018.)

0 absent

1 present

291. Pleural ribs alate

(Nursall 2010.)

0 absent

1 present

4) List of Taxa

**Taxon**	Source
Acanthodes bronni	Miles, 1973; Davis et al., 2012.
Acipenser brevirostrum	Nieuwenhuys, 1982; Hilton et al., 2011.
Aesopichthys erinaceus	Poplin & Lund, 2000.
Aetholepis mirablis	Woodward 1895.
Amia calva	Grande & Bemis, 1998; Sallan, 2014.
Amphicentrum granulosum	Traquair, 1875; Bradley-Dyne, 1939.
Arduafrons prominoris	Nursall 1999.
Atractosteus spatula	Grande, 2010.
Australosomus kochi	Nielsen, 1949.

Beagiascus pulcherrimus	Mickle et al., 2009.
Beishanichthys brevicaudalis	Xu & Gao, 2011.
Birgeria groenlandica	Nielsen, 1949.
Bobasatrania groenlandica	Stensiö, 1932.
Boreosomus piveteaui	Nielsen, 1942.
Brembodus ridens	Tintori 1981.
Caturus furcatus	Patterson, 1975; Lambers, 1994; Grande & Bemis, 1998.
Cheirolepis canadensis	Arratia & Cloutier, 1996; Arratia, 2009.
Cheirolepis schultzei	Arratia & Cloutier, 2004.
Cheirolepis trailli	Pearson & Westoll, 1979; Giles et al., 2015a.
Chondrosteus acipenseroides	Hilton & Forey 2009; Hilton et al., 2011.
Cladodoides wildungensis	Maisey, 2005.
Coccocephalichthys wildi	Poplin, 1974; Poplin & Véran, 1996.
Cosmoptychius striatus	Schaeffer, 1971; Coates, 1999.
Cyranorhis bergeraci	Lund et al., 1997.
Dandya ovalis	Tintori 1983.
Dapedium caelatum	Thies & Hauff, 2008; Thies & Herzog, 1999.
Dapedium punctatum	Thies & Herzog, 1999; Thies & Hauff, 2011; Thies & Waschewitz, 2015.
Dapedium noricum	Tintori 1983.
Dapedium pholidotum	Patterson, 1975; Thies & Herzog, 1999; Thies et al., 2015.
Dapedium stollorum	Thies & Herzog, 1999; Thies & Hauff, 2011; Thies & Waschewitz, 2015.
Dapedium sp.	Rayner 1948; Patterson 1975.
Dialipina salguerioensis	Schultze, 1968; Schultze & Cumbaa, 2001.
Diplocercides kayseri	Stensiö, 1922a,b; Forey, 1996.
Dipteronotus ornatus	Bürgin, 1992.

Discoserra pectinodon	Lund, 2000; Hurley et al., 2007.
Donnrosenia schaefferi	Long et al., 2008.
Dorsetichthys bechei	Patterson, 1968; Patterson, 1973; Patterson, 1975; Grande & Bemis, 1998.
Ebenaqua ritchei	Campbell & Le Duy Phuoc, 1983.
Elops hawaiensis	Forey, 1973.
Entelognathus primordialis	Zhu et al., 2013.
Eomesodon liassicus	Gardiner 1960.
Erpetoichthys calabaricus	Claeson et al., 2007; Claeson & Hagadorn, 2008.
Eusthenopteron foordi	Jarvik, 1980.
Evenkia eunoptera	Berg, 1942; Selenzneva, 1985; Sytchevskaya, 1999.
Fouldenia ischiptera	Sallan & Coates, 2013.
Fukangichthys longidorsalis	Xu et al., 2014.
Gibbodon cenensis	Tintori 1981.
Glyptolepis groenlandica	Jarvik, 1972; Jarvik, 1980.
Gogonasus andrewsae	Long et al., 1997; Long et al., 2006; Holland, 2014.
Gogosardina coatesi	Choo et al., 2009.
Guiyu oneiros	Zhu et al., 2009; Qiao & Zhu, 2010.
Hemicalypterus weiri	Schaeffer, 1967; Gibson 2013, 2016.
Heterostrophus phillipsi	Woodward 1928, 1929.
Hiodon alosoides	Hilton, 2002.
Howqualepis rostridens	Long, 1988; Choo, 2009.
Hulettia americana	Schaeffer & Patterson, 1984.
Ichthyokentema purbeckensis	Griffith & Patterson, 1963.
Kalops monophyrum	Poplin & Lund 2000.
Kansasiella eatoni	Poplin, 1974.
Kentuckia deani	Rayner, 1951; Giles & Friedman, 2014.
Lawrenciella schaefferi	Poplin, 1984; Hamel & Poplin, 2008.

Lepisosteus osseus	Balfour & Parker, 1882; Mathiesen & Popper, 1987; Grande, 2010.
Leptolepis bronni	Rayner, 1937.
"Ligulalepis"	Basden et al., 2000; Basden & Young, 2001.
Luederia kempi	Schaeffer & Dalquest, 1978.
Luganoia lepidosteoides	Bürgin, 1992.
Macrepistius arenatus	Schaeffer, 1960; Schaeffer, 1971.
Macrosemimimus lennieri	Jain & Robinson, 1963; Wenz, 1967; Patterson, 1875; Schröder et al., 2012.
Macrosemius rostratus	Bartram, 1977.
Meemannia eos	Zhu et al., 2006, 2010; Lu et al. 2016.
Melanecta anneae	Coates, 1998.
Mesturus sp.	Nursall 1999
Mesturus verrucosus	Nursall 1999
Mesopoma planti	Coates, 1999.
Miguashaia bureaui	Cloutier, 1996.
Mimipiscis bartrami	Gardiner, 1984; Choo, 2011.
Mimipiscis toombsi	Gardiner, 1984; Choo, 2011; Giles & Friedman, 2014.
Moythomasia lineata	Jessen, 1968; Choo 2015.
Moythomasia nitida	Jessen, 1968; Choo 2015.
Moythomasia durgaringa	Gardiner, 1984; Long & Trinajstic, 2010; Choo 2015.
Obaichthys decoratus	Grande, 2010.
Onychodus jandemarrai	Andrews et al., 2006.
Osorioichthys marginis	Taverne, 1997.
Osteolepis macrolepidotus	Jarvik 1948.
Ozarcus mapesae	Pradel et al., 2014.
Paradapedium egertoni	Jain 1973.
Peltopleurus lissocephalus	Bürgin, 1992.
Platysomus superbus	Moy-Thomas & Bradley-Dyne, 1938.

Polypterus bichir	Allis 1922; Jollie 1984; Bjerring 1991; Bartsch & Gemballa 1992; Bartsch et al. 1997; Grande 2010.
Porolepis sp.	Jarvik, 1972; Jarvik, 1980.
Propterus elongatus	Bartram, 1977.
Psarolepis romeri	Yu, 1998; Zhu et al., 1999; Zhu & Yu, 2009; Qu et al., 2013.
Pteronisculus stensioi	Nielsen 1942; Coates, 1998.
Raynerius splendens	Giles et al., 2015b.
Sargodon tomicus	Tintori 1983.
Saurichthys madagascarensis	Kogan et al., 2016.
Scanilepis dubia	Aldinger, 1937; Lehman, 1979.
Semionotus elegans	Olsen & McCune, 1991.
Styloichthys changae	Zhu & Yu, 2002; Zhu et al., 2006; Friedman, 2007.
Styracopterus fulcratus	Sallan & Coates, 2013.
Tanaocrossus kalliokoskii	Schaeffer, 1967; Schaeffer & Donald, 1978.
Tegeolepis clarki	Gardiner, 1963; Dunkle & Schaeffer, 1973.
Tetragonolepis oldhami	Jain 1973.
Tetragonolepis semicincta	Thies, 1991.
Venusichthys comptus	Xu & Zhao, 2016.
Watsonulus eugnathoides	Olsen, 1984; Grande & Bemis, 1998.
Wendyichthys dicksoni	Lund & Poplin, 1997.
Woodichthys bearsdeni	Coates, 1998.

5) Supplementary References

AHLBERG, P. E. and J. A. CLACK. 1998. Lower jaws, lower tetrapods - a review based on the Devonian genus *Acanthostega*. *Transactions of the Royal Society of Edinburgh: Earth Sciences* **88**: 11–46.

AHLBERG, P. E. and JOHANSON, Z. 1998. Osteolepiforms and the ancestry of tetrapods.

Nature **395**: 792–794.

ALDINGER, H. 1937. Permische Ganoidfisch aus Ostgrönland. *Meddelelser om Grønland* **102**: 1–392.

ALLIS, E. P. 1922. The cranial anatomy of *Polypterus*, with special reference to *Polypterus bichir*. *Journal of Anatomy* **56**: 189–294.

ANDREWS, S. M., LONG JA, AHLBERG P.E., BARWICK R. and CAMPBELL, K. 2006. The structure of the sarcopterygian *Onychodus jandemarrai* n. sp. from Gogo, Western Australia: with a functional interpretation of the skeleton. *Transactions of the Royal Society of Edinburgh* **96**: 197–307.

ARRATIA, G. 2009. Identifying patterns of diversity of the actinopterygian fulcra. *Acta Zoologica*, *Supplement* **90**: 220–235.

ARRATIA, G. and CLOUTIER, R. 1996. Reassessment of the morphology of *Cheirolepis canadensis* (Actinopterygii). 165–197. *In* SCHULTZE, H.-P. and CLOUTIER, R. (eds). *Devonian fishes and plants of Miguasha, Quebec, Canada*. Verlag Dr. Frederich Pfeil, Munich, 374 pp.

BALFOUR F. M. and PARKER, W.N. 1882. On the structure and development of *Lepisosteus*. *Philosophical Transactions of the Royal Society, London* **2**: 359–442.

BARTRAM, A. W. H. 1977. The Macrosemiidae, a Mesozoic family of holostean fishes. *Bulletin of the British Museum of Natural History (Geology)* **29**: 137–234 .

BARTSCH, P. and GEMBALLA, S. 1992. On the anatomy and development of the vertebral column and pterygiophores in *Polypterus senegalus* Cuvier, 1829 («Pisces», Polypteriformes). *Zoologische Jahrbücher. Abteilung für Anatomie und Ontogenie der Tiere*, **122**: 497–529.

BARTSCH. P., GEMBALLA. and PIOTROWSKI, T. 1997. The embryonic and larval development of *Polypterus senegalus* Cuvier, 1829: its staging with reference to

external and skeletal features, behaviour and locomotory habits. *Acta Zoologica, Stockholm* **78**: 309–328.

BASDEN, A. M. and YOUNG, G. C. 2001. A primitive actinopterygian neurocranium from the Early Devonian of southeastern Australia. *Journal of Vertebrate Paleontology*, **21**: 754–766.

BASDEN, A. M., YOUNG, G. C., COATES, M. I. and RITCHIE, A. 2000. The most primitive osteichthyan braincase? *Nature*, **403**: 185–188.

BERG, L. S. 1941. Lower Triassic fishes of the Tunguska Coal basin, Yenisei, Siberia. *Izvestiya Akademii Nauk SSSR (Seriya. Biologicheskaya)* **1941**: 458–474.

BERMÚDEZ-ROCHAS, D. D. and POYATO-ARIZA, F. J. 2015. A new semionotiform actinopterygian fish from the Mesozoic of Spain and its phylogenetic implications. *Journal of Systematic Palaeontology* **13**: 265–285.

BJERRING, H. C. 1991. Two intracranial ligaments supporting the brain of the brachiopterygian fish *Polypterus senegalus*. *Acta Zoologica*, *Stockholm* **72**: 41–47.

BÜRGIN, T. 1992. Basal ray-finned fishes (Osteichthyes; Actinopterygii) from the Middle Triassic of Monte San Giorgio (Canton Tessin, Switzerland). *Schweiserische Paläontologische Abhandlungen* **114:** 1-164.

BRADLEY DYNE, M. 1939. The skull of *Amphicentrum granulosum*. *Proceedings of the Zoological Society, London B* **109**: 195–210.

BRAZEAU, M. D. 2009. The braincase and jaws of a Devonian 'acanthodian' and modern gnathostome origins. *Nature* **457**: 305–308.

BRAZEAU, M. D. and FRIEDMAN, M. 2014. The characters of Palaeozoic jawed vertebrates. *Zoological Journal of the Linnean Society* **170**: 779–821.

CAMPBELL, K. S. W. and PHOUC, L. D. 1983. A late Permian actinopterygian fish from Australia. *Journal of Palaeontology* **26**: 33–70.

CAVIN, L. and SUTEETHORN, V. 2006. A new Semionotiform (Actinopterygii, Neopterygii) from Upper Jurassic–Lower Cretaceous deposits of North-East Thailand, with comments on the relationships of Semionotiforms. *Palaeontology* **49**: 339–353.

CHOO, B. 2011. Revision of the actinopterygian genus *Mimipiscis* (=*Mimia*) from the Upper Devonian Gogo Formation of Western Australia and the interrelationships of the early Actinopterygii. *Earth and Environmental Science Transactions of the Royal Society of Edinburgh* **102**: 77–104.

CHOO, B., LONG, J. A. and TRINAJSTIC, K. 2009. A new genus and species of basal actinopterygian fish from the Upper Devonian Gogo Formation of Western Australia. *Acta Zoologica*, *Supplement* **90**: 194–210.

CLAESON, K., BEMIS, W. E. and HAGADORN, J. W. 2007. New interpretations of the skull of a primitive bony fish *Erpetoichthys calabaricus* (Actinopterygii: Cladistia). *Journal of Morphology* **268**: 1021–1039.

CLAESON, K. M. and HAGADORN, J. W. 2008. The occipital region in the basal bony fish *Erpetoichthys calabaricus* (Actinopterygii: Cladistia). *Journal of Fish Biology* **73**: 1075–1082.

CLOUTIER, R. and AHLBERG, P. E. 1996. Morphology, characters, and the interrelationships of basal sarcopterygians. 445–479. *In* (STIASSNY, M. L. J., PARENTI, L. R. and JOHNSON, G. D. (eds) *Interrelationships of Fishes.* Academic Press, 496 pp.

CLOUTIER, R. 1996. The primitive actinistian *Miguashaia bureaui* Schultze (Sarcopterygii). 227–247. *In* SCHULTZE, H.P., and CLOUTIER, R. (eds) *Devonian Fishes and Plants of Miguasha, Quebec, Canada.* Verlag Dr. Friedrich Pfeil, Munich, 374 pp.

CLOUTIER, R. and ARRATIA, G. 2004. Early diversification of actinopterygians. 217–270. *In* ARRATIA, G., WILSON, M. V. H. and CLOUTIER, R. (eds). *Recent advances in*

the origin and early radiation of vertebrates. Verlag Dr. Friedrich Pfeil, Munich, 703 pp.

COATES, M. I. 1998. Actinopterygians from the Namurian of Bearsden, Scotland, with comments on early actinopterygian neurocrania. *Zoological Journal of the Linnean Society* **122**: 27–59.

COATES, M. I. 1999. Endocranial preservation of a Carboniferous actinopterygian from Lancashire, UK, and the interrelationships of primitive actinopterygians. *Philosophical Transactions of the Royal Society of London B* **354**: 435–462.

DAESCHLER, E. B., SHUBIN, N. H. and JENKINS, F. A. 2006. A Devonian tetrapod-like fish and the evolution of the tetrapod body plan. *Nature* **440**: 757–763.

DAVIS, S. P., FINARELLI, J. A. and COATES, M. I. 2012. *Acanthodes* and shark-like conditions in the last common ancestor of modern gnathostomes. *Nature* **486**: 247–250.

DIETZE, K. 2000. A revision of paramblypterid and amblypterid actinopterygians from Upper Carboniferous–Lower Permian lacustrine deposits of Central Europe. *Palaeontology* **43**: 927–966.

DUNKLE, D. H. 1939. A new paleoniscid fish from the Texas Permian. *American Journal of Science* **237**: 262–274.

FRIEDMAN, M. 2007. *Styloichthys* as the oldest coelacanth: implications for early osteichthyan interrelationships. *Journal of Systematic Palaeontology* **5**: 289–343.

FRIEDMAN, M. and BLOM, H. 2006. A new actinopterygian from the Famennian of East Greenland and the interrelationships of Devonian ray-finned fishes. *Journal of Paleontology*, **80**: 1186–1204.

FRIEDMAN, M. and BRAZEAU, M. D. 2010. A reappraisal of the origin and basal radiation of the Osteichthyes. *Journal of Vertebrate Paleontology* **30**: 36–56.

FOREY, P.L. 1973. A revision of the elopiform fishes, fossil and recent (Vol. 10). British Museum (Natural History). *Bulletin of the British Museum of Natural History (Geology)* **10**: 1–222 .

FOREY, P. L. 1980. *Latimeria*: a paradoxical fish. *Proceedings of the Royal Society of London B: Biological Sciences* **208**: 369–384.

FOREY, P. L. 1998. *History of the coelacanth fishes.* Chapman & Hall, London, 419 pp.

GARDINER, B. G. 1960. A revision of certain actinopterygian and coelacanth fishes, chiefly from the Lower Lias. *Bulletin of the British Museum (Natural History): Geology* **4**: 239–384.

GARDINER, B. G. 1963. Certain palaeoniscoid fishes and the evolution of the snout in actinopterygians. *Bulletin of the British Museum (Natural History): Geology* **8**: 254–325.

GARDINER, B. G. 1984. The relationships of the palaeoniscid fishes, a review based on new specimens of *Mimia* and *Moythomasia* from the Upper Devonian of Western Australia. *Bulletin of the British Museum (Natural History): Geology* **37**: 173–428.

GARDINER, B.G. and SCHAEFFER, B. 1989. Interrelationships of lower actinopterygian fishes. *Zoological Journal of the Linnaean Society* **97**: 135–187.

GARDINER, B. G., SCHAEFFER, B. and MASSERIE, J. A. 2005. A review of lower actinopterygian phylogeny. *Zoological Journal of the Linnean Society* **144**: 511–525.

GIBSON, S. Z. 2012. A new hump-backed ginglymodian fish (Neopterygii, Semionotiformes) from the Upper Triassic Chinle Formation of southeastern Utah. *Journal of Vertebrate Paleontology* **33**: 1037–1050.

GIBSON, S. Z. 2016. Redescription and phylogenetic placement of †*Hemicalypterus weiri* Schaeffer, 1967 (Actinopterygii, Neopterygii) from the Triassic Chinle Formation,

southwestern United States: new insights into morphology, ecological niche, and phylogeny. *PloS one* 11, p.e0163657.

GILES, S. and FRIEDMAN, M. 2014. Virtual reconstruction of endocast anatomy in early ray-finned fishes (Osteichthyes, Actinopterygii). *Journal of Paleontology* **88**: 636–651.

GILES, S., COATES, M. I., GARWOOD, R. J., BRAZEAU, M. D., ATWOOD, R., JOHANSON, Z. and FRIEDMAN, M. 2015a. Endoskeletal structure in *Cheirolepis* (Osteichthyes, Actinopterygii), an early ray-finned fish. *Palaeontology* **58**: 849–870.

GILES, S., DARRAS, L., CLÉMENT, G., BLIECK, A. and FRIEDMAN, M. 2015b. An exceptionally preserved Late Devonian actinopterygian provides a new model for primitive cranial anatomy in ray-finned fishes. *Proceedings of the Royal Society B*. **282**, 20151485.

GILES, S., FRIEDMAN, M. and BRAZEAU, M. D. 2015c. Osteichthyan-like cranial conditions in an Early Devonian stem gnathostome. *Nature*, **520,** 82–85.

GILES, S., XU, G.H., NEAR, T. J. and FRIEDMAN, M. 2017. Early members of 'living fossil' lineage imply later origin of modern ray-finned fishes. *Nature* **549**: 265–269.

GRANDE, L. 2010. An empirical synthetic pattern study of gars (Lepisosteiformes) and closely related species, based mostly on skeletal anatomy. The resurrection of Holostei. *American Society of Ichthyologists and Herpetologists, Special Publication* **6**: 1–871.

GRANDE, L. and BEMIS, W. E. 1998. A comprehensive phylogenetic study of amiid fishes (Amiidae) based on comparative skeletal anatomy. An empirical search for interconnected patterns of natural history. *Memoir (Society of Vertebrate Paleontology)* **4**: 1–690.

GRIFFITH, J. and PATTERSON, C. 1963. The structure and relationships of the Jurassic fish *Ichthyokentema purbeckensis. Bulletin of the British Museum of Natural History (Geology)*, **8**: 1–43.

HAMEL, M.-H. and POPLIN, C. 2008. The braincase anatomy of *Lawrenciella schaefferi*, actinopterygian from the Upper Carboniferous of Kansas (USA). *Journal of Vertebrate Paleontology* **28**: 989–1006.

HILTON, E. J. 2002. Osteology of the extant North American fishes of the genus *Hiodon* Lesueur, 1818 (Teleostei: Osteoglossomorpha: Hiodontiformes). *Fieldiana* **100**: 1−142.

HILTON, E. J. and FOREY, P. L. 2011. Redescription of *Chondrosteus acipenseroides* Egerton, 1858 (Acipenseriformes, Chondrosteidae) from the Lower Lias of Lyme Regis (Dorset, England), with comments on the evolution of sturgeon and paddlefishes. *Journal of Systematic Palaeontology* **7**: 427–453.

HILTON, E. J., GRANDE, L. and BEMIS, W. E. 2011. Skeletal anatomy of the shortnose sturgeon, *Acipenser brevirorostrum* Lesueur 1818, and the systematics of sturgeons (Acipenseriformes, Acipenseridae). *Fieldiana (Life and Earth Sciences)* **3**: 1–168.

HOLLAND, T. 2014. The endocranial anatomy of *Gogonasus andrewsae* Long, 1985 revealed through micro CT-scanning. *Earth and Environmental Science Transactions of the Royal Society of Edinburgh* **105**: 9–34.

HURLEY, I. A., MUELLER, R. L., DUNN, K., SCHMIDT, E., FRIEDMAN, M., HO, R. K., PRINCE, V. E., YANG, Z., THOMAS, M. G. and COATES, M. I. 2007. A new timescale for ray-finned fish evolution. *Proceedings of the Royal Society B* **274**: 489–498.

JAIN, S. L. 1973. New specimens of Lower Jurassic holostean fishes from India. *Palaeontology* **16**: 149–177.

JAIN, S.L. and ROBINSON, P. L. 1963. Some new specimens of the fossil fish Lepidotes from the English Upper Jurassic. In Proceedings of the Zoological Society of London (Vol. 141, No. 1, pp. 119-135). Blackwell Publishing Ltd.

JARVIK, E. 1948. On the morphology and taxonomy of the Middle Devonian osteolepid fishes of Scotland. *Kungliga Svenska Vetenskapsakademiens Handligar* **25**: 1–30.

JARVIK, E. 1972. Middle and Upper Devonian Porolepiformes from East Greenland with special reference to *Glyptolepis groenlandica* n.sp. *Meddelelser om Grønland* **187**: 1–295.

JARVIK E. 1980. *Basic structure and evolution of vertebrates. Volume 1*. Academic Press, London, 575 pp.

JESSEN, H. 1968. *Moythomasia nitida* Gross und *M*. cf. *striata* Gross, Devonische palaeonisciden aus dem oberen Plattenkalk der Bergish-Gladbach-Paffrather Mulde (Rheinisches Schiefergebirge). *Palaeontographica Abteilung A* **128**: 87–114.

JESSEN, H. L. 1973. Interrelationships of actinopterygians and brachyopterygians: evidence from pectoral anatomy. 227–232. *In* GREENWOOD, P. H., MILES, R. S. and PATTERSON, C. (eds). *Interrelationships of fishes*. Academic Press, London, 536 pp.

JOLLIE, M. 1984. Development of the head and pectoral skeleton of *Polypterus* with a note on scales (Pisces: Actinopterygii). *Journal of Zoology* **204**: 469–507.

LAMBERS, P. H. 1994. The halecomorph fishes *Caturus* and *Amblysemius* in the lithographic limestone of Solnhofen (Tithonian), Bavaria. *Geobios* **27**: 91–99.

LAUDER, G. V. and LIEM, K. F. 1983. The evolution and interrelationships of the actinopterygian fishes. *Bulletin of the Museum of Comparative Zoology, Harvard University* **150**: 95–197.

LEHMAN, J. P. 1979. Le genre *Scanilepis* Aldinger du Rhétien de la Scanie (Suéde). *Bulletin of the Geological Institution of the University of Uppsala* **8**: 113–125

LONG, J. A. 1988. New palaeoniscoid fishes from the Late Devonian and early Carboniferous of Victoria. *Memoir of the Australasian Association of Palaeontologists* **7**: 1–64.

LONG, J. A., YOUNG, G. C., HOLLAND, T., SENDEN, T. J. and FITZGERALD, E. M. G. 2006. An exceptional Devonian fish from Australia sheds light on tetrapod origins. *Nature* **444**: 199–202.

LONG, J. A., CHOO, B. and YOUNG, G. C. 2008. A new basal actinopterygian from the Middle Devonian Aztec Siltstone of Antarctica. *Antarctic Science* **20**: 393–412.

LONG, J. A., TRINAJSTIC, K. 2010. The Late Devonian Gogo Formation Lägerstatte of Western Australia: exceptional early vertebrate preservation and diversity. *Annual Review of Earth and Planetary Sciences* **38**: 255–79.

LÓPEZ-ARBARELLO, A. 2012. Phylogenetic interrelationships of ginglymodian fishes (Actinopterygii: Neopterygii). *PLoS One* **7**, p.e39370.

LÓPEZ-ARBARELLO, A. and SFERCO, E. 2018. Neopterygian phylogeny: the merger assay. *Royal Society Open Science* **5**, p.172337.

LUND, R. 2000. The new actinopterygian order Guildayichthyiformes from the Lower Carboniferous of Montana (USA). *Geodiversitas* **22**: 171–206.

LUND, R. and POPLIN, C. 2002. Cladistic analysis of the relationships of the tarrasiids (Lower Carboniferous Actinopterygians). *Journal of Vertebrate Paleontology* **22**: 480–486.

LUND, R., POPLIN, C. and MCCARTHY, K. 1995. Preliminary analysis of the interrelationships of some Paleozoic actinopterygii. *Geobios* **28**: 215–220.

LUND, R. and POPLIN, C. 1997. The rhadinichthyids (paleoniscoid actinopterygians) from the Bear Gulch Limestone of Montana (USA, Lower Carboniferous). *Journal of Vertebrate Paleontology* **17**: 466–486.

KOGAN, I. and ROMANO, C. 2016. Redescription of *Saurichthys madagascariensis* Piveteau, 1945 (Actinopterygii, Early Triassic), with implications for the early saurichthyid morphotype. *Journal of Vertebrate Paleontology* p.e1151886.

MAISEY, J. G. 1986. Heads and tails: a chordate phylogeny. *Cladistics* **2**: 201–256.

MAISEY, J. G. 2005. Braincase of the Upper Devonian shark *Cladodoides wildungensis* (Chondrichthyes, Elasmobranchii), with observations on the braincase in early Chondrichthyans. *Bulletin of the American Museum of Natural History* **288**:1–103.

MATHIESEN, C. and POPPER, A.N. 1987. The ultrastructure and innervation of the ear of the gar, *Lepisosteus osseus. Journal of Morphology* **194**: 129–142.

MICKLE, K. E., LUND, R. and GROGAN, E. D. 2009. Three new palaeoniscoid fishes from the Bear Gulch Limestone (Serpukhovian, Mississippian) of Montana (USA) and the relationships of lower actinopterygians. *Geodiversitas* **31**: 623–668.

MILES, R. S. 1973. Relationships of acanthodians. 63–103. *In* GREENWOOD, P. H., MILES, R. S. and PATTERSON, C. (eds). *Interrelationships of fishes*. Academic Press, London, 536 pp.

MOY-THOMAS, J. A., and BRADLEY-DYNE, M. 1938. XVII.—The Actinopterygian Fishes from the Lower Carboniferous of Glencartholm, Eskdale, Dumfriesshire. *Transactions of the Royal Society of Edinburgh* **59**: 437–480.

NIELSEN, E. 1942. Studies on Trassic fishes from East Greenland I. *Glaucolepis* and *Boreosomus*. *Meddelelser om Grønland* **146**: 1–309.

NIELSEN, E. 1949. Studies on Triassic fishes from East Greenland. II. Australosomus and Birgeria. *Meddelser øm Grønland* **146**: 1–309.

NIEUWENHUYS, R. 1982. An overview of the organization of the brain of actinopterygian fishes. *American Zoologist* **22**: 287–310.

NURSALL J.R. 1999. The family †Mesturidae and the skull of the pycnodont fishes. 153–

188. *In* ARRATIA, G. and SCHULTZE, H.-P. (eds.) *Mesozoic Fishes 2: Systematics and Fossil Record*. Verlag Dr. Friedrich Pfeil, Munich, 604 pp.

OLSEN, P. E. 1984. The skull and pectoral girdle of the parasemionotid fish *Watsonulus eugnathoides* from the early Triassic Sakamena Group of Madagascar, with comments on the relationships of the holostean fishes. *Journal of Vertebrate Paleontology* **4**: 481 – 499.

OLSEN, P. E. and MCCUNE, A. R. 1991. Morphology of the *Semionotus elegans* species group from the Early Jurassic part of the Newark Supergroup of Eastern North America with comments on the family Semiotidae (Neopterygii). *Journal of Vertebrate Paleontology* **11**: 269–292.

PATTERSON, C. 1968. The caudal skeleton in Lower Liassic pholidophorid fishes. Bulletin of the British Museum Natural History (Geology) **16**: 203–239.

PATTERSON, C. 1973. Interrelationships of holosteans. 233–306. *In* P. H. GREENWOOD, P. H., MILES, R. S. and PATTERSON, C (eds). *Interrelationships of Fishes* Academic Press, New York, 536 pp.

PATTERSON, C. 1975. The braincase of pholidophorid and leptolepid fishes, with a review of the actinopterygian braincase. *Philosophical Transactions of the Royal Society of London, Series B* **269**: 275–579.

PATTERSON, C. 1982. Morphology and interrelationships of primitive actinopterygian fishes. *American Zoologist* **22**: 241–259.

PEARSON, D.M. and WESTOLL, T. S. 1979. The Devonian actinopterygian *Cheirolepis* Agassiz. *Transactions of the Royal Society of Edinburgh* **70**: 337–399.

POPLIN C. 1974. Étude de quelques Paléoniscidés pennsylvaniens du Kansas. *Cahiers de Paléontologie (Section Vertébrés),* Paris, 151 pp.

POPLIN, C. M. 1984. *Lawrenciella schaefferi* n. g., n. sp. (Pisces: Actinopterygii) and the

use of endocranial characters in the classification of the palaeonisciformes. *Journal of Vertebrate Paleontology* **4**: 413–421.

POPLIN, C. and LUND, R. 2000. Two new deep-bodied palaeoniscoid actinopterygians from Bear Gulch (Montana, USA, Lower Carboniferous). *Journal of Vertebrate Paleontology* **20**: 428–449.

POPLIN C. and VÉRAN, M. 1996. A revision of the actinopterygian fish *Coccocephalus wildi* from the Upper Carboniferous of Lancashire. *Special Papers in Palaeontology* **52**: 7–29.

POYATO-ARIZA, F. J. 2015. Studies on pycnodont fishes (I): evaluation of their phylogenetic position among actinopterygians. *Rivista Italiana di Paleontologia e Stratigrafia* **121**: 329–343.

PRADEL, A., MAISEY, J. G., TAFFOREAU, P., MAPES, R. H. and MALLATT, J., 2014. A Palaeozoic shark with osteichthyan-like branchial arches. *Nature* **509**: 608–611.

QIAO, T. and Zhu, M. 2010. Cranial morphology of the Silurian sarcopterygian *Guiyu oneiros* (Gnathostomata: Osteichthyes). *Science China Earth Sciences* **53**: 1836–1848.

QU, Q., ZHU, M. and WANG, W. 2013. Scales and dermal skeletal histology of an early bony fish *Psarolepis romeri* and their bearing on the evolution of rhombic scales and hard tissues. *PLoS ONE,* **8**, e61485.

RAYNER, D. H. 1937. II.—On *Leptolepis bronni* Agassiz. *Journal of Natural History* **19**: 46–74.

RAYNER, D. H. 1948. The structure of certain Jurassic holostean fishes, with special reference to their neurocrania. *Philosophical Transactions of the Royal Society of London B* **233**: 287–345.

RAYNER, D. H. 1951. On the cranial structure of an early palaeoniscid, *Kentuckia* gen. nov. *Transactions of the Royal Society of Edinburgh* **62**: 58–83.

SALLAN, L. C. 2014. Major issues in the origins of ray-finned fish (Actinopterygii) biodiversity. *Biological Reviews*, **89**: 950–971.

SALLAN, L. C. and COATES, M. I. 2013. Styracopterid (Actinopterygii) ontogeny and the multiple origins of post-Hangenberg deep-bodied fishes. *Zoological Journal of the Linnean Society* **169**: 156–199.

SCHAEFFER, B., COPE, E. D. and HILL, R. T. 1960. The Cretaceous holostean fish *Macrepistius*. *American Museum Novitates*; **2011**:1–18.

SCHAEFFER, B. 1967. Late Triassic fishes from the Western United States. *Bulletin of American Museum of Natural History* **135**: 285–342.

SCHAEFFER, B. 1971. The braincase of the holostean fish *Macrepistius*, with comments on neurocranial ossification in the Actinopterygii. *American Museum Novitates* **2459**: 1–34.

SCHAEFFER, B. and DALQUEST, W. W. 1978. A palaeonisciform braincase from the Permian of Texas, with comments on cranial fissures and the posterior myodome. *Novitates*: **2658**: 1–15.

SCHAEFFER, B. and DONALD, N. G. M. 1978. Redfieldiid fishes from the Triassic-Liassic Newark Supergroup of eastern North America. *Bulletin of the American Museum of Natural History* **159**: 131–173.

SCHAEFFER, B. and PATTERSON, C. 1984. Jurassic fishes from the western United States, with comments on Jurassic fish distribution. *American Museum Novitates* **2796**: 1–86.

SCHRÖDER, K. M., LÓPEZ-ARBARELLO, A. and EBERT, M. 2012. *Macrosemimimus* gen. nov. (Actinopterygii, Semionotiformes) from the Late Jurassic of Germany, England and France. *Journal of Vertebrate Paleontology* **32**: 512–529.

SCHULTZE, H. P. 1968. Palaeoniscoidea-Schuppen aus dem Unterdevon Australiens und

Kanadas und aus dem Mitteldevon Spitzbergens Bulletin of the British Museum (Natural History) **16**: 343–376.

SCHULTZE, H.-P and CUMBAA, S. L. 2001. *Dialipina* and the characters of basal osteichthyans. 315-332. *In* AHLBERG, P. E. (ed.) *Major Events in Early Vertebrate Evolution.* Taylor & Francis, London. 418 pp.

SELEZNEVA, A. A. 1985. *Evenkia* is *Polypterus* ancestor. *Paleotologisheskii Zhurnal* **19**: 71–76. [Russian]

STENSIÖ, E.A. 1922a. Über zwei Coelacanthiden aus dem Oberdevon van Wildungen. *Palaeontologischen Zeitschrift* **4**: 167–210.

STENSIÖ, E.A. 1922b. Notes on certain crossopterygians. *Proceedings of the Zoological Society of London*: **92**: 1241–1271.

STENSIÖ. 1932. Triassic fishes from East Greenland. *Meddelelser om Grønland* **83**: 125–164.

SWARTZ, B. A. 2009. Devonian actinopterygian phylogeny and evolution based on a redescription of *Stegotrachelus finlayi*. *Zoological Journal of the Linnean Society* **56**: 750–784.

SYTCHEVSKAYA, E. K. 1999. Freshwater fish fauna from the Triassic of Northern Asia. 445–468. *In* ARRATIA, G. and SCHULTZE, H.-P. (eds.) *Mesozoic Fishes 2: Systematics and Fossil Record*. Verlag Dr. Friedrich Pfeil, Munich, 604 pp.

TAVERNE, L. 1997. *Osorioichthys marginis*, “Paléonisciforme” du Famennien de Belgique, et la phylogénie de Actinoptérygiens dévoniens (Pisces). *Bulletin de l’Institut Royal des Sciences Naturelles de Belgique* **67**: 57–78.

THIES, D. 1991. The osteology of the bony fish *Tetragonolepis semicincta* Bronn 1830 (Actinopterygii, †Semionotiformes) from the Early Jurassic (Lower Toarcian) of Germany. *Geologica et Palaeontologica* **25**: 251– 297.

THIES, D. and HAUFF, R. B. 2008. A neotype for *Dapedium caelatum* Quenstedt, 1858 (Actinpoterygii, Neopterygii, Semionotiformes) from the Early Jurassic (Early Toarcian) of South Germany. *Geologica et Palaeontologica* **42**: 23–38.

THIES, D. and HAUFF, R. B. 2011. A new species of *Dapedium* Leach, 1822 (Actinopterygii, Neopterygii, Semionotiformes) from the Early Jurassic of South Germany. *Palaeodiversity* **4**: 185–221.

THIES, D. and HERZOG, A. 1999. New information on †*Dapedium* Leach 1822 (Actinopterygii, †Semionotiformes). 143–152 *In* ARRATIA, G. and SCHULTZE, H.-P. (eds.) *Mesozoic Fishes 2: Systematics and Fossil Record*. Verlag Dr. Friedrich Pfeil, Munich, 604 pp.

THIES, D. and WASCHKEWITZ, J. 2015. Redescription of *Dapedium pholidotum* (Agassiz, 1832) (Actinopterygii, Neopterygii) from the Lower Jurassic Posidonia Shale, with comments on the phylogenetic position of *Dapedium* Leach, 1822. *Journal of Systematic Palaeontology* **14**: 339–364.

TINTORI, A., 1981. Two new pycnodonts (Pisces, Actinopterygii) from the Upper Triassic of Lombardy. *Rivista Italiana di Paleontologia e Stratigrafia* **86**: 19–30.

TINTORI, A. 1983. Hypsiomatic Semionotidae (Pisces, Actinopterygii) from the Upper Triassic of Lombardy (N. Italy). *Rivista Italiana di Paleontologia e Stratigrafia* **88**: 417–442.

TRAQUAIR, R. H. 1877 – 1914. The Ganoid Formations, Palaeontographical Society Monograph, Fishes of the 1877 – 1914. British Carboniferous Palaeontographical Society, London.

WENZ, S. 1968. Compléments à l'étude des poissons actinoptérygiens du Jurassique français. *Centre National de la Recherche Scientifique* 1–276.

Woodward 1985, 1928, 1929.

XU, G.-H. and GAO, K.Q. 2011. A new scanilepiform from the Lower Triassic of northern Gansu Province, China, and phylogenetic relationships of non-teleostean Actinopterygii. *Zoological Journal of the Linnean Society* **161**: 595–612.

XU, G.-H., GAO, K.-Q. and FINARELLI, J. A. 2014. A revision of the Middle Triassic scanilepiform fish *Fukangichthys longidorsalis* from Xinjiang, China, with comments on the phylogeny of the Actinopteri. *Journal of Vertebrate Paleontology* **34**: 747–759.

XU, G.-H., GAO, K.-Q. and COATES, M. I. 2015. Taxonomic revision of *Plesiofuro mingshuica* from the Lower Triassic of northern Gansu, China, and the relationships of early neopterygian clades. *Journal of Vertebrate Paleontology* **35**: e1001515.

XU, G.-H. and SHEN, C.-C. 2015. *Panxianichthys imparilis* gen. et sp. nov., a new ionoscopiform (Halecomorphi) from the Middle Triassic of Guizhou, China. *Vertebrata PalAsiatica* **53**: 1–6.

XU, G.-H. and ZHAO, L.J. 2016. A Middle Triassic stem-neopterygian fish from China shows remarkable secondary sexual characteristics. *Science Bulletin* **61**:338–344.

XU, G.-H. & ZHAO, L.J. 2016. A Middle Triassic stem-neopterygian fish from China shows remarkable secondary sexual characteristics. *Scientific Bulletin* **61**:338–344.

YU, X. 1998. A new porolepiform-like fish, *Psarolepis romeri*, gen. et sp. nov. (Sarcopterygii, Osteichthyes) from the Lower Devonian of Yunnan, China. *Journal of Vertebrate Paleontology* **18**: 261–274.

ZHU, M., YU, X. and JANVIER, P . 1999. A primitive fossil fish sheds light on the origin of bony fishes. *Nature* **397**: 607–610.

ZHU, M. and AHLBERG, P. E. 2004. The origin of the internal nostril of tetrapods. *Nature* **432**: 94–97.

ZHU, M. & SCHULTZE, H. P. 2001. Interrelationships of basal osteichthyans. 289–314 *In* AHLBERG, P.E. (ed). *Major events in early vertebrate evolution: palaeontology, phylogeny, genetics and development*. Taylor & Francis, London, 418 pp.

ZHU, M., WANG, W. and YU, X. 2010. *Meemannia eos*, a basal sarcopterygian fish from the Lower Devonian of China–expanded description and significance. 199–214. *In* ELLIOT, D. K., MAISEY, J. G., YU, K. and MIAO, D. (eds). *Morphology, Phylogeny and Paleobiogeography of Fossil Fishes*. Verlag, Dr. Friedrich Pfeil, Munich, 472 pp.

ZHU, M. and YU, X. 2002. A primitive fish close to the common ancestor of tetrapods and lungfish. *Nature* **418**: 767–770.

ZHU, M., YU, X. and AHLBERG, P. E. 2001. A primitive sarcopterygian fish with an eyestalk. *Nature* **410**: 81–84.

ZHU, M., YU, X., AHLBERG, P. E., CHOO, B., LU, J., QIAO, T., QU, Q., ZHAO, W., JIA, L., BLOM, H. and ZHU, Y. 2013. A Silurian placoderm with osteichthyan-like marginal jaw bones. *Nature*, **502**:188–193.

ZHU, M., YU, X., WANG, W., ZHAO, W. and JIA, L . 2006. A primitive fish provides key characters bearing on deep osteichthyan phylogeny. *Nature* **441**: 77–80.

ZHU, M., ZHAO, W., JIA, L., LU, J., QIAO, T. and QU, Q. 2009. The oldest articulated osteichthyan reveals mosaic gnathostome characters. *Nature*, **458**: 469–474.
