# Supplementary material for: A giant dapediid from the Late Triassic of Switzerland and insights into neopterygian phylogeny
Source: R Soc Open Sci. 2018 Aug 15;5(8):180497. doi: 10.1098/rsos.180497 (PMC6124034; doi:10.1098/rsos.180497)
Supplement: Supplementary Figure 1 [file rsos180497supp3.pdf]

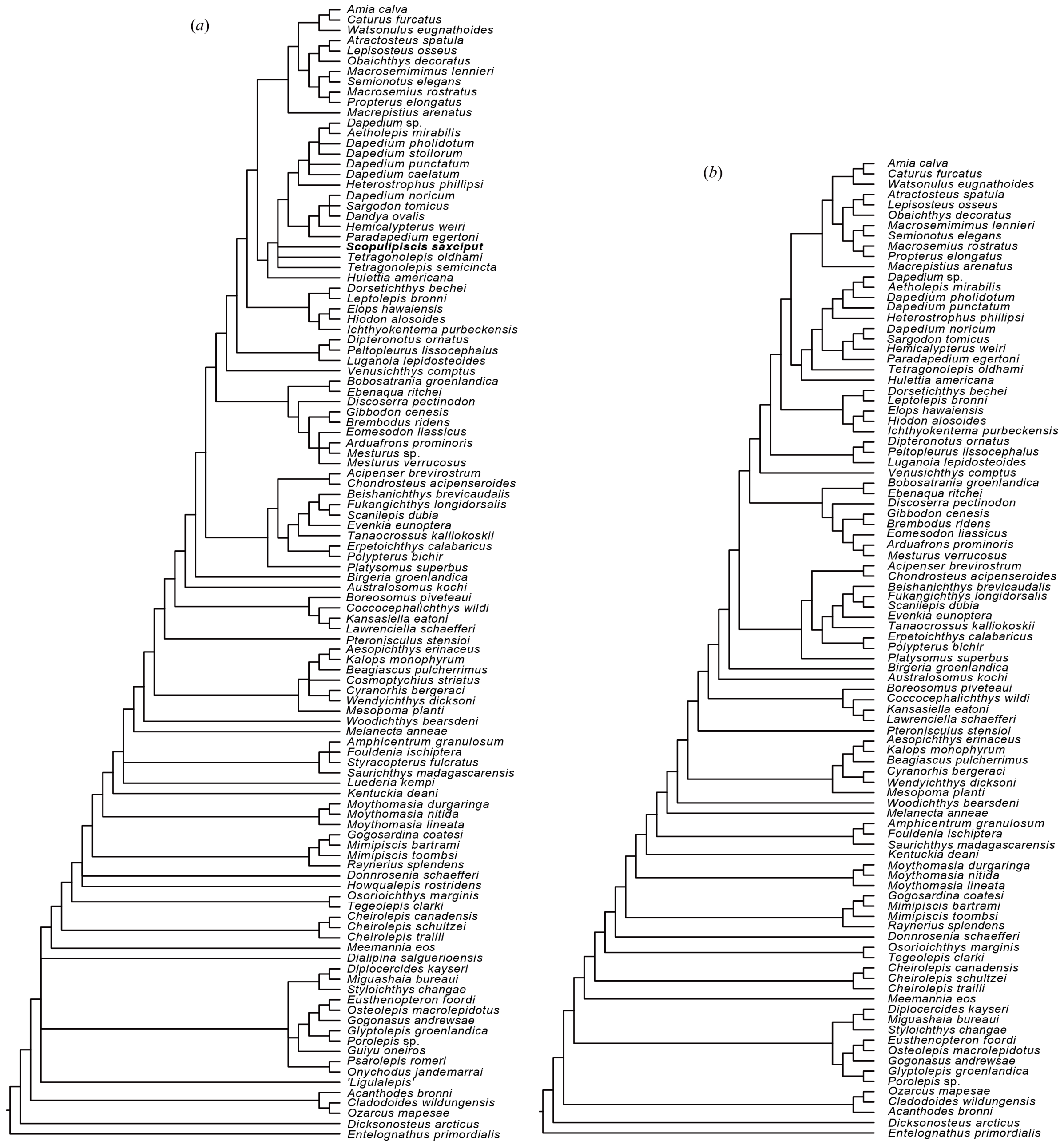

Supplementary Figure 1. Phylogenetic position of *Scopulipiscis saxciput* gen. et sp. nov. (a) Adams consensus tree of the 20000 shortest trees (1522 steps) for 110 taxa and 291 equally weighted characters. (b)Agreement subtree of the 20000 shortest trees (1522 steps) for 110 taxa and 291 equally weighted characters. 95 of the 110 taxa are included, and the following taxa are pruned from the tree: '*Ligulalepis*', *Dialipina salguerioensis*, *Onychodus jandemarrai*, *Guiyu oneiros*, *Psarolepis romeri*, *Howqualepis rostridens*, *Luederia kempi*, *Styracopterus fulcratus*, *Mesturus* sp., *Tetragonolepis semicincta*, *Scopulipiscis saxciput*, *Dapedium stollurum*, *Dapedium caelatum*, *Dandya ovalis*, *Cosmoptychius striatus*.
