# Supplementary material for: A giant dapediid from the Late Triassic of Switzerland and insights into neopterygian phylogeny
Source: R Soc Open Sci. 2018 Aug 15;5(8):180497. doi: 10.1098/rsos.180497 (PMC6124034; doi:10.1098/rsos.180497)
Supplement: Supplementary Data [file rsos180497supp4.pdf]

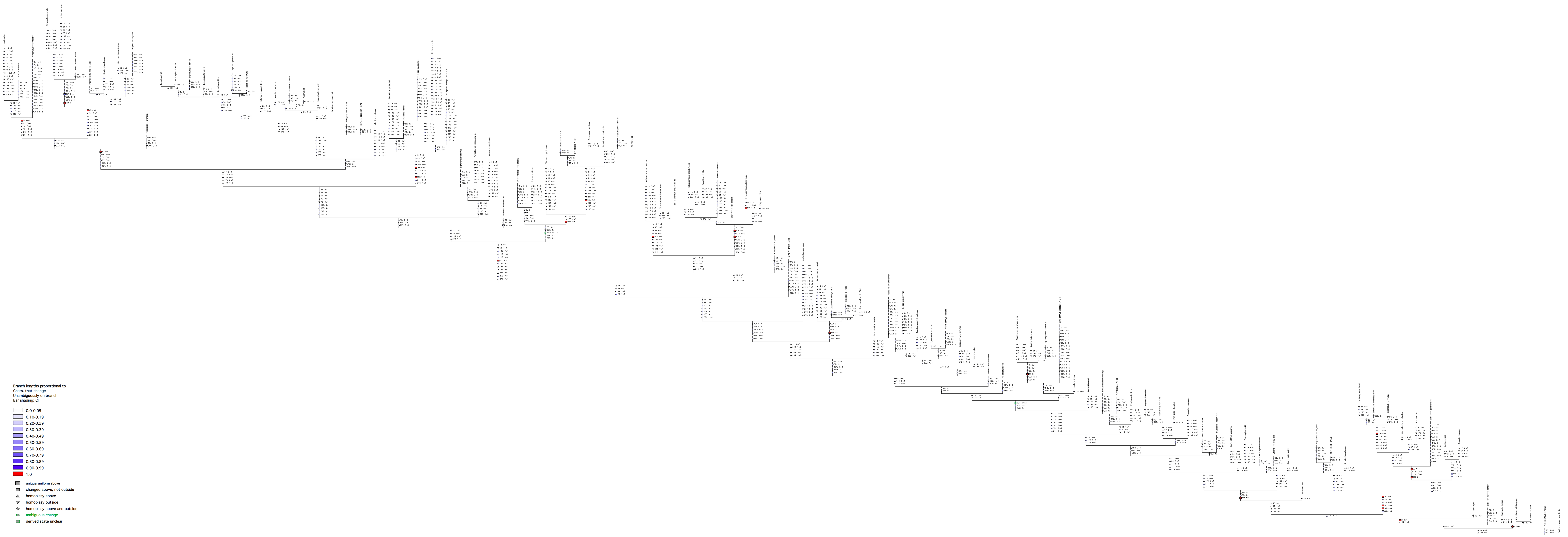
Branch lengths proportional to
Chars. that change
Unambiguously on branch
Bar shading: CI
0.0-0.09
0.10-0.19
0.20-0.29
0.30-0.39
0.40-0.49
0.50-0.59
0.60-0.69
0.70-0.79
0.80-0.89
0.90-0.99
1.0
unique, uniform above
changed above, not outside
homoplasy above
homoplasy outside
homoplasy above and outside
ambiguous change
derived state unclear
